# Protected Chaos in a Topological Lattice

**DOI:** 10.1002/advs.202503216

**Published:** 2025-05-20

**Authors:** Haydar Sahin, Hakan Akgün, Zhuo Bin Siu, S. M. Rafi‐Ul‐Islam, Jian Feng Kong, Mansoor B. A. Jalil, Ching Hua Lee

**Affiliations:** ^1^ Department of Electrical and Computer Engineering National University of Singapore Singapore 117583 Republic of Singapore; ^2^ Institute of High Performance Computing (IHPC) Agency for Science Technology and Research (A*STAR) Singapore 138632 Republic of Singapore; ^3^ Department of Physics Bilkent University Ankara 06800 Türkiye; ^4^ Quantum Innovation Centre (Q.InC) Agency for Science Technology and Research (A*STAR) 2 Fusionopolis Way, Innovis #08‐03 Singapore 138634 Republic of Singapore; ^5^ Department of Physics National University of Singapore Singapore 117542 Republic of Singapore

**Keywords:** machine learning, protected chaos, non‐linear topology, topological protection

## Abstract

The erratic nature of chaotic behavior is thought to erode the stability of periodic behavior, including topological oscillations. However, it is discovered that in the presence of chaos, non‐trivial topology not only endures but also provides robust protection to chaotic dynamics within a topological lattice hosting non‐linear oscillators. Despite the difficulty in defining topological invariants in non‐linear settings, non‐trivial topological robustness still persists in the parametric state of chaotic boundary oscillations. This interplay between chaos and topology is demonstrated by incorporating chaotic Chua's circuits into a topological Su‐Schrieffer‐Heeger (SSH) circuit. By extrapolating from the linear limit to deep into the non‐linear regime, it is found that distinctive correlations in the bulk and edge scroll dynamics effectively capture the topological origin of the protected chaos. The findings suggest that topologically protected chaos can be robustly achieved across a broad spectrum of periodically driven systems, thereby offering new avenues for the design of resilient and adaptable non‐linear networks.

## Graphical abstract

Topological and chaotic dynamics are often considered incompatible, with one expected to dominate or disrupt the other. This work reveals that topological localization can persist even under strong chaotic dynamics and, counter‐intuitively, protect chaotic behavior. The findings challenge the conventional view that chaos erodes regular oscillations, showing instead that it can be protected by topology in non‐linear dynamical lattices.

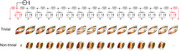

## 1 Introduction

Chaotic dynamics, despite being notoriously unpredictable and sensitive to initial conditions,^[^ ^1^ ^]^ govern the behavior of many complex systems^[^ ^2^, ^3^ ^]^ such as networks of coupled oscillators and dynamical non‐linear lattices.^[^ ^4^, ^5^, ^6^, ^7^, ^8^, ^9^ ^]^ In such coupled dynamical networks, even small perturbations are amplified rapidly, leading to complex global behaviors.^[^ ^4^, ^10^, ^11^ ^]^ While synchronization can appear at times, chaos, with its inherent unpredictability, disrupts this order and makes the system decidedly irregular in the long run. However, we discover that this well‐established pattern of behavior may no longer hold when a network of coupled chaotic oscillators is arranged in a topological manner. Despite the lack of well‐defined band topological invariants in nonlinear systems, we find that topological robustness potently persists, even as chaotic behavior introduces randomness that would erode the bulk periodicity and threaten any semblance of topological bulk‐boundary correspondence.

Surprisingly, not only can topological robustness be well‐preserved in certain chaotic systems, it even protects chaotic oscillations from strong perturbations, demonstrating a parametric robustness inherited from band topology. This protection stems from the stability of non‐trivial edge oscillations, whose absence would lead to the rapid decay of bulk chaotic attractors, as observed in the case of trivial edge oscillations.

Recent progress in non‐linear topological physics has expanded to include models combining non‐linear and non‐Hermitian mechanisms. Of particular interest are phenomena such as non‐Hermitian global synchronization, where the inherent non‐orthogonality of eigenmodes facilitates enhanced communication between modes, enabling global synchronization;^[^ ^12^ ^]^ size‐dependent non‐Hermitian topological synchronization, characterized by distinct edge and bulk oscillations, with bulk oscillation frequencies explicitly depending on lattice size;^[^ ^13^ ^]^ and a growth blockade arising from modulational instability observed in non‐Hermitian periodic Hatano‐Nelson lattices.^[^ ^14^ ^]^ Additionally, various types of non‐linear skin modes have recently been reported.^[^ ^15^, ^16^, ^17^ ^]^ Interestingly, while non‐linearity is conventionally considered disruptive to topological mechanisms, it is now intentionally utilized to configure these very topological states.^[^ ^18^ ^]^ Despite these exciting developments, existing studies predominantly consider non‐chaotic non‐linearities, employing regular oscillator models such as Stuart‐Landau or Kerr‐type oscillators.

In this work, we choose chaotic Chua circuits among various potential candidates to implement the Lorenz system,^[^ ^1^ ^]^ as they offer an experimentally accessible and easily tunable platform for investigating chaotic dynamics.^[^ ^19^, ^20^, ^21^ ^]^ Identical Chua circuits are coupled such that they form a one‐dimensional topological Su‐Schrieffer‐Heeger (SSH) circuit array with onsite chaotic Chua oscillators.^[^ ^22^, ^23^, ^24^, ^25^, ^26^, ^27^, ^28^, ^29^, ^30^, ^31^, ^32^ ^]^ In our setup, the degree of non‐linearity can be adjusted by tuning the current‐voltage characteristics of the Chua diodes, with high non‐linearity typically expected to substantially distort linear eigenstates.

In non‐linear systems, a key challenge in attributing robustness to band topology is the difficulty in defining bulk topological invariants, which are inherently properties of linear band structures. (Nonetheless, see Refs. [33, 34, 35, 36, 37, 38] for proposed non‐linear topological invariants in lattices with generally Kerr‐type non‐linearities.) To overcome this, we continuously deform the Chua circuit array from its linear SSH limit by tuning its non‐linear Chua diodes. Notably, the topological edge oscillations are preserved remarkably well even deep into the nonlinear regime. This allows for the identification of non‐linear topological boundaries, which can be made further precise with the aid of machine learning techniques.^[^ ^39^ ^]^

Importantly, we emphasize that although we shall demonstrate topologically protected chaos only with Chua circuits, this novel form of robustness is not specific to the Lorenz equations, and extends generally to chaotic attractors with well‐defined bounded oscillations. Furthermore, the physical implementation is applicable to any medium which can host the requisite non‐linear dynamics, not just electronic circuit metamaterials. As such, our discovery of topologically protected chaos also opens up new possibilities in fields as diverse as neural networks,^[^ ^40^, ^41^ ^]^ chaotic quantum dynamics^[^ ^42^ ^]^ and mechanical metamaterials.^[^ ^43^ ^]^

## 2 Results

### 2.1 Chaotic Chua Oscillators as Unit Cells

To show how topology can protect chaos in a topological lattice, we first design unit cells that host chaotic oscillations. A key ingredient of chaos is non‐integrability, which can be realized in various non‐linear systems of differential equations. A particularly stable candidate is the Chua oscillator,^[^ ^44^ ^]^ and below we review its physical implementation in an electrical circuit.

**Figure 1:**
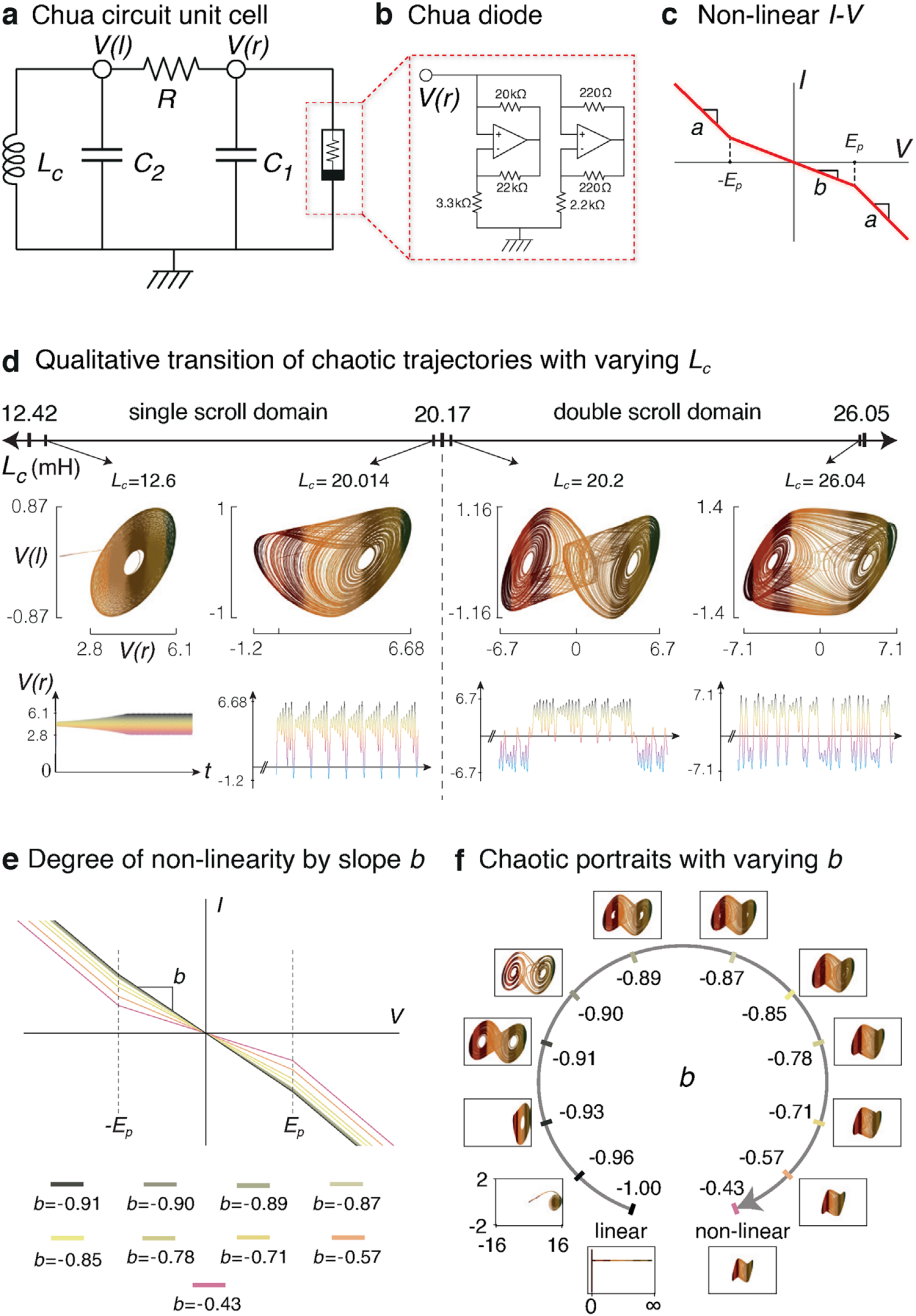
Overview of a Chua circuit and its chaotic dynamics. a) The Chua circuit is a two‐node (*V*(*l*) and *V*(*r*)) circuit comprising four passive components: two capacitors, one inductor, and one resistor, as well as the all‐important active Chua diode. b) The Chua diode provides the requisite non‐linearity for chaotic dynamics, and can be implemented using operational amplifiers in inverting feedback loops. c) The Chua diode's non‐linear *I*–*V* characteristic consists of piece‐wise linear segments, with slopes *b* for the middle segment and *a* for the other segments. d) The oscillations construct chaotic phase portraits in the parametric space of the voltage at the two nodes (obtained from Equation (1)). These portraits are classified based on the total number of equilibrium states, also known as attractors. We illustrate the qualitative transition of the phase portraits from single to double scroll and determine two domains based on the inductance of the inductor (marked on the *L*_*c*_ scale). The lower and upper *L*_*c*_ values of 12.42 mH and 26.05 mH indicate the critical thresholds beyond which the oscillations either quickly attenuate or diverge to infinity, respectively. The bottom row of plots shows the corresponding voltage oscillations at node *V*(*r*), where the Chua diode is attached. Here, *b* = −0.71. e) Representative *I*‐*V* characteristics of the Chua diode with various slopes *b* of the middle segment (of width 2*E*_*p*_). Throughout this study, we set *a* = −1.14 and *E*_*p*_ = 3 V, with variable non‐linearity controlled by *b*. f) The non‐linearity *b* of the Chua diode profoundly controls the type and amplitude of its chaotic scroll, with limit point behavior crossing over to chaotic scrolls as *b* is increased from its linear limit. Here, *L*_*c*_ = 24 mH, and other default parameter values are *C*_1_ = 10 nF, *C*_2_ = 100 nF, and *R* = 1.85 kΩ.

Chua circuits are recognized as the first and among the simplest electrical circuits capable of exhibiting chaos.^[^ ^45^ ^]^ A Chua circuit consists of four passive linear components, namely an inductor (*L*_*c*_), two capacitors (*C*_1_ and *C*_2_), and a regular resistor (*R*), as well as an active non‐linear resistor known as the “Chua diode”, as shown in **Figure** **1**a. While the circuit behaves as a trivial lossy LC oscillator if only passive linear elements are present, an active non‐linear circuit component significantly alters its regular oscillatory behavior. Here, the Chua diode (Figure 1b), has a piece‐wise linear current‐voltage (*I*–*V*) characteristic with non‐constant negative slope, yielding an overall non‐linear negative differential resistance (Figure 1c). In practice, the non‐linear *I*–*V* characteristic of the Chua diode can be achieved by employing appropriately connected operational amplifiers (op‐amps) within a negative feedback loop,^[^ ^46^ ^]^ as detailed in Figure 1b (In our LTspice simulations, we employ LT1351 op‐amps).

Chua circuits form the building blocks of our topological lattice described later, with qualitatively different dynamical behaviors in different parameter regimes. Even though chaotic behavior is inherently stochastic, chaotic trajectories are confined within certain characteristic phase space regimes known as attractors.^[^ ^47^, ^48^ ^]^ Two relevant types of Chua oscillator attractors—the single and the double scroll—are presented in Figure 1d. They arise from the underlying non‐linear Lorenz differential equations^[^ ^1^ ^]^

(1) $$\begin{array}{cc} \frac{dx}{d\tau } & =\alpha (y-x-f_{b}\left(x\right)),\\ \frac{dy}{d\tau } & =x-y+z,\\ \frac{dz}{d\tau } & =-\beta y \end{array}$$

where τ is a dimensionless time variable and *x*(τ), *y*(τ), *z*(τ) represent the dynamical state in 3D space. Of crucial importance is the non‐linear function *f*_*b*_(*x*), which we choose to be the dimensionless non‐linear piece‐wise function

(2) $$f_{b}\left(x\right)=bx+\frac{1}{2}\left(a-b\right)(|x+E_{p}|-|x-E_{p}\left|\right)$$

that replicates the *I*‐*V* characteristic shown in Figure 1c, where *a* and *E*_*p*_ are real constants and *b* is a parameter that controls the extent of non‐linearity (*f*_*b*_(*x*) is linear when *b* = *a*). In general, a wide variety of deterministic and chaotic behavior^[^ ^19^, ^49^ ^]^ exists in various regimes of α, β, and *b*.

Since this work is focused on the experimentally measurable consequences of topologically protected chaotic behavior, rather than the general classification of the Lorenz dynamics, we shall adapt the quantities in Equation (1) to particular electrical circuit component properties that best showcase single and double‐scroll chaotic behavior (see Experimental Section: Relating an Array of Lorenz Equations With Our Chua‐SSH Circuit Lattice). Explicitly, we identify

(3) $$x=\frac{V(r)}{E_{p}},\;y=\frac{V(l)}{E_{p}},\;z=I_{L_{c}}\frac{R}{E_{p}}$$

where *x* and *y* are the dimensionless versions of the right and left node voltages (*V*(*r*) and *V*(*l*), see Figure 1a) normalized by the Chua diode's linear voltage range *E*_*p*_, while *z* is the dimensionless current through the inductor *L*_*c*_ rescaled by *R*/*E*_*p*_. In addition, we define the dimensionless ratios

(4) $$\alpha =\frac{C_{2}}{C_{1}},\;\beta =\frac{R^{2}C_{2}}{L_{c}}$$

Hence, in terms of the physical circuit quantities, Chua circuit dynamics are fully captured by the time dependence of the pair of voltages (*V*(*r*), *V*(*l*)), which will henceforth be plotted as phase portraits (Figure 1d).

Importantly, whether the Chua circuit exhibits regular or chaotic oscillations is regulated by α and β, which can be tuned by varying the relevant circuit components. To reduce the redundancy in this tuning, we will henceforth set the following circuit parameters values as default: *C*_1_ = 10 nF, *C*_2_ = 100 nF, and *R* = 1.85 kΩ, *a* = −1.14, and *E*_*p*_ = 3 V, while keeping *L*_*c*_ and *b* as parameters to be varied, unless otherwise stated.

In Figure 1d, using these default parameters, it is shown that varying the inductance *L*_*c*_ can induce two qualitatively different classes of oscillations in the dynamical phase portraits (*V*(*r*), *V*(*l*)). The voltage oscillations between nodes *V*(*l*) and *V*(*r*) form a single‐scroll chaotic phase portrait when *L*_*c*_ is between 12.42 mH and 20.17 mH. Beyond that, the Chua circuit exhibits double scroll dynamics in between 20.17 mH and 26.05 mH. Around the critical value *L*_*c*_ = 20.17 mH separating these two domains, the phase portrait rapidly deforms and transitions to the other configuration. The oscillations also become unstable at the lower and upper boundaries of these *L*_*c*_ domains (see Section IV in the Supporting Information for detailed analyses of scroll transitions as a function of *L*_*c*_, *C*_1_, and *C*_2_).

Physically, the single and double scroll scenarios are distinguished by the oscillations of *V*(*r*) relative to that of *V*(*l*). While the polarity of the voltage at node *V*(*r*) remains mostly the same in the single scroll cases, i.e., positive, it strongly oscillates between positive and negative voltages in the double scroll cases, as shown in the time evolution of *V*(*r*) in bottom row of Figure 1d.

Besides the inductance *L*_*c*_, the other key tuning parameter *b* controls the non‐linearity of the Chua diode through the slope of the middle segment of its *I*–*V* characteristic. As shown in Figure 1e, the system approaches its linear limit as the parameter *b* is decreased. The corresponding chaotic phase portraits are depicted in Figure 1f for the default parameter combination and *L*_*c*_ = 24 mH. While the exactly linear limit is given by *b* → *a* = −1.14 using our default parameters, to facilitate the analysis of chaotic dynamics, the system is already decidedly linear by *b* = −1. As we approach the linear limit (more negative *b*), the portrait trajectories wander and fluctuate more, until they become non‐chaotic and attenuated beyond *b* = −0.91. These behaviors remain qualitatively similar had we used other physical parameter combinations, as long as we are still in the double scroll domain.

### 2.2 Chaotic Topological SSH Circuit

**Figure 2:**
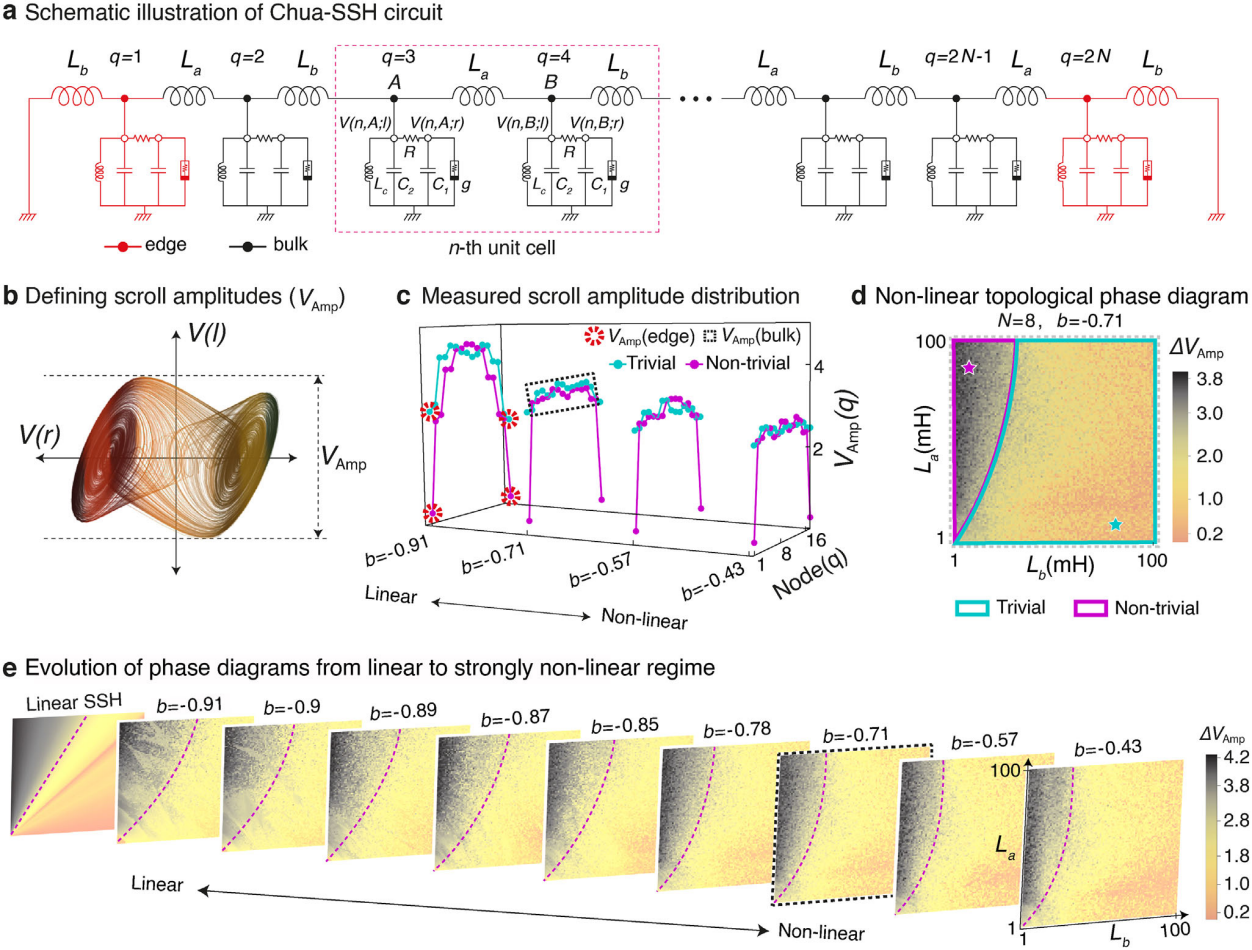
The SSH lattice of Chua oscillators and its topological characterization by extrapolating from its linear limit. a) The Chua‐SSH circuit is constructed by coupling identical Chua circuits at their left nodes (*l*) with alternating intra‐cell and inter‐cell coupling inductances of *L*_*a*_ and *L*_*b*_. *A* and *B* labels the sublattice nodes of each unit cell. We also alternatively label the nodes from *q* = 1 to 2*N*, where *N* is the total number of unit cells. b) Dynamical evolution in one Chua oscillator, as characterized by *V*_Amp_, the peak‐to‐peak amplitude of *V*(*l*) fluctuations. c) By determining the amplitudes of each scroll in the Chua‐SSH lattice, the evolution of the scroll dynamics across the nodes of an *N* = 8 circuit can be plotted for different levels of non‐linearity. Notably, starting from a topologically non‐trivial (magenta with (*L*_*a*_, *L*_*b*_) = (80 mH, 8 mH) corresponding to (δ_*a*_, δ_*b*_) = (4.278, 42.78)) SSH configuration in the linear limit, the edge scrolls are generally significantly suppressed compared to the bulk scrolls. But not so for the topologically‐nontrivial (cyan, (*L*_*a*_, *L*_*b*_) = (8 mH, 80 mH) corresponding to (δ_*a*_, δ_*b*_) = (42.78, 4.278)) cases. Here, δ_*a*_ d) Difference in amplitude *V*_Amp_ (Equation (14)) between the bulk and edge scrolls, with distinct black and yellow regions extrapolated from the topologically non‐trivial and trivial regimes in the linear limit. **e** Evolution of the *V*_Amp_ diagram from the linear to deeply non‐linear regime, showing how the black region continuously evolves from the exact topologically non‐trivial SSH phase in the linear limit (see Experimental Section: Determination of Linear SSH Phase Diagram), manifestly showing how topological edge robustness persists in the non‐linear regime. The inherit topological boundaries (dashed) can be rigorously determined via machine learning (see Experimental Section: Topological Phase Characterizations via Unsupervised Learning). Circuit parameters used in all simulations are *N* = 8, *L*_*c*_ = 24 mH, *C*_1_ = 10 nF, *C*_2_ = 100 nF, *R* = 1.85 kΩ, and *a* = −1.14.

We now show how to construct a topological lattice that admits chaotic oscillations. A paradigmatic 1D topological model is the SSH lattice model, which exhibits robust symmetry‐protected boundary states in its topologically non‐trivial phase.^[^ ^50^, ^51^, ^52^ ^]^ The idea is to replace every site of the SSH lattice with a copy of the Chua circuit, whose dynamics are described by Equation (1). As shown in **Figure** **2**a, the two equivalent sites (*A* and *B*) of each SSH unit cell are thus each grounded with a Chua circuit, and its *l* node of a Chua circuit is connected that of adjacent sites by inductors of either *L*_*a*_ (intra‐cell) or *L*_*b*_ (inter‐cell), depending on the even/oddness of the site position. Hence, each unit cell consists of two identical Chua circuits, such that a *N*‐unit cell Chua‐SSH (cSSH) circuit contains a total of 2*N* Chua circuits.

To elucidate what behavior can be potentially new in topological chaotic circuits, we need to review known behavior in non‐topologically coupled Chua circuits. Firstly, for a pair of coupled Chua circuits (as in the *L*_*b*_ → ∞ case where unit cells are decoupled), the two Chua circuits eventually become synchronized, a phenomenon known as “chaos synchronization,”^[^ ^53^, ^54^ ^]^ which has been extensively studied through various coupling components and combinations.^[^ ^55^, ^56^, ^57^, ^58^, ^59^ ^]^ This synchronization occurs because the information exchange leads to the merging of the attractor points of the two chaotic systems.^[^ ^60^, ^61^, ^62^ ^]^ The time required for synchronization depends on the coupling strength, which essentially determines the amount of shared information.

However, complete synchronization is generally not observed in a 1D topologically trivial array of Chua oscillators.^[^ ^11^, ^20^ ^]^ Instead, they are known to exhibit traveling waves,^[^ ^63^, ^64^ ^]^ homoclinic orbits and solitary waves.^[^ ^21^ ^]^ Yet, complete synchronization is not observed, but only chimera states.^[^ ^65^, ^66^ ^]^

The absence of complete synchronization in a lattice array is due to the chaotic interference between coherent and incoherent groups,^[^ ^67^, ^68^ ^]^ where some portions of the network become synchronized while the rest remain unsynchronized.^[^ ^69^ ^]^ These incoherent groups consistently introduce irregularity to the lattice, amplifying chaotic dynamics across the system. While complete synchronization is already quite rare and difficult to attain—even in networks with global couplings where each oscillator interacts with every other—it becomes unachievable in a 1D lattice array with local (nearest‐neighbor) couplings due to persistent perturbations in the trajectories.^[^ ^70^, ^71^ ^]^ Such interference in general leads to the suppression of periodic behavior in non‐linear chaotic networks.

However, as described later, it turns out that the introduction of topological modes can fundamentally alter the chaotic dynamics. While the aggregate feedback among a lattice of Chua oscillators may intuitively seem to be overall destructive, compromising the oscillation synchronization at the very least, a lattice with protected “topological” states would turn out to have more “robust” chaotic behavior. We would find that chaotic dynamics enjoy some protection by topological non‐trivial‐ness, despite the expectation that non‐linearity and chaos would erode the topology.

To proceed, we define our cSSH circuit (Figure 2a) through its equations of motion. Unlike the uncoupled Chua system described in Equation (1), each variable in Equation (3) is now labeled by its unit cell number *n* and its sublattice node label μ = *A*, *B*, and its notation is generalized to

(5) $$\begin{array}{cc} & x\left(n,\mu \right)=\frac{V(n,\mu ;r)}{E_{p}},\;y\left(n,\mu \right)=\frac{V(n,\mu ;l)}{E_{p}},\\ & \;\;z\left(n,\mu \right)=I_{L_{c}}\left(n,\mu \right)\frac{R(n,\mu )}{E_{p}} \end{array}$$

and for the Chua diode non‐linearity,

(6) $$\begin{array}{ccc} f_{b}\left(x\left(n,\mu \right)\right) & = & bx(n,\mu )\\ & + & \frac{1}{2}\left(a-b\right)(|x\left(n,\mu \right)+E_{p}|-|x\left(n,\mu \right)-E_{p}\left|\right) \end{array}$$

Here, we express the node voltages for the cSSH circuit as *V*(*n*, μ; *l*) and *V*(*n*, μ; *r*), where *l* and *r* label the left and right nodes at position (*n*, μ), respectively (previously, for a single Chua circuit, they were just *V*(*l*) and *V*(*r*)). The dynamical evolution on the *A* sublattice of each Chua circuit is

(7) $$\begin{array}{cc} & \frac{dx(n,A)}{d\tau }=\alpha \left(y\left(n,A\right)-x\left(n,A\right)-f_{b}\left(x\left(n,A\right)\right)\right),\\ & \frac{dy(n,A)}{d\tau }=x\left(n,A\right)-y\left(n,A\right)+z\left(n,A\right)+u\left(n\right)-v\left(n-1\right),\\ & \frac{dz(n,A)}{d\tau }=-\beta y\left(n,A\right) \end{array}$$

while for sublattice *B* they are

(8) $$\begin{array}{cc} & \frac{dx(n,B)}{d\tau }=\alpha \left(y\left(n,B\right)-x\left(n,B\right)-f_{b}\left(x\left(n,B\right)\right)\right),\\ & \frac{dy(n,B)}{d\tau }=x\left(n,B\right)-y\left(n,B\right)+z\left(n,B\right)-u\left(n\right)+v\left(n\right),\\ & \frac{dz(n,B)}{d\tau }=-\beta y\left(n,B\right) \end{array}$$

Notice that Equations (7) and (8) involve two additional dimensionless state variables, i.e., *u* and *v*, representing currents through intra‐cell and inter‐cell couplings *L*_*a*_ and *L*_*b*_ respectively (Figure 2a):

(9) $$u\left(n\right)=I_{L_{a}}\left(n\right)\frac{R(n,\mu )}{E_{p}},\;v\left(n\right)=I_{L_{b}}\left(n\right)\frac{R(n,\mu )}{E_{p}}$$

The temporal evolution of these currents is described by the dimensionless differential voltage as

(10) $$\begin{array}{cc} & \frac{du(n)}{d\tau }=\delta_{a}\left(y\left(n,B\right)-y\left(n,A\right)\right),\\ & \frac{dv(n)}{d\tau }=\delta_{b}\left(y\left(n+1,A\right)-y\left(n,B\right)\right) \end{array}$$

where the dimensionless δ_*a*_, δ_*b*_ depend on inductances *L*_*a*_, *L*_*b*_ via

(11) $$\delta_{a}=\frac{C_{2}R^{2}}{L_{a}},\;\delta_{b}=\frac{C_{2}R^{2}}{L_{b}}$$

Importantly, δ_*a*_ and δ_*b*_ allows for the tuning of the topological phase (alongside the tunable non‐linearity *b*), with other dimensionless coefficients fixed at α = 10 and β = 14.26, as calculated from Equation (4) with fixed *L*_*c*_ = 24 mH and other default circuit parameters. For the coupling inductance range that we shall use in this work, between 1 mH and 100 mH, δ_*a*_ and δ_*b*_ can range from 342.25 to 3.4225.

By combining Equations (7) and (8) with Equation (10), we obtain the equations governing the dynamical evolution of our cSSH circuit. Open boundary conditions are implemented by imposing $\frac{dv(0)}{d\tau }=\delta_{b}y\left(1,A\right)$ and setting *y*(*n* + 1, *A*) = 0. For dynamical simulations, the initial conditions are set as $x\left(n,A\right){|}_{\tau =0}=0.01$, while all other variables are initialized to zero. For the explicit derivation of these equations of motion and the conversions for each dimensionless variable introduced earlier, please refer to Experimental Section: Relating an Array of Lorenz Equations With Our Chua‐SSH Circuit Lattice. Note that for ease of presentation, each node in our simulation results would be labeled with a single numeric index *q* for simplicity. Here *q* ranges from 1 to 2*N*, starting from the left edge, with the corresponding voltages denoted as *V*(*q*; *l*) and *V*(*q*; *r*).

### 2.3 Topological Robustness in the Non‐Linear Regime

Even though topological invariants are rigorously defined only for linear systems,^[^ ^72^, ^73^, ^74^, ^75^, ^76^, ^77^ ^]^ topological signatures in our system interestingly remain robust well into the non‐linear regime. Specifically, we observe that drastic differences between bulk and edge chaotic oscillations persist as our Chua diode is interpolated from the linear to strongly non‐linear regime.

In the linear regime where the slope of the middle segment of the Chua diode's *I*‐*V* characteristic is *b* ≈ *a*, Equations (7), (8), and (10) reduce to the linear relations

(12) $$\begin{array}{cc} & \lambda_{J}V\left(n,A\right)=\frac{1}{L_{a}}V\left(n,B\right)+\frac{1}{L_{b}}V\left(n-1,B\right),\\ & \lambda_{J}V\left(n,B\right)=\frac{1}{L_{a}}V\left(n,A\right)+\frac{1}{L_{b}}V\left(n+1,A\right) \end{array}$$

giving rise to a circuit Laplacian *J* of SSH‐type.^[^ ^23^, ^78^ ^]^ In momentum space (*k*)‐space, it takes the form

(13) $$\begin{array}{c} J\left(k\right)=\left(\begin{array}{cc} \lambda_{J} & -\frac{1}{j\omega L_{a}}-\frac{1}{j\omega L_{b}}e^{-jk}\\ -\frac{1}{j\omega L_{a}}-\frac{1}{j\omega L_{b}}e^{jk} & \lambda_{J} \end{array}\right) \end{array}$$

where $\lambda_{J}=j\omega C_{2}+\frac{1}{R}+\frac{1}{j\omega L_{a}}+\frac{1}{j\omega L_{b}}+\frac{1}{j\omega L_{c}}$ represents the onsite components, *j* is the imaginary unit and ω is the angular frequency. It is well‐known that the SSH model is topologically non‐trivial when *L*_*a*_ > *L*_*b*_, and trivial otherwise;^[^ ^22^, ^79^, ^80^, ^81^, ^82^ ^]^ below, we shall see how this phase boundary is modified in the non‐linear context.

In our Chua circuit lattice, the robustness of the topological edge states is manifested in the dramatically different amplitudes of the voltage oscillations in boundary and bulk sites. As shown in the phase portrait of an isolated Chua oscillator in Figure 2b, oscillations of voltage *V*(*r*) at node *r* of a chosen Chua oscillator site serve to discriminate between scroll types, while *V*(*l*) at node *l* remains relatively constant as non‐linearity is varied (Figure 1f). As such, for the purpose of detecting the persistence of topological signatures as non‐linearity is introduced, we shall focus on *V*_Amp_(*q*), the peak‐to‐peak amplitude of *V*(*q*; *l*). Thus, *V*_Amp_(*q*) omits the Chua node label, as we consistently refer to *V*_Amp_ by the voltage amplitude of the *l* node at the *q*‐th site.

Figure 2c shows the qualitatively different profiles of *V*_Amp_ between topological and non‐topological scenarios, across all the circuit nodes for *N* = 8 unit cells (*q* = 16 nodes). In the linear limit, the cases (*L*_*a*_, *L*_*b*_) = (8 mH, 80 mH) (cyan) and (*L*_*a*_, *L*_*b*_) = (80 mH, 8 mH) (magenta) correspond to the topologically trivial and non‐trivial scenarios, respectively. Note that, the *V*(*l*) amplitude *V*_Amp_ for the trivial case (cyan) is relatively constant across all the sites, apart from slight suppressions at the edge unit cells *n* = 1 and 8 due to edge effects. However, for the topologically non‐trivial case (magenta), the edge *V*_Amp_ is almost 4 volts lower than the *V*_Amp_ within the bulk, due to the presence of topological edge states. Yet, in both topological and non‐topological scenarios, the oscillation amplitudes are very similar across any *V*_Amp_(bulk) (i.e., any bulk node). Hence we take this dramatically suppressed voltage oscillation amplitude at the edge nodes as the signature of topological edge states:

(14) $$\Delta V_{\text{Amp}}=V_{\text{Amp}}\left(\text{bulk}\right)-V_{\text{Amp}}\left(\text{edge}\right)$$

Interestingly, the topological edge suppression of *V*_Amp_ persists even as the Chua diode non‐linearity *b* is tuned into the strongly non‐linear regime, up to *b* = −0.43 (Figure 1e). As apparent in Figure 2c, the edge *V*_Amp_ of the topological scenarios (magenta) remain strongly suppressed even as the circuit becomes significantly non‐linear, such that *V*_Amp_ pertains to the amplitude of the chaotic scrolls *V*(*l*). Even though we have deliberately chosen topological and non‐topological parameters such that either the inter‐ or intra‐cell inductance is much larger, i.e. with *L*_*a*_/*L*_*b*_ = 10, i.e., close to the dimerized limit, our observed topological robust edge behavior contrasts strongly with the synchronized oscillations^[^ ^60^, ^83^, ^84^ ^]^ observed in a fully dimerized cSSH.

Importantly, this persistence of the large edge amplitude suppression Δ*V*_Amp_ for non‐linear chaotic oscillations allows one to define topological phase boundaries even under highly non‐linear conditions. Shown in Figure 2d is a phase diagram capturing the difference between the amplitudes of bulk and edge portraits Δ*V*_Amp_ in the parameter space of *L*_*a*_ and *L*_*b*_, for an illustrative non‐linearity parameter *b* = −0.71. Two distinct regions, namely black and yellow are clearly visible, separated by a rather abrupt gradient in Δ*V*_Amp_. The presence of this rather sharp demarcation parameter regions of high and low Δ*V*_Amp_ provides further support that topological robustness indeed extends into the non‐linear regime, allowing for the continued identification of topological (black) and non‐topological (yellow) regions.

To explicitly identify the two distinct non‐linear parameter space regions with the rigorously defined topological and non‐topological regions in the linearized effective cSSH system, we present the evolution of Δ*V*_Amp_ phase diagrams under various non‐linearities in Figure 2e. By varying the non‐linearity parameter *b* (Figure 1e), we obtain an interpolation from the linear limit (*b* = −0.91, beyond which the chaotic oscillations break down, as elaborated in Experimental Section: Determination of Linear SSH Phase Diagram) to the highly non‐linear regime (up to *b* = −0.43). Indeed, across various degrees of non‐linearity *b*, the phase diagram exhibits a demarcation into two regions (black and yellow) that varies gradually with *b*. Note that the jump in Δ*V*_Amp_ is intrinsically not perfectly sharp even in the linear limit with sharply defined topological boundary *L*_*a*_ = *L*_*b*_, since bulk states also contribute to the voltage dynamics in all cases.

Henceforth, we shall call the black region the “topological” regime. The omnipresence of the distinctive black regions in the phase diagrams of Figure 2e, even in the strongly non‐linear limit (*b* = −0.43), showcases that qualitatively topological edge behavior remains robust for chaotic dynamics. The determination of the non‐linear phase boundaries (purple dashed), which differs from *L*_*a*_ = *L*_*b*_ of the linear limit, can be made rigorous with a machine learning model, as elaborated in Experimental Section: Non‐linear to Linear Phase Boundary Extrapolation. (These phase boundaries remain unchanged for sufficiently long lattices; see Experimental Section: The Effect of Lattice Size on Phase Boundaries, and under structural disorders; see Section III in the Supporting Information.)

### 2.4 Topology Protects Chaos

Contrary to the intuitive expectation that chaotic dynamics would erode the topological properties of a system, we find that, surprisingly, it is however the topology that protects the chaotic dynamics. To investigate the topological robustness of chaotic edge oscillations in an experimentally realistic setting, we performed LTspice circuit simulations of the voltage dynamics driven by an external alternating current (AC) denoted by *I*(*t*) = *I*_0_sin (2π*f*_*r*_ *t*), where *I*_0_ represents the current magnitude and *f*_*r*_ is the AC frequency.

Recalling our default parameter values used in Equations (7), (8), (10) and the coupling inductance values indicated by the stars in Figure 2d (i.e., {*L*_*a*_, *L*_*b*_} = {8 mH, 80 mH}), we find the resonant frequency as |Re(*f*_*r*_)| = 6.42 kHz (see Experimental Section: Determination of Resonant Frequency). For the non‐trivial phase, we set *L*_*a*_ = 80 mH and *L*_*b*_ = 8 mH, with these values switched for the trivial phase. Given that the topological response of topolectrical circuits manifests itself when the signal frequency matches with the resonant frequency,^[^ ^22^, ^23^, ^85^, ^86^, ^87^, ^88^, ^89^ ^]^ we maintain the signal frequency at 6.42 kHz in all simulations.

**Figure 3:**
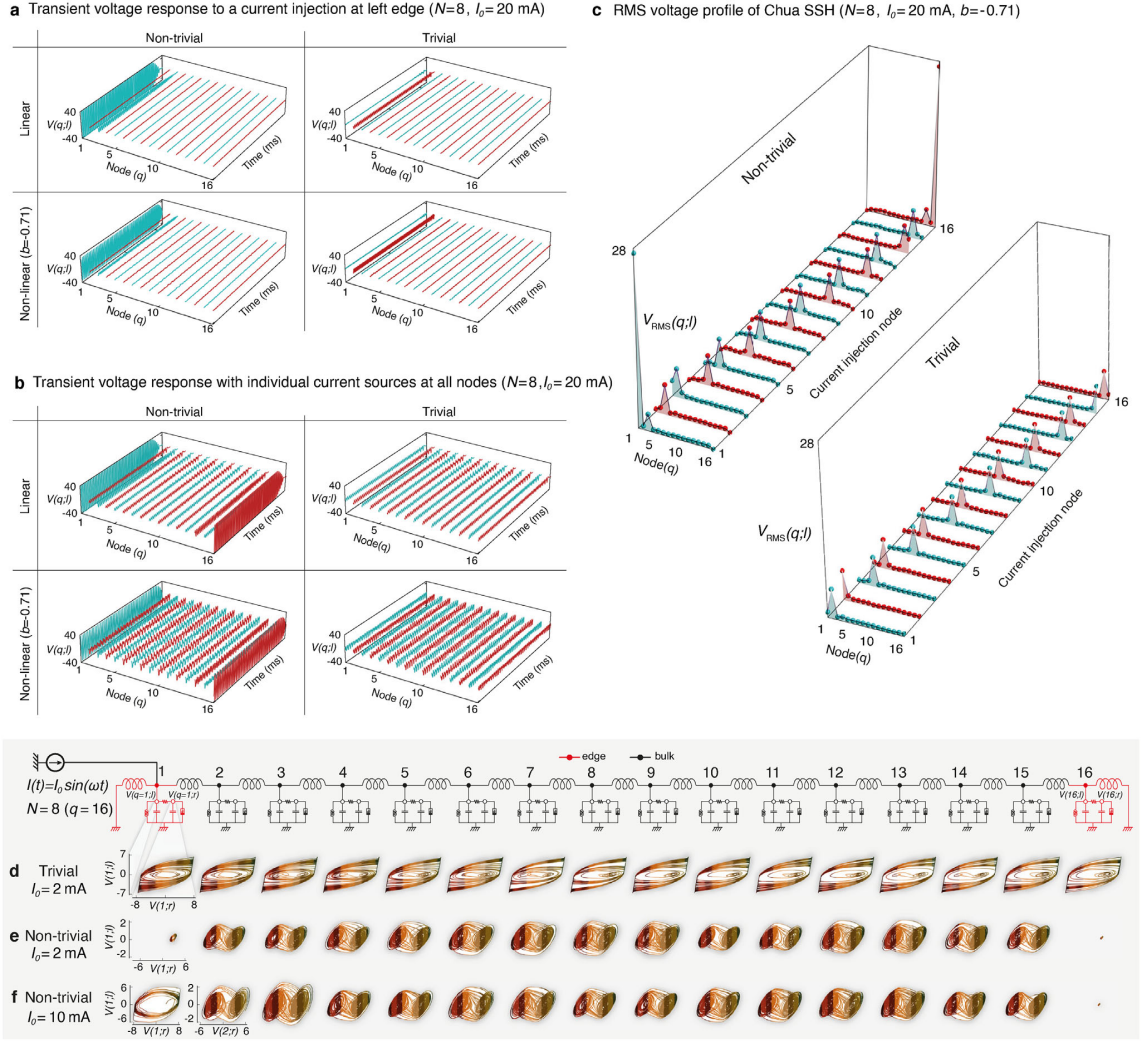
Topological protection of chaotic dynamics under perturbations, analyzed by contrasting topological and non‐linear phases via LTspice simulations. a) Transient voltage response is tested by applying an AC current with a magnitude of *I*_0_ = 20 mA to the left edge (node 1) of an *N* = 8 Chua‐SSH circuit. The linear and non‐linear regimes shows similar profile behaviors, with strong edge oscillations occurring at the left edge in the non‐trivial phase of both regimes. b) Pronounced transient oscillations are observed only at the edges when a current source is applied to each node simultaneously and individually across the circuit. c) RMS voltages are plotted for each node in the topologically non‐trivial and trivial phases, showing clear edge localization in the non‐trivial phase compared to the trivial phase. d) In the topologically trivial case, a relatively small current of 2 mA injected at the left edge (red) transforms chaotic oscillations into limit cycle oscillations shortly after injection, even though each node initially exhibits double‐scroll chaotic behavior without an stimuli. e) By contrast, with topological parameters, chaotic oscillations are robust under the same current injection, i.e., 2 mA, demonstrating the topological protection of chaos in the lattice. f) Further demonstration of the robustness of topological protection, with bulk chaotic portraits remaining intact even under a large current injection of 10 mA. In all cases, for the non‐trivial non‐linear phase (*L*_*a*_, *L*_*b*_, *f*_*r*_, *b*) = (80 mH, 8 mH, 6.42 kHz, −0.71) were used. The values of *L*_*a*_ and *L*_*b*_ are switched for the trivial phase.

The response of our lattice under an external current stimulus provides insights into the robustness of topological dynamics in the presence of circuit non‐linearity, as presented in **Figure** **3**. In Figure 3a,b, we contrast the dynamics between linear and non‐linear scenarios, as well as between topologically trivial and non‐trivial parameter regions. To isolate the protection from topological edge modes, we also compared the effects of injecting the current in three different ways: In Figure 3a, an AC current is injected at the left edge node (node 1) where topological modes reside. The voltage response *V*(*q*; *l*) clearly does not decay with time only at the left node, only when the system has topologically non‐trivial parameters. Most saliently, the oscillations at the left node remain just as robust deep in the non‐linear regime (*b* = −0.71), beyond the scope of usual topological invariants. The prominent edge oscillations, even in the non‐linear regime, indicate the persistence of topological zero modes, which can only be excited by the resonant frequency, set at 6.42 kHz in our system.

Alternatively, in Figure 3b, rather than using a single current source, we attach dedicated multiple current sources *I*_0_ to each node simultaneously and display the voltage oscillations across all nodes to see the global response. As before, the oscillations are consistently much smaller on the bulk nodes (*q* = 2 to 15) at the gapped topological frequency. However, now the oscillations survive at both the left and right edges, further confirming the origin of the robustness from the topological edge modes, given that the current injection occurs democratically at all nodes. Importantly, the voltage dynamics remain qualitatively unchanged as circuit non‐linearity is introduced, like in Figure 3b, a testimony of continued topologically protected robustness.

To further demonstrate that non‐linear oscillations exhibit the same type of edge dependence as topological modes, in Figure 3c, we investigate the circuit response from injecting current one node at a time, for each node, and measuring the resultant root‐mean‐square voltage (*V*_RMS_) at all nodes. Evidently, the edge nodes in the topological case (Figure 3c) exhibit far larger *V*_RMS_ than the bulk nodes, as well as the *V*_RMS_ of all nodes from the topologically trivial case. While the significantly enhanced edge dynamics in the non‐trivial phase and the absence of boundary modes in the trivial phase are well‐known and expected phenomena in the linear regime, the surprising aspect is the persistence of these topological features even in the strongly non‐linear regime.

For a concrete demonstration of how topology protects chaos, we now turn to Figure 3d, which shows the phase portraits *V*(*q*; *l*) vs *V*(*q*; *r*) for every node *q* in the circuit. It is known that chaotic oscillations (which arise in isolated, self‐driven setups) can in general be significantly destroyed by external perturbing drives. This is exemplified in the topologically trivial case in Figure 3d, which shows that the voltages on all nodes exhibit limit cycles instead of chaotic dynamics when an external current of amplitude *I*_0_ = 2 mA is introduced at the left edge (node 1). However, the same current *I*_0_ = 2 mA fails to penetrate the circuit bulk in the topologically non‐trivial case, and the double scrolls saliently survive at all bulk nodes (refer to Figure 3e). Notably, even with a much larger current magnitude, such as *I*_0_ = 10 mA, as shown in Figure 3f, the strong topological edge localization continues to protect the chaotic double scroll oscillations from the perturbative inputs.

Thus, topological protection remarkably enables chaos to persist even under substantial perturbations that would otherwise push the chaotic trajectories to saturate at the op‐amps' output limit (can be even tens of volts) in an isolated Chua circuit. If that were to happen, external perturbations would be amplified by the gain feedback, ultimately leading to limit cycle oscillations. However, this is suppressed by the very robust edge states inherited from linear band structure topology, thereby constituting a topological protective mechanism for the Chua oscillations. (See Supplementary Materials Sections II and III in the Supporting Information for experimentally realizable protected chaos under consideration of disorder, uncertainties, and inevitable loss.)

### 2.5 Phase Boundary Determination From Edge Scroll Attenuation and Effective Impedance

In the following, we describe other observables that provide alternative signs of topological robustness in chaotic lattices.

#### 2.5.1 Edge Scroll Attenuation and Total Number of Double Scrolls

**Figure 4:**
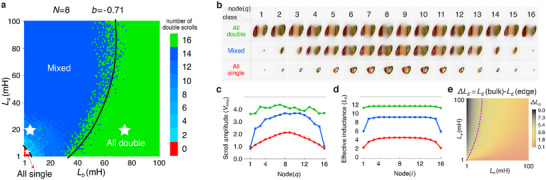
Classification of topological chaos based on edge scrolls attenuation, and its comparison with effective inductance predictions. a) Classification of chaotic lattice dynamics in the parameter space of (*L*_*a*_, *L*_*b*_) based on the number of double and single chaotic scrolls out of 2*N* = 16 Chua oscillators. These scenarios can be categorized into three main classes: i) All‐single (red), where the entire portraits are single scroll, ii) Mixed (shades of blue), where single and double scroll portraits coexist in the circuit, and iii) All‐double (green). The boundaries of these three regimes are defined by a machine learning model involving a wider parameter space (see Experimental Section: Finding Explicit Parametric Equations of Scroll Space Through Machine Learning). b) Scroll profiles of each Chua oscillator, for the three representative cases indicated by the white stars in (a). Saliently, as the edge oscillations are attenuated, some double scrolls are also converted into single scrolls. c) The scroll amplitude profiles for these three examples (green, blue, red) are plotted, with the edge scrolls significantly suppressed in blue but not in green, while the bulk scrolls are suppressed toward the red class. d) The effective inductance *L*_*E*_(*i*) (Equation (15)) profiles corresponding to the same three examples, all which exhibit a qualitative correlation with the scroll amplitudes (also see Section V in the Supporting Information). e) This close qualitative agreement is evident across the entire parameter space, which is mapped by measuring the difference between bulk and edge effective inductances, i.e., Δ*L*_*E*_ = *L*_*E*_(bulk) − *L*_*E*_(edge), similar to Δ*V*_Amp_ diagram. The purple dashed line indicates the approximate phase boundary. Here *b* = −0.71.

Saliently, topological signatures in Chua oscillators lattices also manifest in the scroll pattern of the oscillator dynamics. Since each isolated Chua oscillator exhibits double scroll dynamics by default, the number of double scrolls across all 2*N* oscillators would signify the extent of oscillator independence i.e. lack of correlation. Plotted in **Figure** **4**a is the number of double scrolls in a chain with 2*N* = 16 Chua oscillators, in the parameter space of inductance values *L*_*a*_ and *L*_*b*_. We identify three main classes of behavior: the all‐double scroll class (green region), the all‐single scroll class (red region), as well as the transitional “mixed” class (various shades of blue) with both double and single scrolls coexisting. Interestingly, the double/mixed class boundary in this parameter space plot almost coincides with the topological boundary shown earlier in Figure 2, although the very small all single (red) parameter region represents distinct new behavior.

To understand the suggestive topological origin of the scroll patterns, we provide a visual illustration of the scrolls in Figure 4b. Plotted are the chaotic portraits (defined in Figure 2b) for all the 2*N* = 16 oscillators, for representative parameters in each class (green, blue, red), as indicated by the white stars in Figure 4a. Notably, as we reduce *L*_*b*_ and *L*_*a*_, the double scrolls near the ends of the lattice generally tend to attenuate significantly, such that they become single scrolls. The extent of single scroll penetration increases as *L*_*a*_, *L*_*b*_ → 0 towards the red region, which is also deep inside the topological region found earlier. (For additional examples, see Section VI in the Supporting Information).

In Figure 4c, we quantitatively establish the connection between the classification by number of double scrolls (Figure 4a) with Δ*V*_Amp_ (defined in Equation (14)) which determines the inherited topological regions. Plotted in Figure 4c, is the *V*_Amp_ spatial profile for the abovementioned representative examples in each class (green, blue, red). While edge suppression is less pronounced in the all‐double and all‐single examples compared to the bulk, the edge scroll amplitude is significantly lower than that of the bulk scrolls in the mixed class. Indeed, the blue and red cases with also exhibits significant edge effects, unlike in the purportedly topologically trivial green region where large *L*_*b*_ gives rise to nearly decoupled unit cells.

#### 2.5.2 Node Effective Inductance

Next, we furthermore reveal that the edge sensitivity of the scroll amplitude profiles in Figure 4c can be qualitatively predicted from effective inductance (*L*_*E*_) profile, which is a lumped‐element concept for linear systems. Even though we have a highly non‐linear dynamics, the inspiration is that the network connectivity of the circuit, in particular the elements that give rise to SSH band topology, all stem from linear inductors.

The effective inductance *L*_*E*_(*i*) at a bulk node *i* is given recursively by

(15) $$L_{E}\left(i\right)={\left(\frac{1}{L_{c}}+\frac{1}{L_{E}^{\text{left}}}+\frac{1}{L_{E}^{\text{right}}}\right)}^{-1}$$

where $L_{E}^{\text{left}}$ and $L_{E}^{\text{right}}$ represent the effective lumped inductances on the left and right sides of the *i*th node, respectively. The effective inductance method assumes that chaotic scrolls across different coupling inductances evolve due to the *L*_*E*_ at each node, which results from the cumulative contributions of *L*_*c*_, *L*_*a*_, and *L*_*b*_. (For the detailed derivation and explanation, see Experimental Section: Derivation of Effective Inductance Formulae and Experimental Section: Qualitative Proportionality Between the Effective Inductance and Scroll Amplitude.)

Figure 4d shows the *L*_*E*_(*i*) profiles for each representative example (green, blue, red) from the three regimes in Figure 4a. The effective inductance profile of each example exhibits a very similar trend across the nodes in the chain as the voltage amplitude (Figure 4c). Based on this qualitative correspondence, we proceed to evaluate whether this relationship holds across the entire parameter space. Analogous to the Δ*V*_Amp_ diagram that defines the inherited band topology, in Figure 4e, we plot the effective inductance difference between a bulk node and an edge node, defined as Δ*L*_*E*_ = *L*_*E*_(bulk) − *L*_*E*_(edge). The two distinct regions, which closely resemble the Δ*V*_Amp_ diagrams in Figure 2e, provide strong evidence of a qualitative link between Δ*V*_Amp_ and Δ*L*_*E*_, and also an alternative justification of our non‐linear topological characterization.

Remarkably, the effective inductance approaches can be generalized to multi‐band models. Beyond the dimer SSH, we present an example of a trimer Chua‐SSH circuit, in which the close relationship between effective inductance and scroll amplitudes prominently holds (see Experimental Section: Derivation of Effective Inductance Formulae: Trimer Chua‐SSH Circuit).

## 3 Discussion

The realization and characterization of chaotic topological lattices require careful and new approaches, as existing topological classifications are based on linear systems. Here, we have demonstrated topologically protected chaos in a one‐dimensional topological circuit array of inductively coupled identical chaotic Chua circuits. Counterintuitively, incorporating chaotic dynamics into the periodic structure of the topological SSH circuit results in protected chaos rather than eroding the circuit's topological behavior. In other words, the topological non‐trivial phase of the SSH lattice can protect the bulk dynamical scrolls of the Chua circuit elements from external perturbations, as shown in Figure 3d–f. The topological protection of chaos is significant for preserving chaotic circuits to be potentially used in encryption and secure communications,^[^ ^58^, ^59^, ^62^, ^90^, ^91^ ^]^ where even minor variations in the initial conditions can lead to significant deviations. Moreover, topologically protected chaos might provide pivotal phenomenological stability for potential chaotic processors in analog computing, which enhances machine learning.^[^ ^40^ ^]^ Ensuring these processors are isolated from their environment is essential within a network.

One of the main challenges in such non‐linear topological systems is the inability to evaluate the topological invariants directly. In this work, we utilized an analogous way to evaluate the dynamics of our system inspired by topological bulk‐edge correspondence. Protected topological edge dynamics alters the chaotic phase portraits at each node. Therefore, we topologically characterized our system by measuring the scroll amplitudes for different non‐linearities and coupling strengths, as presented in Figure 2e. The phase diagram obtained from the difference between the bulk and edge scrolls plotted as a function of coupling parameters becomes more pronounced and resembles that of the linear counterpart as non‐linearity is tuned down.

To further investigate the interplay between chaos and topology, we developed an alternative characterization based on the node effective impedance. This approach stems from our observation that the said topology arises from the values of the linear inductors, and that the chaotic dynamics of Chua circuits at each node do not solely rely on individual component values, but rather on the cumulative effective impedance due to other circuit elements at that node. This behavior enabled us to examine the dynamical evolution of both chaotic and topological phenomena in our circuit. Our methodology simplifies the circuit analysis and successfully captures the non‐linear behavior from an approximate linear model. By evaluating the difference between the bulk and edge effective inductance, we observe a similar qualitative phase demarcation as the scroll amplitude evolution (Figure 4e). Furthermore, we provide analytical expressions for the effective inductance calculations. From the scroll amplitude and the effective inductance methods, we construct a comprehensive picture of topologically protected chaos in the circuit.

In addition, our study constitutes an exemplary study of the coexistence of topological phenomena and chaotic dynamics. This work may pave the way for future research into the interplay between topology and multi‐scroll chaotic systems,^[^ ^8^, ^92^ ^]^ where chaotic oscillations extend beyond single or double attractor points. Further studies can examine the effects of using Chua circuit components directly as intra‐cell and inter‐cell couplings, or employing non‐oscillatory couplings such as resistors, diodes, or memristors.^[^ ^93^, ^94^, ^95^ ^]^ Extending our model to include these elements is expected to reveal protected chaos due to the strong persistence of topology, potentially enriching the observations derived from this study.

Finally, we emphasize that our approach is generic and applicable to a variety of platforms, such as photonics,^[^ ^96^ ^]^ acoustics,^[^ ^97^ ^]^ and optical cavities,^[^ ^98^ ^]^ since they are based on mathematical non‐linear differential equations rather than a specific circuit model. The framework and methods presented in this study can further offer new insights into possible topological robustness in diverse chaotic systems such as ecological networks, biological rhythms, and disease dynamics, thus highlighting the relevance of this study to numerous scientific fields.

## 4 Experimental Section

### Determination of Linear SSH Phase Diagram

Here, we describe the process of obtaining the linear SSH amplitude diagram in relation to the voltage Δ*V*_Amp_ diagrams, where distinct regions represent two different behavioral regimes. To compare this qualitative behavior with the topological edge amplitudes across *L*_*a*_ and *L*_*b*_, we examine the SSH circuit in the linear limit, where the non‐linear Chua diode is disregarded as it blocks the AC signal in this regime. Consequently, the SSH Laplacian effectively consists only of the onsite components *L*_*c*_, *C*_2_, and *R* in series with *C*_1_ grounded. The topological phases are characterized by the topological invariant—the winding number *W*—for this circuit, calculated as

(16) $$W=\frac{1}{2\pi j}\int_{-\pi }^{\pi }dk\frac{d}{dk}\log h\left(k\right)$$

where $h\left(k\right)=-\frac{1}{j\omega L_{a}}-\frac{1}{j\omega L_{b}}e^{jk}$ encodes the topological phase information. Our effective Laplacian, given in Equation (13), describes the linear limit of the Chua‐SSH circuit and is non‐trivial with a non‐zero winding number when *L*_*a*_/*L*_*b*_ > 1, and trivial otherwise.

However, while this topological invariant can be calculated for the linear limit Laplacian, it cannot be directly applied once non‐linearity is introduced. Therefore, we instead rely on the consequences of the topological robustness to trace the topological properties of our non‐linear Chua‐SSH circuit. The key idea is to evaluate the difference in bulk and edge state amplitudes as the coupling inductances are varied.

**Figure 5:**
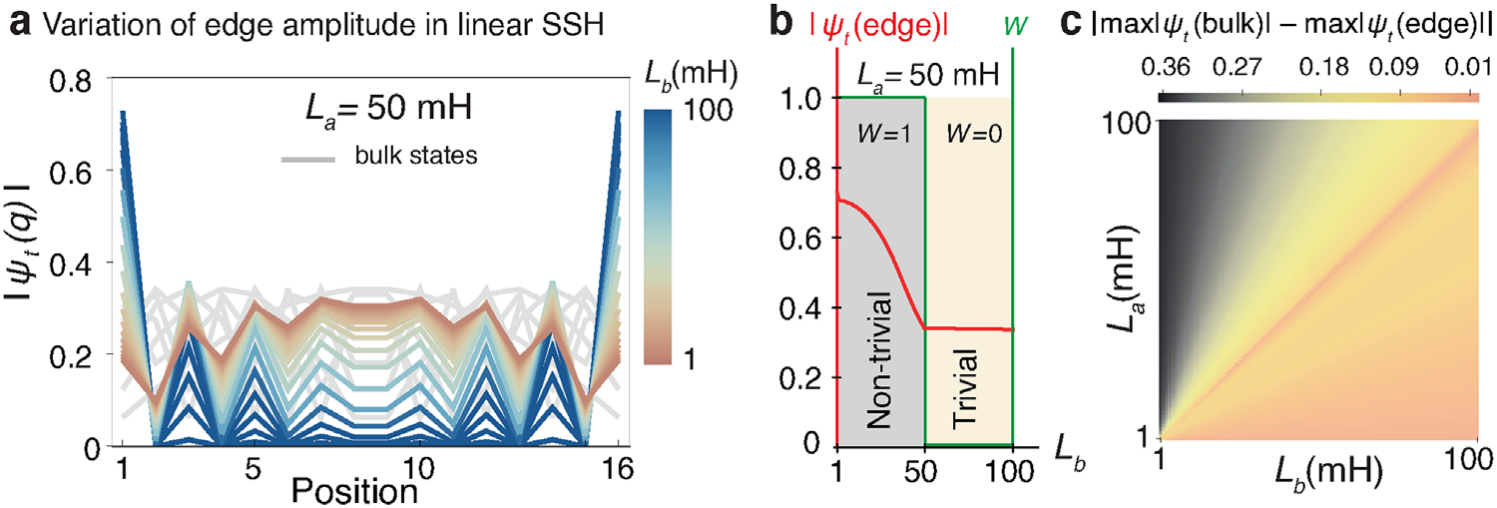
Methodology for determining the linear SSH phase diagram from measurable quantities. a) The SSH circuit in the linear limit exhibits distinct edge states, shown in color as a function of the inter‐cell coupling, with *L*_*a*_ = 50 mH. b) Although the topological invariant changes abruptly at the phase boundary, the edge state amplitudes increase gradually. The red solid line represents the maximum amplitude of the edge states as a function of *L*_*b*_ across both topological phases. c) Analogous to the scroll amplitude diagrams, the phase diagram of the linear SSH can be obtained by analyzing the difference between the edge and bulk eigenstates for each combination of *L*_*a*_ and *L*_*b*_. In the effective circuit Laplacian, the non‐linear Chua diode and the capacitor *C*_1_ are omitted, as the regular resistor strongly dampens their contribution to the effective impedance. Here, |ψ| denotes the eigenmodes of the effective linear SSH Laplacian corresponding to the fully linearized cSSH. The parameters used are (*L*_*c*_, *C*_2_, *R*) = (24 mH, 100 nF, 1.85 kΩ).

In **Figure** **5**a, we show how the edge amplitude changes as the inter‐cell inductance *L*_*b*_ is varied. Despite the quantized nature of the topological invariants, the edge amplitude increases gradually, transitioning from faded‐red to deep‐blue. To highlight this behavior, we plot the maximum edge amplitude in Figure 5b. The parameter regimes highlighted by the gray and yellow‐shaded backgrounds correspond to the non‐trivial and trivial phases, respectively, as defined by Equation (16). While the winding number (green) abruptly drops from *W* = 1 to *W* = 0 at *L*_*a*_ = *L*_*b*_, the maximum edge amplitude (red) begins to decrease gradually as approaching *L*_*b*_ = 50 mH, contrasting with the discrete behavior of the topological invariant.

Similar to our Δ*V*_Amp_ approach, we initially calculate the dynamical state amplitudes using

(17) $$|\psi_{t}\left(q\right)\rangle =e^{-iHt}|\psi_{t_{0}}\left(q\right)\rangle$$

where *H* = *J*/*j*ω is the effective Hamiltonian, and $\psi_{t_{0}}\left(q\right)$ is an initial state. In Figure 5c, we illustrate the difference between the maximum left edge and bulk amplitudes across all *L*_*a*_ and *L*_*b*_ values. The yellow/orange region represents a very small (or negligible) amplitude difference, while the difference increases rapidly, though not suddenly, for smaller *L*_*b*_. This approach, based on directly measurable quantities reflecting topological effects, enables us to evaluate the topological phase diagram without relying on the topological invariant, but instead on measurable values. The strong resemblance between Figure 5c and the Δ*V*_Amp_ diagrams shown in Figure 2e as the system approaches the linear limit provides further evidence of the topological origin of the two regions, black and yellow, in our non‐linear phase diagrams.

### Determination of Resonant Frequency

At the linear limit, the Chua diode can be treated as a regular resistor with a constant value, allowing the calculation of the AC frequency of the circuit without accounting for the complex structure of the diode. This approximation is valid because, with a linear *I*–*V* relationship, the Chua diode no longer contributes to the non‐linear feedback that drives the complex dynamics of the system. In our simulations, at the linear limit, the output voltage of the op‐amps diverged to the saturation value, introducing only a constant DC voltage. Therefore, it becomes feasible to determine the resonant frequency by ignoring the Chua diodes while including all other components in the circuit. The resonant frequency of the circuit is then given by

(18) $$f_{r}=\frac{1+\sqrt{1-4R^{2}\left(C_{1}+C_{2}\right)\left(L_{c}^{-1}+L_{a}^{-1}+L_{b}^{-1}\right)}}{j4\pi R(C_{1}+C_{2})}$$

The resonant frequency is indeed complex, as our Chua‐SSH circuit includes the onsite resistors that are part of the Chua circuits. While the imaginary part represents decay, the real part corresponds to the actual resonant frequency. Using the default parameters, we obtain the resonant frequency as |Re(*f*_*r*_)| = 6.42 kHz. This calculated resonant frequency is further confirmed by the fast Fourier transform, which shows a harmonic peak at this calculated frequency.

### Relating an Array of Lorenz Equations With Our Chua‐SSH Circuit Lattice

**Figure 6:**
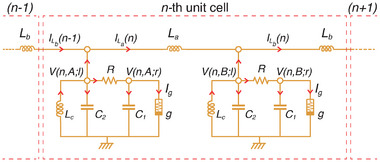
The schematic of a unit cell of our Chua‐SSH circuit. Two sublattice nodes within a unit cell, *A* and *B*, are connected by a coupling inductor with inductance *L*_*a*_, while adjacent unit cells are connected by an inductor with inductance *L*_*b*_. Each unit cell is labeled by *n*, with the nodes on the left and right sides of the conventional resistor *R* denoted as *V*(*n*, μ; *l*) and *V*(*n*, μ; *r*), respectively, where μ = {*A*, *B*}. The red arrows indicate the considered direction of current flow. The non‐linear Chua diodes are labeled as *g*.

The equations of motion for our cSSH circuit are defined by a set of eight dimensionless equations for each unit cell, as illustrated in **Figure** **6**. These equations govern the temporal variation of the current passing through each energy storage component. They are expressed as a function of the unit cell index *n*, as shown in Equations (7), (8), and (10). Our circuit's equation set includes two new dimensionless parameters, δ_*a*_ and δ_*b*_. These parameters represent the energy exchange between the nearest neighboring circuits and cells, respectively. The introduction of these parameters allows us to adjust the circuit's topological phase, owing to their dependence on the intra‐cell and inter‐cell coupling inductances. To derive the system's dimensionless Lorenz equations, which describe our coupled Chua's circuits, we write the currents passing through the components connected to the (*n*, *A*, *l*) and (*n*, *B*, *l*) sublattice nodes as

(19) $$\begin{array}{cc} & I_{C_{2}}\left(n,\mu \right)=I_{L_{c}}\left(n,\mu \right)-I_{R}\left(n,\mu \right)\pm I_{L_{b}}\left(n-1\right)\mp I_{L_{a}}\left(n\right),\\ & I_{C_{1}}\left(n,\mu \right)=I_{R}\left(n,\mu \right)-I_{g}\left(n,\mu \right),\\ & L_{c}\left(n,\mu \right)\frac{dI_{L_{c}}\left(n,\mu \right)}{dt}=-V\left(n,\mu ;l\right) \end{array}$$

where *n* and μ denote the unit cell number and the sublattice nodes *A* and *B*, respectively, that is, μ ∈ *A*, *B*. The signs of the currents $I_{L_{a}}$ and $I_{L_{b}}$ depend on the sublattice node, as will be further detailed in the following derivation. We note that, while this notation is more suitable for deriving the equations of motion, we use *q* in place of (*n*, μ) to indicate position in the figures and discussions. Above, $I_{C_{1}}\left(n,\mu \right)$, $I_{C_{2}}\left(n,\mu \right)$, *I*_*R*_(*n*, μ), and $I_{L_{c}}\left(n,\mu \right)$ represent the currents across the capacitors *C*_1_ and *C*_2_, the regular resistor *R*, and the inductor *L*_*c*_ in each of Chua's circuits, respectively. Since we couple two of Chua's circuits using *L*_*a*_ and the neighboring unit cells with *L*_*b*_, we write the currents across these two additional inductors as follows:

(20) $$\begin{array}{cc} & L_{a}\left(n\right)\frac{dI_{L_{a}}\left(n\right)}{dt}=V\left(n,A;l\right)-V\left(n,B;l\right),\\ & L_{b}\left(n\right)\frac{dI_{L_{b}}\left(n\right)}{dt}=V\left(n,B;l\right)-V\left(n+1,A;l\right) \end{array}$$

where *L*_*a*_ and *L*_*b*_ represent the coupling inductances of the intra‐cell and inter‐cell couplings, respectively, and *V* denotes the voltage at the node indicated by its subscript. We proceed by employing the relationship $I_{i}=C_{i}\frac{dV_{i}}{dt}$ for capacitors. For inductors, the voltage across each inductor is described by the temporal evolution of the current passing through it, as $V_{i}=L_{i}\frac{dI_{i}}{dt}$. To transform the abovementioned equations into Lorenz equations, we introduce the dimensionless variables

(21) $$\begin{array}{cc} & x\left(n,\mu \right)=\frac{V(n,\mu ;r)}{E_{p}},\;y\left(n,\mu \right)=\frac{V(n,\mu ;l)}{E_{p}},\\ & \;\;z\left(n,\mu \right)=I_{L_{c}}\left(n,\mu \right)\frac{R(n,\mu )}{E_{p}},\\ & u\left(n\right)=I_{L_{a}}\left(n\right)\frac{R(n,\mu )}{E_{p}},\;v\left(n\right)=I_{L_{b}}\left(n\right)\frac{R(n,\mu )}{E_{p}} \end{array}$$

where, in addition to the dimensionless variables *x*, *y*, *z*, we introduce *u* and *v*. The term *E*_*p*_ represents the breakpoint voltage, which defines the extent of the slope of the middle segment of the *I*–*V* characteristics of the Chua diode. We further introduce the dimensionless parameters

(22) $$\begin{array}{cc} & \alpha =\frac{C_{2}\left(n,\mu \right)}{C_{1}\left(n,\mu \right)},\;\beta =R{\left(n,\mu \right)}^{2}\frac{C_{2}\left(n,\mu \right)}{L_{c}\left(n,\mu \right)},\\ & \;\;\tau =\frac{t}{R\left(n,\mu \right)C_{2}\left(n,\mu \right)},\\ & \delta_{a}=\frac{C_{2}\left(n,\mu \right)}{L_{a}\left(n\right)}R{\left(n,\mu \right)}^{2},\;\delta_{b}=\frac{C_{2}\left(n,\mu \right)}{L_{b}\left(n\right)}R{\left(n,\mu \right)}^{2} \end{array}$$

### Dimensionless Non‐Linear Function

The non‐linear current‐voltage characteristic of Chua diode is expressed as a piece‐wise function comprising three segments defined by the dimensional *m*_0_ and *m*_1_ slopes, given by

(23) $$\begin{array}{cc} & I_{g}\left(n,\mu \right)=\frac{1}{2}\left(m_{1}-m_{0}\right)\\ & \;\;\;\times \left(|V\left(n,\mu ;r\right)+E_{p}|-|V\left(n,\mu ;r\right)-E_{p}|\right)+m_{0}V\left(n,\mu ;r\right) \end{array}$$

where *E*_*p*_ is the breakpoint voltage of the non‐linear *I*–*V*, which practically defines the extend of the voltage oscillations at the two terminals of the Chua circuits. *V*(*n*, μ; *r*) denotes the voltage at node *V*(*r*) of the μ‐sublattice node (*A* or *B*) of the *n*‐th unitcell. We now introduce the dimensionless parameters for the non‐linear resistor as

(24) $$\begin{array}{cc} & f\left(x\left(n,\mu \right)\right)=I_{g}\left(n,\mu \right)\frac{R(n,\mu )}{E_{p}},\\ & a=m_{1}R\left(n,\mu \right),\;b=m_{0}R\left(n,\mu \right) \end{array}$$

where *I*_*g*_(*n*, μ) represents the non‐linear current across the Chua diode. The slopes of the three segments, defined by *m*_0_ and *m*_1_, are crucial parameters that enable tuning of the dimensionless non‐linearity factors *a* and *b*, expressed in Equations (2) and (6).

Utilizing the dimensionless slope parameters specified in Equation (24), the dimensionless non‐linear function is expressed as

(25) $$\begin{array}{cc} f_{b}\left(x\left(n,\mu \right)\right) & =bx(n,\mu )\\ & \;+\frac{1}{2}\left(a-b\right)(|x\left(n,\mu \right)+E_{p}|-|x\left(n,\mu \right)-E_{p}\left|\right) \end{array}$$

In our simulations, we specifically set *E*_*p*_ = 3 V, and *a* = −1.14, while varying *b* to implement the non‐linear function described above. The subscript *b* in *f*_*b*_ denotes that the non‐linear function depends on *b*.

### Detailed Derivations of Dimensionless Equations

We express the current for the left node (i.e., (*n*, μ; *l*)) of each Chua's circuit in our chaotic SSH circuit as a function of the unit cell *n* and sublattice node μ, as follows

(26) $$I_{C_{2}}\left(n,\mu \right)=I_{L_{c}}\left(n,\mu \right)-I_{R}\left(n,\mu \right)\pm I_{L_{a}}\left(n\right)\mp I_{L_{b}}\left(n-1\right)$$

Here, unlike Equation (19), the sign of $I_{L_{a}}\left(n\right)$ and $I_{L_{b}}\left(n-1\right)$ varies depending on the sublattice node. For example, when μ = *A*, $I_{L_{a}}\left(n\right)$ and $-I_{L_{b}}\left(n-1\right)$, but when μ = *B*, $-I_{L_{a}}\left(n\right)$, and $I_{L_{b}}\left(n-1\right)$. The above equation can be reformulated to express it in terms of the voltage across the capacitors *C*_2_ and the resistors *R* as

(27) $$\begin{array}{cc} C_{2}\left(n,\mu \right)\frac{dV(n,\mu ;l)}{dt}= & I_{L_{c}}\left(n,\mu \right)\pm I_{L_{a}}\left(n\right)\mp I_{L_{b}}\left(n-1\right)\\ & -\frac{1}{R(n,\mu )}\left(V(n,\mu ;l)-V(n,\mu ;r)\right) \end{array}$$

Now, we substitute the dimensionless variables specified in Equations (21) and (22) into the above equation and rewrite it as

(28) $$\begin{array}{cc} \frac{dy(n,\mu )}{d\tau }=\;\; & x(n,\mu )-y(n,\mu )\\ & +\frac{R(n,\mu )}{E_{p}}\left(I_{L_{c}}\left(n,\mu \right)\pm I_{L_{a}}\left(n,\mu \right)\mp I_{L_{b}}\left(n-1\right)\right) \end{array}$$

We then rearrange Equation (21) and derive one of the dimensionless system equations as

(29) $$\begin{array}{c} \frac{dy(n,\mu )}{d\tau }=x\left(n,\mu \right)-y\left(n,\mu \right)+z\left(n,\mu \right)\pm u\left(n\right)\mp v\left(n-1\right) \end{array}$$

Note that the sign of the dimensionless variables *u*(*n*) and *v*(*n* − 1) changes such that $\frac{dy(n,A)}{d\tau }=\cdots +u\left(n\right)-v\left(n-1\right)$ and $\frac{dy(n,B)}{d\tau }=\cdots -u\left(n\right)+v\left(n-1\right)$. Similarly, we express the current for the right node (i.e., (*n*, μ; *r*)) of each Chua's circuit as

(30) $$I_{C_{1}}\left(n,\mu \right)=I_{R}\left(n,\mu \right)-I_{g}\left(n,\mu \right)$$

and

(31) $$\begin{array}{cc} C_{1}\left(n,\mu \right)\frac{dV(n,\mu ;r)}{dt}= & \frac{1}{R(n,\mu )}\left(V(n,\mu ;l)-V(n,\mu ;r)\right)\\ & -I_{g}\left(n,\mu \right) \end{array}$$

We apply the dimensionless variables from Equations (21) and (22), and rewrite the above equation as

(32) $$\frac{C_{1}\left(n,\mu \right)}{C_{2}\left(n,\mu \right)}\frac{dx(n,\mu )}{d\tau }=\left(y(n,\mu )-x(n,\mu )\right)-I_{g}\left(n,\mu \right)\frac{R(n,\mu )}{E_{p}}$$

and finally we have

(33) $$\begin{array}{c} \frac{dx(n,\mu )}{d\tau }=\alpha \left(y(n,\mu )-x(n,\mu )-f(x(n,\mu ))\right) \end{array}$$

To derive the third equation in the Lorenz system, we write the current across the Chua inductor as

(34) $$L_{c}\left(n,\mu \right)\frac{dI_{L_{c}}\left(n,\mu \right)}{dt}=-V\left(n,\mu ;l\right)$$

explicitly write

(35) $$\frac{dI_{L_{c}}\left(n\right)}{d\tau }\frac{L_{c}}{R\left(n,\mu \right)C_{2}\left(n,\mu \right)}=-E_{p}y\left(n,\mu \right)$$

and with β = *R*(*n*, μ)^2^ *C*_2_(*n*, μ)/*L*_*c*_(*n*, μ), we obtain

(36) $$\begin{array}{c} \frac{dz(n,\mu )}{d\tau }=-\beta y\left(n,\mu \right) \end{array}$$

We now derive the dimensionless form of the intra‐cell coupling, denoted as *L*_*a*_(*n*), within a unit cell. The current for the inductors *L*_*a*_(*n*) is expressed as

(37) $$L_{a}\frac{dI_{L_{a}}\left(n\right)}{dt}=V\left(n,B;l\right)-V\left(n,A;l\right)$$

We incorporate the dimensionless parameters from Equations (21) and (22) and reformulate the above equation as

(38) $$\frac{dI_{L_{a}}\left(n\right)}{d\tau }\frac{L_{a}}{R\left(n,\mu \right)C_{2}\left(n,\mu \right)}=E_{p}\left(y(n,B)-y(n,A)\right)$$

and

(39) $$\frac{R(n,\mu )}{E_{p}}\frac{dI_{L_{a}}\left(n\right)}{d\tau }\frac{L_{a}\left(n\right)}{R{\left(n,\mu \right)}^{2}C_{2}\left(n,A\right)}=y\left(n,B\right)-y\left(n,A\right)$$

We now employ the dimensionless new variables *u*(*n*) and δ_*a*_, as explicitly defined in Equation (21) and Equation (22), to further simplify the above equation as

(40) $$\begin{array}{c} \frac{du(n)}{d\tau }=\delta_{a}\left(y(n,B)-y(n,A)\right) \end{array}$$

Similarly, we can now derive the dimensionless form of the inter‐cell coupling, denoted as *L*_*b*_(*n*), within a unit cell. The current for the inductors *L*_*b*_(*n*) is expressed as

(41) $$L_{b}\left(n\right)\frac{dI_{L_{b}}\left(n\right)}{dt}=V\left(n+1,A;l\right)-V\left(n,B;l\right)$$

By following similar steps and utilizing the new dimensionless variables *v*(*n*) and δ_*b*_, we obtain

(42) $$\begin{array}{c} \frac{dv(n)}{d\tau }=\delta_{b}\left(y(n+1,A)-y(n,B)\right) \end{array}$$

We have derived the dimensionless system equations in terms of the unit cell number *n* and the sublattice node μ, which can be either *A* or *B*. While it is possible to treat each component as an individual entity and assign specific values to each, we standardized the components across all unit cells in our cSSH for periodicity. Consequently, we set *L*_*a*_ = *L*_*a*_(*n*), *L*_*b*_ = *L*_*b*_(*n*), *L*_*c*_ = *L*_*c*_(*n*, μ), *C*_1_ = *C*_1_(*n*, μ), *C*_2_ = *C*_2_(*n*, μ), and *R* = *R*(*n*, μ) across the entire lattice.

### Derivation of Effective Inductance Formulae

Here, we derive the effective inductance experienced at any node, and show how it may affect the local topological behavior of its attached chaotic oscillator. In our cSSH circuit, we couple Chua circuits at their *l* nodes via inductors with inductances *L*_*a*_ and *L*_*b*_, forming an SSH circuit with onsite Chua circuits. Coupling identical Chua circuits results in a breakpoint of the identity of Chua circuits because each Chua circuit experiences effectively different inductance at the coupling nodes due to the open boundary conditions. For example, while the total impedance at the *l* nodes is *L*_*c*_ + *L*_*a*_ + *L*_*b*_ + *C*_2_ + *R*, the effective impedance varies depending on the position of the node. Building on this behavior, we assess the circuit's chaotic and topological dynamics using the effective inductance approach, as inductance is a common parameter in both the Chua circuits and the SSH circuit. In the following, we describe how we derive the effective inductance in our circuit by considering an approximate circuit consisting solely of inductive elements.

**Figure 7:**
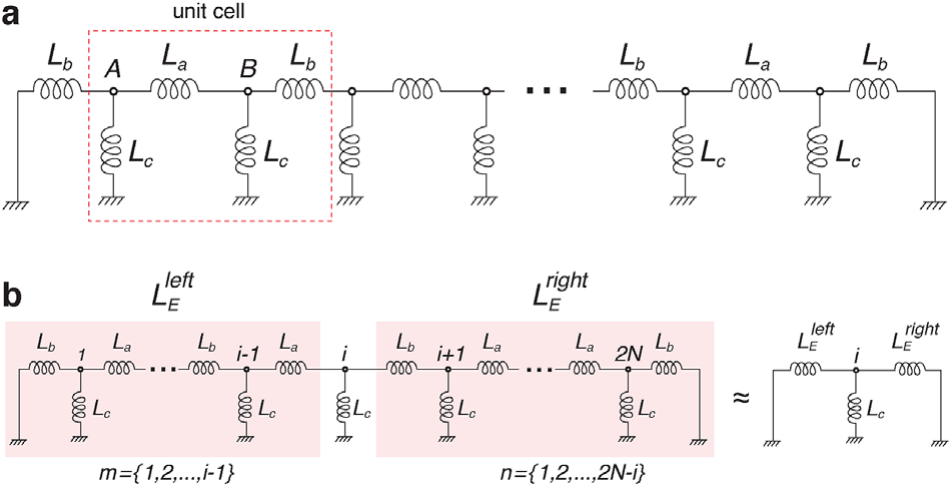
The approximate inductive SSH circuit model with open boundary conditions. a) The chaotic SSH circuit can be approximated to a linear circuit to which only the same type of components are considered. This circuit corresponds to a fully inductive linear SSH circuit with the intra‐cell and inter‐cell inductances *L*_*a*_ and *L*_*b*_ and onsite inductor with inductance *L*_*c*_. b) The effective inductance at each node can be calculated analogous to the lumped‐element method, which considers the impedance of each direction as a single equivalent impedance. This method requires lumping all the elements along each direction and allows us to determine the impedance at a node (inductance for our circuit) by considering each side as a single entity, i.e., $L_{E}^{\text{left}}$ and $L_{E}^{\text{right}}$. The impedance of the lumped elements can be determined starting from the edge elements. *m* and *n* represent the indices of each iteration.

We first derive the effective inductance for the bulk nodes of the circuit depicted in **Figure** **7**a. The effective inductance at a node is not simply the sum of the inductances of the connected inductors (i.e., *L*_*a*_ + *L*_*b*_ + *L*_*c*_); instead, it is determined by the contributions of all the inductors on both sides of the node across the boundary. To calculate the effective node inductance, we lump all the nodes on both sides of the node, as illustrated in Figure 7b. This concept corresponds to the lumped‐element model, where the circuit elements are concentrated at a single node at which the effective inductance can be calculated as

(43) $$L_{E}\left(i\right)={\left(\frac{1}{L_{c}}+\frac{1}{L_{E}^{\text{left}}}+\frac{1}{L_{E}^{\text{right}}}\right)}^{-1}$$

where *L*_*E*_(*i*) represents the effective inductance at the *i*th node, and $L_{E}^{\text{left}}$ and $L_{E}^{\text{right}}$ are the effective lumped inductances of the left and right sides of node *i*, respectively.

**Figure 8:**
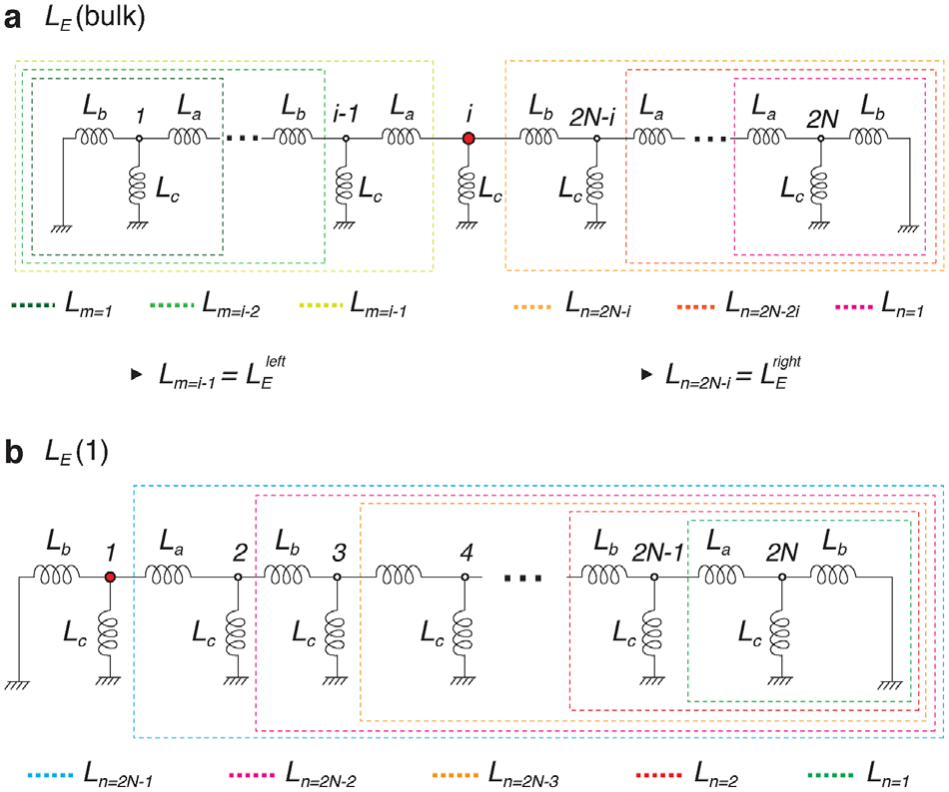
Examples for the derivation of recursive effective inductance for the edge and bulk nodes. a) The effective inductance of a bulk node is obtained by lumping the elements found both sides of the circuit. The effective inductances of the left and right lumps are obtained when $L_{E}^{\text{left}}=L_{m=i-1}$ and $L_{E}^{\text{right}}=L_{n=2N-i}$, respectively. Once $L_{E}^{\text{left}}$ and $L_{E}^{\text{right}}$ are obtained, *L*_*E*_(*i*) is given by Equation (43). b) The effective inductance of an edge node, e.g., *L*_*E*_(1), is obtained by starting to lump the inductors from the right side of the circuit. As we proceed to the opposite edge, we label each summation with the recursion index *n*. *n* = 2*N* − 1 gives us the effective inductance of the right side of the circuit. *N* denotes the total number of unit cells.

Now, to determine $L_{E}^{\text{left}}$ and $L_{E}^{\text{right}}$, we begin from each boundary and sum the inductances towards node *i*. We recursively label the summation of the left and right sides as *L*_*m*_ and *L*_*n*_, where *m* and *n* are indices that range from 1 to *i* − 1 and 1 to 2*N* − *i*, respectively. For example, at both edges (refer to **Figure** **8**a), inductors *L*_*b*_ and *L*_*c*_ are in parallel, and they are in series with *L*_*a*_ in the first iteration. This leads to $L_{m,n=1}={\left(\frac{1}{L_{c}}+\frac{1}{L_{b}}\right)}^{-1}+L_{a}$ for the first iterations. This implies that *L*_*m*, *n* = 1_ effectively reduces the inductance of the three inductors at the edge node to a single equivalent inductor between the edge node and ground. The first iteration corresponds to the dark green and magenta dashed rectangles in Figure 8a. Following the first iteration, the second iteration (i.e., {*m*, *n*} = 2) results in $L_{m=2}={\left(\frac{1}{L_{c}}+\frac{1}{L_{m=1}}\right)}^{-1}+L_{b}$ and $L_{n=2}={\left(\frac{1}{L_{c}}+\frac{1}{L_{n=1}}\right)}^{-1}+L_{b}$. As we progress to node *i*, we obtain the recursive relation

(44) $$\begin{array}{cc} L_{m}= & {\left(\frac{1}{L_{c}}+\frac{1}{L_{m-1}}\right)}^{-1}+L_{a,b},\\ L_{n}= & {\left(\frac{1}{L_{c}}+\frac{1}{L_{n-1}}\right)}^{-1}+L_{a,b} \end{array}$$

where *L*_0_ = *L*_*b*_ and *L*_*a*, *b*_ alternates between *L*_*a*_ and *L*_*b*_ depending on the parity of the indices *m* and *n*. Specifically, for the odd values of *m* and *n*, *L*_*a*, *b*_ assumes the value of *L*_*a*_, while for even values, it takes on the value of *L*_*b*_. The above recursive relations provide us with the effective inductance of each lump when *m* = *i* − 1 and *n* = 2*N* − *i* implying that $L_{m=i-1}=L_{E}^{\text{left}}$ and $L_{n=2N-i}=L_{E}^{\text{right}}$. Once the effective inductances on both sides of the *i*th node are determined, the effective inductance at the *i*th node is given by Equation (43).

In the case of edge node effective inductances, i.e., *L*_*E*_(*i* = 1) (left) and *L*_*E*_(*i* = 2*N*) (right), the recursive indices become *m* = 0 and *n* = 2*N* − 1, or *m* = 2*N* − 1 and *n* = 0, respectively. We illustrate the model for the left edge calculation in Figure 8b. In the edge *L*_*E*_ calculations, we have a single lump covering the remaining nodes, which is the case corresponding to *m* = 0 or *n* = 0.

The effective inductance profile is significantly altered depending on the boundary conditions. To illustrate this, we consider a semi‐infinite circuit where *N* is large enough. We recall Equation (44) and express it for a bulk node as

(45) $$\begin{array}{cc} & L_{E}^{\text{left}}\left(\text{bulk}\right)≊L_{E}^{\text{right}}\left(\text{bulk}\right)=\\ & L_{a,b}+\frac{1}{\frac{1}{L_{c}}+\frac{1}{L_{a,b}+\frac{1}{\frac{1}{L_{c}}+\frac{1}{L_{a,b}+\frac{1}{\frac{1}{L_{c}}+\frac{1}{L_{a,b}+\frac{1}{\ddots }}}}}}}, \end{array}$$

where *L*_*a*, *b*_ takes on value of *L*_*a*_ or *L*_*b*_ depending on the parity of the node *i*. The above equation implies that the effective inductance of bulk nodes is approximately equivalent. This is because $L_{E}^{\text{left}}$ and $L_{E}^{\text{right}}$ have nearly equal weight due to the long‐depth of the fractions.

However, as we approach one of the edges, the depth of one recursion in Equation (45) decreases while the other increases, corresponding to *n* ≪ *m* or *m* ≪ *n*. A decrease (increase) in the recursion depth leads to an overall increase (decrease) in $L_{E}^{\text{left}}$ or $L_{E}^{\text{right}}$. This results in a rapid change in the effective inductance at the nodes near the boundaries. From Equation (43), since the effective inductances appear in the denominator, an increase in the inductance of the lumps leads to a decrease in the overall node inductance as we approach the edges, i.e., *L*_*E*_(*i* → 1) or *L*_*E*_(*i* → 2*N*).

**Figure 9:**
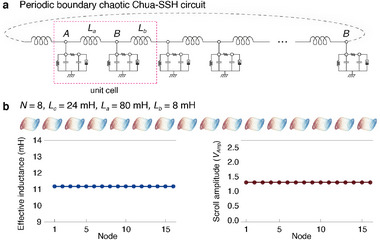
Chua‐SSH circuit under periodic boundary conditions (PBC) and its effective inductance and scroll amplitude profiles. a) The periodic boundary cSSH is achieved by connecting the two edge nodes with *L*_*b*_. The bulk of the circuit under OBC is completely identical to that of the PBC cSSH. b) The demonstration of chaotic portraits, effective inductance, and scroll amplitude profiles exhibits a consistent profile throughout the entire circuit attributed to the implementation of PBC.

Whereas, in the case of periodic boundary conditions (**Figure** **9**a), the effective inductance across the entire circuit is expected to remain uniform. This is because *m* and *n* are approximately equal to *N*, making the contributions of $L_{E}^{\text{left}}$ and $L_{E}^{\text{right}}$ always roughly equal. As a result, *L*_*E*_(*i*) remains constant for all *i*, regardless of its parity. For example, in Figure 9b, we present the effective inductance profile of the periodic Chua‐SSH circuit alongside the scroll amplitude profile, both of which exhibit uniform amplitudes. Remarkably, the scroll amplitudes exhibit qualitatively the same profile as the effective inductance, further supporting the presence of topologically protected chaotic dynamics in the open boundary Chua‐SSH circuit.

### Trimer Chua‐SSH Circuit

**Figure 10:**
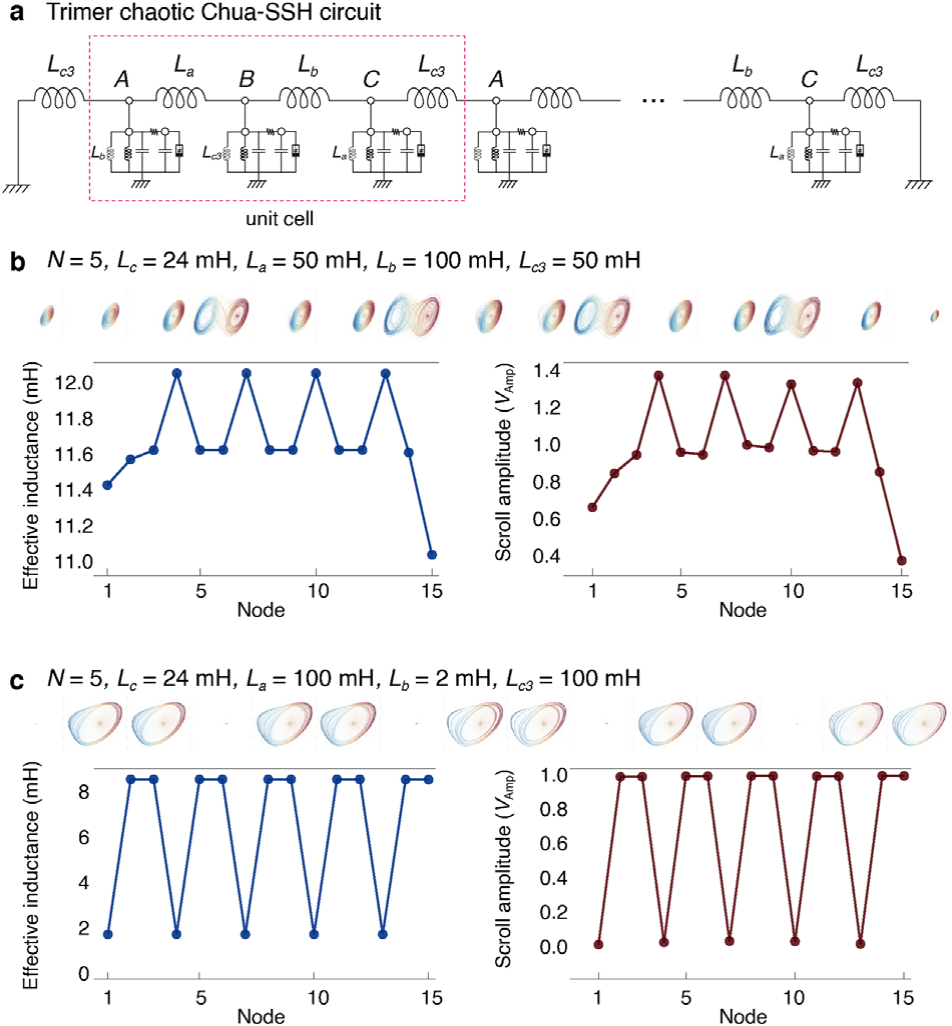
Trimer chaotic Chua‐SSH circuit and examples of its chaotic phase portraits, effective inductance, and scroll amplitude profiles. a) The trimer Chua‐SSH circuit comprises three sublattice nodes—*A*, *B*, and *C*—and three types of inductors with inductances *L*_*a*_, *L*_*b*_, and *L*_*c*3_. Additional inductors are connected between each node and ground to ensure uniform node conductance across the circuit. b,c) Two examples of chaotic phase portraits, effective inductance, and scroll amplitude profiles are presented, demonstrating the effectiveness of the effective inductance approach and its correlation with scroll amplitude in generic circuits. The parameters besides those indicated are the default parameters.

To examine the generality of the qualitative correspondence between the effective inductance and the scroll amplitudes, we present a trimer Chua‐SSH circuit. The inclusion of an additional sublattice node (*C*) in each unit cell requires modifying the circuit's grounding. As shown in **Figure** **10**a, we ground each node with an additional inductor whose inductance corresponds to the coupling inductor that is not connected to the considered node. This arrangement ensures that each node experiences the same conductance. Following this structure, the effective inductance for the trimer chaotic SSH circuit involving the new additional inductance *L*_*c*3_ is given by

(46) $$L_{E}^{\text{TCSSH}}\left(i\right)={\left(\frac{1}{L_{c}}+\frac{1}{L_{a,b,c3}\left(i\right)}+\frac{1}{L_{E}^{\text{left}}}+\frac{1}{L_{E}^{\text{right}}}\right)}^{-1}$$

where

(47) $$L_{a,b,c3}\left(i\right)=\left\{\begin{array}{cc} L_{b}, & \text{if}\;i\;\mathrm{mod}\;3=1,\\ L_{c3}, & \text{if}\;i\;\mathrm{mod}\;3=2,\\ L_{a}, & \text{if}\;i\;\mathrm{mod}\;3=0 \end{array}\right.$$

The effective inductance of the left and right lumped elements, i.e., $L_{E}^{\text{left}}$ and $L_{E}^{\text{right}}$, are determined by

(48) $$\begin{array}{cc} L_{m}= & {\left(\frac{1}{L_{c}}+\frac{1}{L_{b,c3,a}}+\frac{1}{L_{m-1}}\right)}^{-1}+L_{a,b,c3},\\ L_{n}= & {\left(\frac{1}{L_{c}}+\frac{1}{L_{a,c3,b}}+\frac{1}{L_{n-1}}\right)}^{-1}+L_{b,a,c3} \end{array}$$

where each inductance term, such as *L*_*a*, *b*, *c*3_ or *L*_*b*, *c*3, *a*_, represents one of *L*_*a*_, *L*_*b*_, or *L*_*c*3_ depending on mmod3 and nmod3. For example, when mmod3=1, *L*_*b*, *c*3, *a*_ becomes *L*_*b*_; when mmod3=2, it becomes *L*_*c*3_; and when mmod3=0, it becomes *L*_*a*_. This same cyclic logic applies to the additional terms outside the parentheses. The effective inductance of the left and right lumps are achieved when *m* = *i* − 1 and *n* = 2*N* − *i*, implying that $L_{m=i-1}=L_{E}^{\text{left}}$ and $L_{n=2N-i}=L_{E}^{\text{right}}$.

Significantly, as can be seen from the two examples presented in Figures 10b and 10c, the correlation between the effective inductance profile and scroll amplitude is a generic property that can be applied to more complex or higher dimensional non‐linear lattice models to examine the topological properties in non‐linear circuits. Our novel approach offers a valuable approximation for evaluating non‐linear circuits using linear models. We will discuss this by providing analytical solutions of these numerical approaches and demonstrating their general agreement with the scroll amplitude profiles in the following subsections.

### Analytical Effective Inductance Formulae

Utilizing the recursive structure of *L*_*E*_(*i*), it is possible to obtain the analytical expressions for the edge and bulk nodes. The approach relies on assessing each continued fraction representation by expressing the nested dependencies in terms of repeated sequences. The recursive nature gives rise to an infinite continued fraction, which can be simplified by expressing it in terms of two interdependent variables. These variables are defined recursively, with each relying on the other, leading to a nested solution. Solving these coupled equations yields closed‐form expressions for both edge and bulk node inductances. We provide a detailed derivation in Section I in the Supporting Information. Considering a semi‐infinite circuit, we obtain the analytical form of *L*_*E*_(1) as

(49) $$\begin{array}{cc} & L_{E}\left(1\right)=\\ & {\left(\frac{1}{L_{c}}+\frac{1}{L_{b}}+\frac{2}{L_{a}+\sqrt{\frac{\left(L_{a}+2L_{c}\right)\left(2L_{c}\left(L_{a}+L_{b}\right)+L_{a}L_{b}\right)}{L_{b}+2L_{c}}}}\right)}^{-1} \end{array}$$

Similarly, the analytical expression for the second node from the left edge is given by

(50) $$\begin{array}{cc} L_{E}\left(2\right)=\;\; & \left(\frac{1}{L_{c}}+\frac{1}{L_{a}+\frac{1}{\frac{1}{L_{c}}+\frac{1}{L_{b}}}}\right.\\ & {\left.\;+\frac{2}{L_{b}+\sqrt{\frac{\left(L_{b}+2L_{c}\right)\left(2L_{c}\left(L_{a}+L_{b}\right)+L_{a}L_{b}\right)}{L_{a}+2L_{c}}}}\right)}^{-1} \end{array}$$

We provide a closed‐form expression for the third node in Section I in the Supporting Information. The bulk closed‐form effective inductance expression is also obtained as

(51) $$\begin{array}{cc} L_{E}( & \text{bulk})=\\ & \frac{L_{a}L_{c}\left(L_{b}+L_{c}\right)+L_{b}L_{c}^{2}}{\sqrt{\left(L_{a}+2L_{c}\right)\left(L_{b}+2L_{c}\right)\left(2L_{c}\left(L_{a}+L_{b}\right)+L_{a}L_{b}\right)}} \end{array}$$

**Figure 11:**
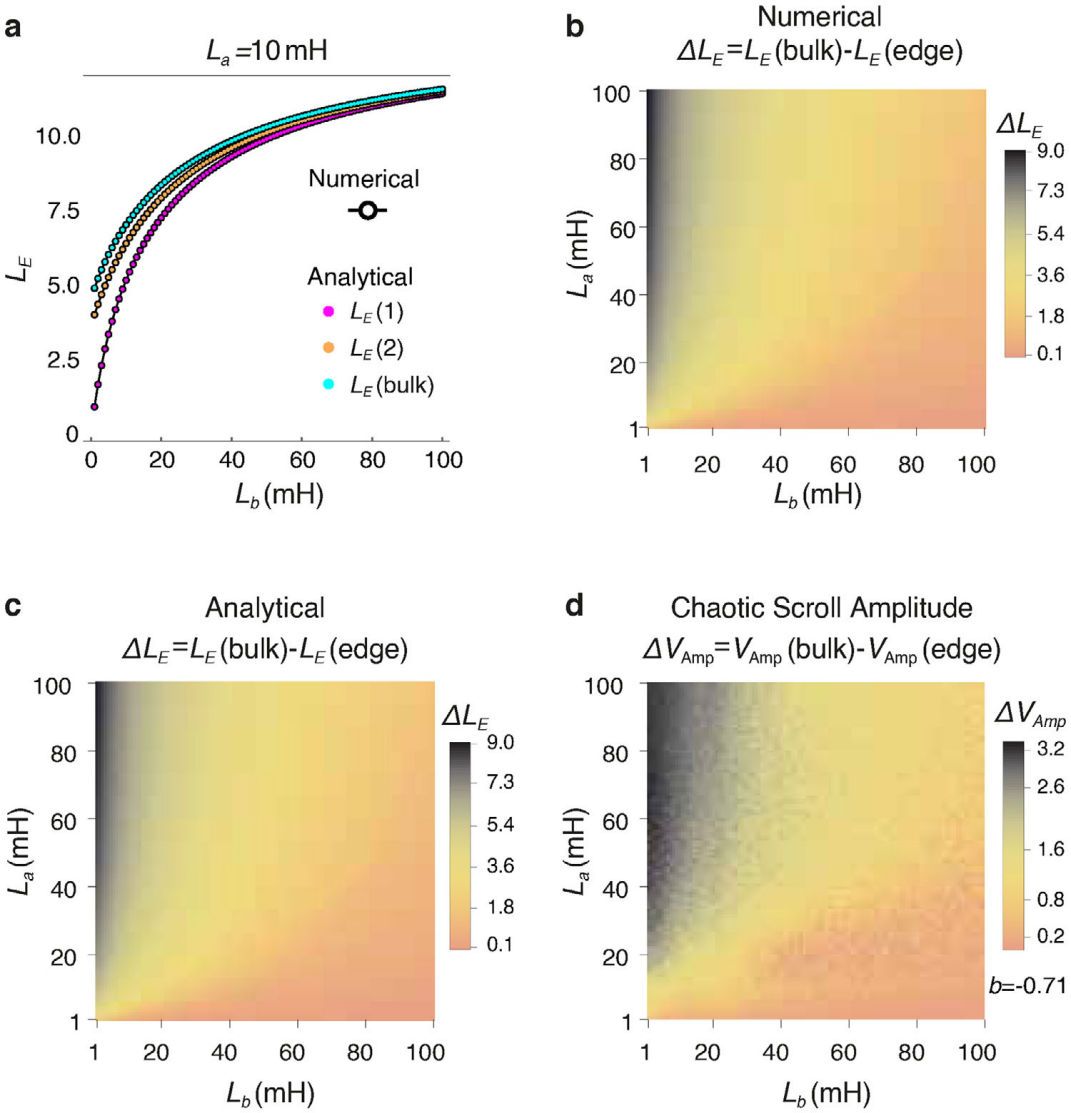
The comparison of the numerical and analytical effective inductance diagrams and the correspondence between the effective inductance and scroll amplitudes. a) The numerical effective inductances of the edge node, second node, and a bulk node, as calculated from Equation (43), show good agreement with the analytical effective inductances of the edge node, second node, and a bulk node calculated from Equations (49), (50), and (51). The parameters used are *L*_*a*_ = 10 mH. b) The numerical effective inductance diagram across *L*_*a*_ and *L*_*b*_ is obtained from Equation (43). The diagram display the *L*_*E*_ difference between bulk and edge nodes. c) The effective inductance diagram is obtained from the analytical closed‐form expressions for the edge and bulk nodes, i.e., Equations (49), and (51). As provided a specific *L*_*a*_ example in (a), both the numerical and analytical effective inductance expressions show good agreement across the entire range of the parameter space. d) The chaotic scroll amplitudes were obtained through numerical simulations of our circuit with *N* = 8. We measured the peak‐to‐peak amplitude of the voltage oscillations at the node *l*s and calculated the difference between the bulk and edge node scroll amplitudes. The resulting density plot of Δ*V*_Amp_ reveals two distinct regions in parallel to the effective inductance diagrams showing their direct correspondence. The noisy appearance of the density plot is attributable to the dynamic characteristics of the chaotic scrolls. For each panel here, we consistently set *L*_*c*_ = 24 mH.

These analytical expressions we derived for the first two edge nodes and a bulk node align well with the numerical effective inductances. In **Figure** **11**a, we present the analytical and numerical *L*_*E*_(1), *L*_*E*_(2), and *L*_*E*_(bulk) results, highlighting significant differences between the bulk and edge inductance for small *L*_*b*_ values. Figure 11b demonstrates this behavior across a wide range of intra‐ and inter‐cell coupling inductances. In Figure 11c, the analytical Δ*L*_*E*_ results align well with the numerical ones across all parameter spaces.

The qualitative correspondence between the effective inductance and the scroll amplitude exhibits very similar behavior across the same parameter space. For example, Δ*V*_amp_ shown in Figure 11d exhibits similar dynamics where the difference between the bulk and edge quantities rapidly increases as the parameters approach the black region, which is the core argument to define the topological phases in our non‐linear lattice. Importantly, this close agreement between the two different quantities allows us to study the non‐linear behavior using the linear effective inductance model.

### Qualitative Proportionality Between the Effective Inductance and Scroll Amplitude

To explain the correlation between the effective inductance and scroll amplitudes, we consider a bulk node in Figure 7b. The current flowing into the bulk node is given by

(52) $$I_{L_{c}}=I_{\text{right}}-I_{\text{left}}$$

where *I*_right_ and *I*_left_ represent the currents flowing through the right and left inductor lumps, respectively. Considering the current flow directions shown in Figure 6, we substitute the current integral expressions into the above relation as

(53) $$\frac{1}{L_{c}}\int V\left(i\right)\;dt=-\frac{1}{L_{E}^{\text{left}}}\int V\left(i\right)\;dt-\frac{1}{L_{E}^{\text{right}}}\int V\left(i\right)\;dt$$

and rearranging this relation, we arrive at

(54) $$\left(\frac{1}{L_{c}}+\frac{1}{L_{E}^{\text{left}}}+\frac{1}{L_{E}^{\text{right}}}\right)\int V\left(i\right)\;dt=0$$

By substituting Equation (43), we obtain

(55) $$\frac{1}{L_{E}\left(i\right)}\int V\left(i\right)\;dt=0$$

The above equation suggests that if *L*_*E*_(*i*) is larger, the node can sustain larger oscillations in voltage (since inductors resist changes in current, causing voltage to rise). In other words, the system allows higher voltage swings when the inductance is high because the inductor slows down current changes. As such the voltage at the node must increase to maintain the same rate of change in current. Conversely, if *L*_*E*_(*i*) is smaller, the voltage needed to drive the same current change is smaller. The integral reflects the scaling of voltage oscillations over time by the effective inductance. For instance, a larger inductance value *L*_*E*_(*i*) allows more energy to be stored, facilitating larger voltage amplitudes at the node, i.e.,

(56) $$V\left(i\right)\propto L_{E}\left(i\right)$$

Taking the derivative of both sides of Equation (55) also shows that the rate of change of current through the inductor is inversely proportional to the inductance. The current flows primarily through the inductors connected to the ground, which offer lower impedance. A low inductance at the edge nodes increases the rate of change of current, compressing the dynamics into a more constrained oscillatory state. Conversely, high inductance at the bulk nodes results in slower voltage evolution. The double‐scroll attractors therefore persist because the state can still alternate between two stable oscillatory regions.

In the case of an external AC current injection, as shown in Figure 3, it is intuitive that the current flows through the ground, as the end nodes are connected to the ground with *L*_*b*_, which is smaller in the non‐trivial phase, corresponding to the black region in Figure 2d.

### The Effect of Lattice Size on Phase Boundaries

**Figure 12:**
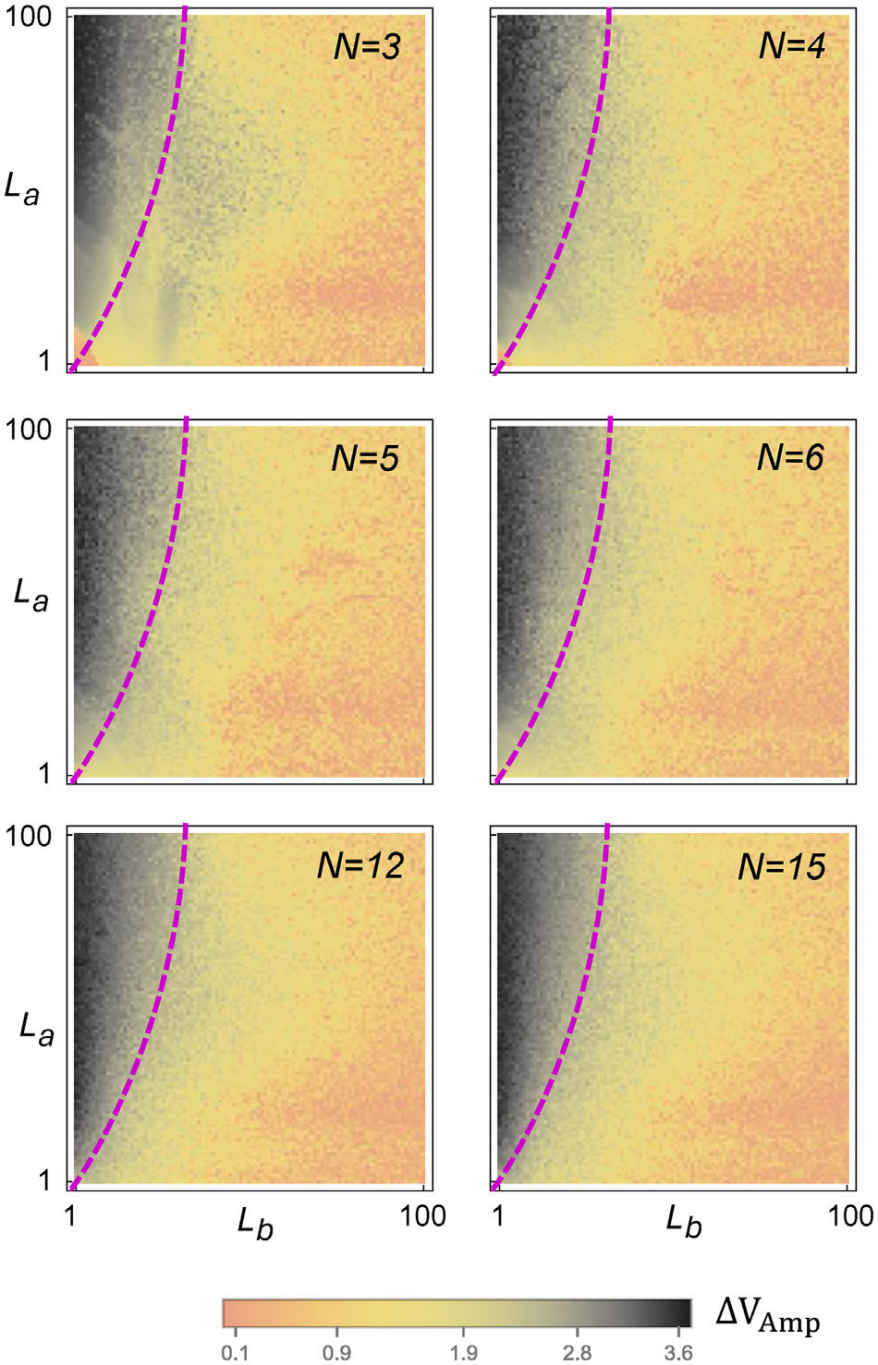
Evaluation of non‐linear phase boundaries under varying lattice sizes. The phase diagrams are obtained from the amplitude difference between bulk and edge scrolls, defined by Equation (14). The magenta dashed lines correspond to the reference phase boundary from Figure 2d (for *N* = 8), facilitating visual comparison. The non‐linear phase boundaries remain qualitatively unchanged for lattice sizes *N* ⩾ 5. Circuit parameters are fixed at (*L*_*c*_, *C*_1_, *C*_2_, *R*, *b*) = (24 mH, 10 nF, 100 nF, 1.85 kΩ, −0.71).

We now discuss the influence of the system size on the non‐linear phase boundaries. In **Figure** **12**, we present non‐linear phase diagrams obtained for various system sizes (*N* = 3, 4, 5, 6, 12, and 15), keeping constant other circuit parameters, including the non‐linearity (*b* = −0.71) and Chua circuit parameters. The magenta dashed lines correspond to the phase boundary from the reference diagram in Figure 2d (for *N* = 8), enabling an easy comparison to observe how the phase boundaries evolve with changing lattice size.

For smaller lattice sizes (*N* = 3, 4), significant deviations from the reference boundary (magenta dashed line) appear, particularly in regions where both *L*_*a*_ and *L*_*b*_ are small. In contrast, for lattice sizes *N* ⩾ 5, the non‐linear phase boundaries remain qualitatively consistent across almost the entire parameter space.

Specifically, for lattices smaller than *N* = 5, the lower‐left regions of the diagrams in Figure 12 predominantly exhibit regular or damped oscillations, indicated by relatively uniform regions compared to chaotic regions, which typically appear noisy. This finding aligns with expectations, as topological phenomena inherently rely on sufficient periodicity within the bulk lattice structure. Hence, a lattice size of at least *N* = 5 unit cells is determined to be sufficient to achieve clearly defined topological protection across the investigated parameter range.

Nevertheless, even for smaller lattices (*N* = 3, 4), when the ratio of *L*_*a*_ to *L*_*b*_ is sufficiently large (e.g., (*L*_*a*_, *L*_*b*_) = (80 mH, 8 mH)), prominent suppression of edge modes in the non‐trivial phase persists. We illustrate this behavior by providing examples of the spatial distribution of chaotic scrolls in Section VI in the Supporting Information.

### Finding Explicit Parametric Equations of Scroll Space Through Machine Learning

We present how we identify the explicit parametric equations for determining the three main scroll classes, i.e., all single, mixed and all double. The black lines in Figure 4a defines the boundaries between the classes. We determine these boundaries by employing linear discriminant analysis (LDA) and support vector machines (SVM). LDA serves as a dimensionality reduction tool that distinguishes classes by maximizing the ratio of between‐class variance to within‐class variance.^[^ ^99^ ^]^ Meanwhile, SVM classifies data by identifying optimal hyperplanes that maximize the margin between classes.^[^ ^100^ ^]^ We utilize the LDA and SVM accuracy results to evaluate linear separability.

**Figure 13:**
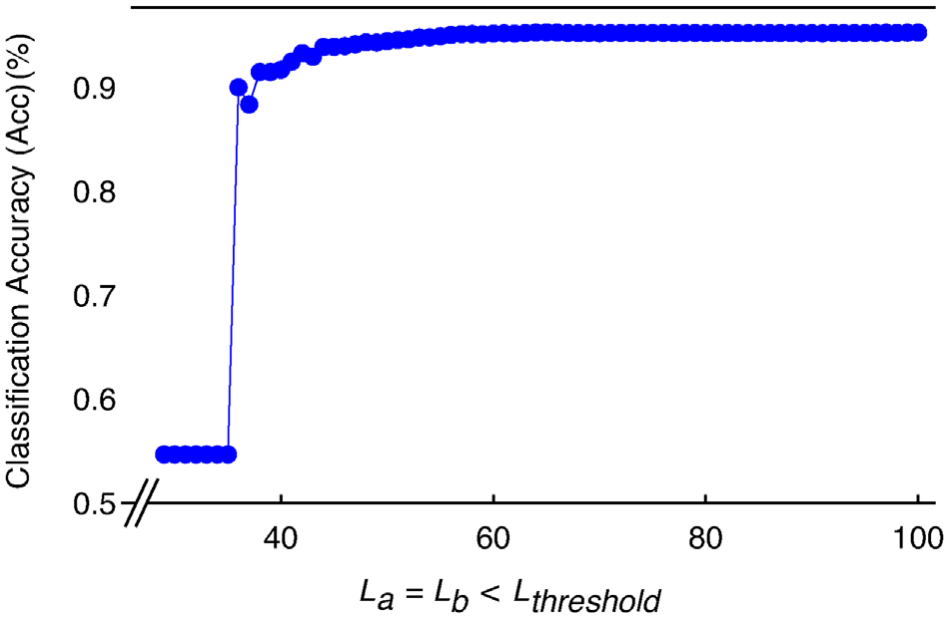
Accuracy evolution over trained parameter region. This figure demonstrates how accuracy across the entire state space changes with respect to a given training space. The training space is defined by a threshold inductance value, i.e., *L*_*a*_ < *L*_threshold_ and *L*_*b*_ < *L*_threshold_, where the *x*‐axis corresponds to *L*_threshold_. The high accuracy (Acc ‐ Equation (57)) remaining beyond *L*_threshold_ = 50 indicates that all information defining the state space can be acquired from the region *L*_*a*_ < 50 and *L*_*b*_ < 50. This suggests that the found equations continue to govern the state space over a much larger extended region.

The state space is likely to be linearly separable if distinct clusters are uncovered by LDA or high accuracy is achieved by SVM. Linear separability suggests the feasibility of using linear equations to determine the boundaries between classes. In our study, we transform from a non‐linear state space (*L*_*a*_, *L*_*b*_) in Figure 4a to a higher‐dimensional state space (*L*_*a*_, *L*_*b*_, *L*_*E*_(bulk), Δ*L*_*E*_). In contrast to the results obtained in the two‐dimensional non‐linear state space, applying LDA and SVM to the newly defined four‐dimensional state space results in separated clusters in the LDA‐reduced space and high classification accuracy (Acc) with SVMs (up to 95% as shown in **Figure** **13**). The accuracy is defined as:

(57) $$\text{Acc}=\frac{1}{M}\sum_{k=1}^{M}I\left(o_{k}={\hat{o}}_{k}\right)$$

Here, *M* is the total number of instances, *o*_*k*_ is the true label of the *k*‐th instance, ${\hat{o}}_{k}$ is the predicted label of the *k*‐th instance, and I(·) is the indicator function. The parametric equations found by SVM define the black lines in Figure 4a, which can be obtained from

(58) $$\left\{\begin{array}{cc} 7.4698L_{E}\left(\mathrm{bulk}\right)-30.8352<\Delta L_{E} & \text{All single},\\ 4.4354L_{E}\left(\mathrm{bulk}\right)-5.7499>L_{b} & \text{Mixed},\\ \text{otherwise} & \text{All double} \end{array}\right.$$

Note that the conditions are evaluated sequentially: if condition 1 is satisfied, the data point belongs to ‘All double’; if condition 1 is not satisfied but condition 2 is, the data point belongs to ‘Mixed’; otherwise, the data point belongs to ‘All single.'

Remarkably, even though the classes can be determined using only data points with *L*_*a*_ < 50 and *L*_*b*_ < 50, yet 95% accuracy is achieved in classifying the extended region *L*_*a*_ < 100 and *L*_*b*_ < 100. By analyzing the accuracy trend across the trained parameter region (Figure 13), it becomes clear that beyond *L*_*a*_ < 50 and *L*_*b*_ < 50, the system provides no additional information, and the accuracy stabilizes. Consequently, these inequalities accurately describe a broader range of unsimulated parameter regions, highlighting their strong generalizability (see Sections VII and VIII in the Supporting Information).

This approach reduces the need for computationally intensive simulations across entire phase spaces and allows for explicit theoretical formulations. Furthermore, this approach can be extended to identify non‐linear phase boundaries using the SVM kernel trick.

### Topological Phase Characterizations Via Unsupervised Learning

Here, we outline how we define the phases in the Δ*V*_Amp_ and Δ*L*_*E*_ diagrams of the main text. The non‐linear topological phase is defined by sudden changes in voltage amplitude difference, i.e., Δ*V*_Amp_, and effective inductance difference, i.e., Δ*L*_*E*_. Beyond studying the qualitative correlation between Δ*V*_Amp_ and Δ*L*_*E*_, we further analyze their phase boundaries to investigate this correlation quantitatively. The *k* means clustering unsupervised learning algorithm and high‐pass Fourier filtering signal processing technique are utilized for this investigation. The *k*‐means algorithm provides the phase transition regions from the statistical distribution of the quantitative difference in the phase diagrams, while Fourier filtering reduces uncertainty near the phase space edges owing to its improved noise resilience.

**Figure 14:**
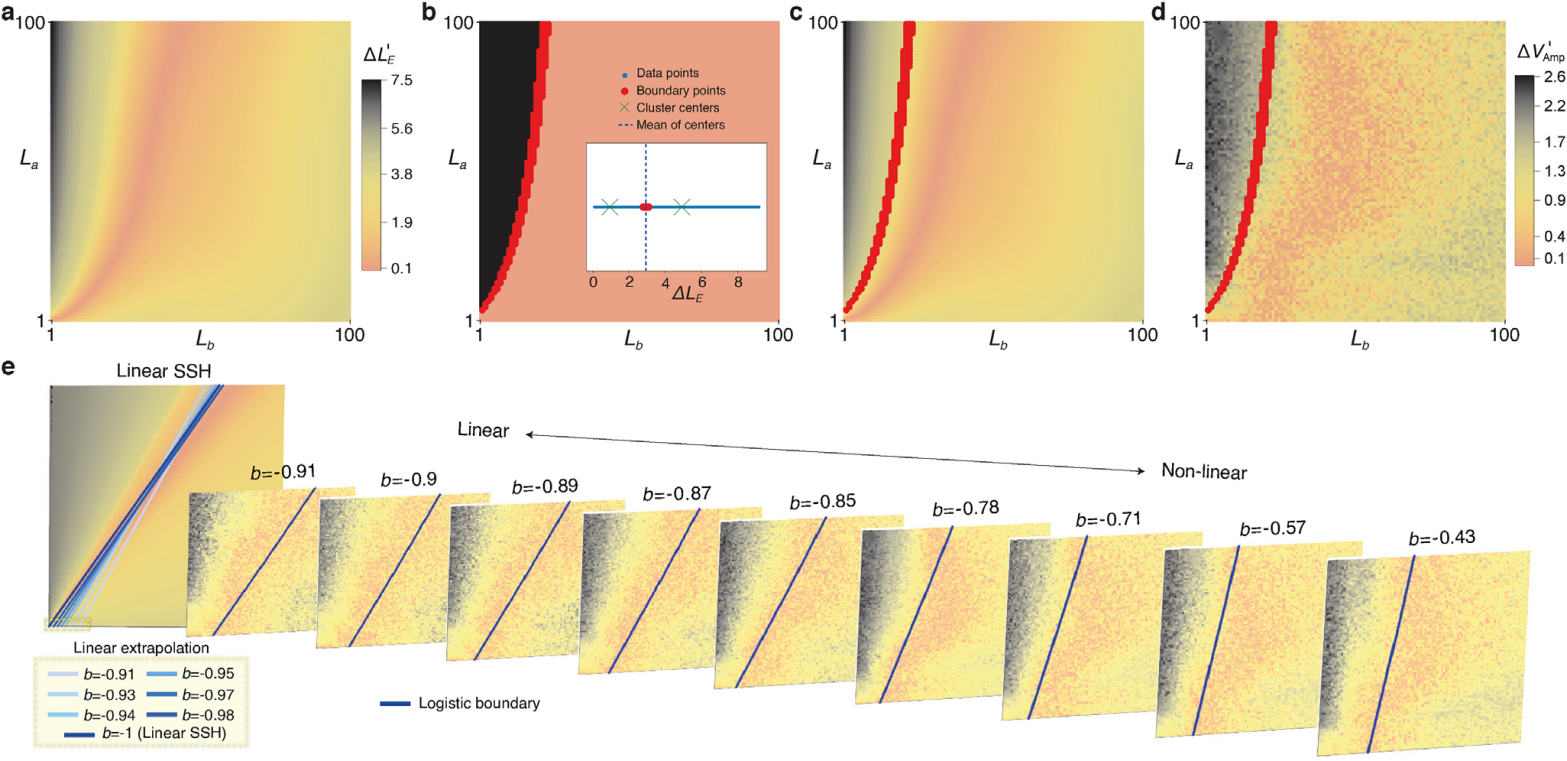
Phase boundary detection and evolution through machine learning. a) High‐pass filtering of Δ*L*_*E*_ around the corresponding k‐means boundary yields $\Delta L_{E}^{\prime }$, allowing us to examine phase transitions in real space with enhanced noise resilience. b) The quantification of the phase boundary is provided by the *k*‐means clustering algorithm, where phase boundaries are defined as equidistant points from the cluster centers. c) Combining these results for Δ*L*_*E*_, the overlap between high‐pass filtering and *k*‐means clustering reinforces confidence in the identified phase boundaries. d) High‐pass filtering of Δ*V*_Amp_ around its k‐means boundary yields $\Delta V_{\text{Amp}}^{\prime }$ where *b* = −0.71, extracting a noise‐resilient phase boundary. Overlaying the *k*‐means boundary of Δ*L*_*E*_ onto this reveals a close alignment, suggesting both phase spaces share the same topological nature. e) We use the *k*‐means algorithm to effectively identify clusters and apply logistic regression to determine the logistic boundaries, shown as blue lines. As chaotic patterns disappear beyond *b* = −0.91, we apply linear regression using the slope and intercept from the previous nine samples’ lines to continue tracking the evolution of the phase boundary. The extrapolated non‐linear boundary directly overlaps with the linear SSH boundary, reinforcing the idea that non‐linear boundaries are reminiscent of the linear topological boundary.

Since our non‐linear phase diagram is defined by the difference between the bulk and edge magnitudes, the high‐frequency components directly define the fast‐changing data points. In this context, Fourier filtering, with its strong noise resilience, emerges as the natural choice. This approach is particularly effective for the Δ*V*_Amp_ phase boundary, given its susceptibility to noise caused by the inherent randomness in its phase space. Utilizing Gaussian high‐pass filtering, we remove slow‐changing points in the state space by selecting high‐frequency components around the identified *k*‐means boundaries. Starting from real space, we apply the Fourier transform to access frequency space, then filter out low frequencies using the following mask function:

(59) $$F=1-e^{-\frac{∥u-u_{0}{∥}^{2}}{2\sigma^{2}}}$$

where **u** represents the frequency space vector corresponding to the real‐space variables (*L*_*a*_, *L*_*b*_), **u**_0_ is the center of the Gaussian function, and σ is its standard deviation. After setting σ = 0.5 and masking frequency space, we apply the inverse Fourier transform to recover real space with the two distinguished phases, as shown for Δ*L*_*E*_ in **Figure** **14**a.

While high‐pass filtering offers noise‐resilient insights by isolating fast‐changing components, *k*‐means clustering provides a statistically rigorous method for identifying clusters and their boundaries in the data. It partitions the data into *k* clusters by iteratively minimizing the sum of squared distances between data points and their corresponding cluster centroids. This corresponds to solving the optimization problem:

(60) $$\min_{\left\{\eta_{k}\right\}}\sum_{k=1}^{k=2}\sum_{q\in \{C_{1},C_{2}\}}{∥q-\eta_{k}∥}^{2}$$

where η_*k*_ is the centroid of cluster $C_{i}$ and *q* denotes a data point. The topologically non‐trivial ($C_{2}$) and trivial regions ($C_{1}$) correspond to different cluster types (black and orange regions in Figure 14b), and the phase boundary is acquired by the equidistant points from the centroids (η_*k*_), as detailed in the inset of Figure 14b.

Using this methodology, we acquire the phase boundary of Δ*L*_*E*_ presented in Figure 14b. We showcase *k*‐means phase boundary of Δ*L*_*E*_ determined in Figure 14b on top of high‐pass filtered diagrams of Δ*L*_*E*_ (Figure 14c) and Δ*V*_Amp_ (Figure 14d). This combined analysis of *k*‐means and high‐pass filtering reveals a remarkable similarity between the phase boundaries of Δ*L*_*E*_ and Δ*V*_Amp_, indicating that both phase spaces are governed by the same topological nature.

### Non‐Linear to Linear Phase Boundary Extrapolation

Chaotic boundary portraits begin to vanish beyond *b* = −0.91 due to the highly linearized *I*‐*V* characteristics. For instance, the partial regular regions emerge as *b* decreases, as shown in Figure 2e, particularly in the small *L*_*a*_ and *L*_*b*_ parameter regime. To estimate whether the phase boundaries determined using the methodology in Figure 14a–d approximate the linear SSH phase boundary beyond *b* = −0.91, we apply cluster identification with the *k*‐means algorithm within the region *b* < −0.91, which enables us to track the evolution of phases effectively. By modeling these boundaries linearly for simplicity and extrapolating their behavior to *b* > −0.91, we find that the extrapolated boundaries in the non‐linear regime closely align with those in the linear regime, demonstrating the continuity in the system's behavior despite the transition.

To acquire the linear boundaries for different *b* settings, we apply logistic regression to the identified *k*‐means clusters. In our setting, logistic regression models the probability of a sample belonging to a phase as:

(61) $$P\left(q\in C_{1}|L_{a},L_{b}\right)=\frac{1}{1+e^{-(\lambda_{J_{0}}+\lambda_{J_{1}}L_{a}+\lambda_{J_{2}}L_{b})}}$$

where $\lambda_{J_{0}}$, $\lambda_{J_{1}}$, and $\lambda_{J_{2}}$ are the model coefficients, and $q\in C_{1}$ corresponds to the sample being part of the topologically trivial cluster. The coefficients $\lambda_{J_{0}}$, $\lambda_{J_{1}}$, and $\lambda_{J_{2}}$ are determined by maximizing the log‐likelihood function, given by:

(62) $$\begin{array}{cc} L(\lambda_{J}) & =\sum_{q\in \{C_{1},C_{2}\}}\left[I\left(q\in C_{1}\right)\log P\left(q\in C_{1}|L_{a},L_{b}\right)\right.\\ & \;\left.+I\left(q\in C_{2}\right)\log P\left(q\in C_{2}|L_{a},L_{b}\right)\right] \end{array}$$

Then the decision boundary is found when the probability is 0.5, which corresponds to:

(63) $$\lambda_{J_{0}}+\lambda_{J_{1}}L_{a}+\lambda_{J_{2}}L_{b}=0$$

This equation represents a logistic boundary separating the classes in the phase space denoted in Figure 14e with blue lines. These lines linearly approximate the visual purple boundaries shown in Figure 2e by using k‐means clusters to demonstrate the direct quantitative evolution from linear to non‐linear.

As chaotic portraits vanish beyond *b* = −0.91, we use linear regression to perform extrapolation based on the previous nine sample's slope (*b* = −0.43 to *b* = −0.91). The extrapolated logistic boundaries are shown in varying blue tones in the first panel of Figure 14e. Additionally, to increase the fidelity of the regression across the fully linear SSH, we used the intercept data ranging from *b* = −0.85 to *b* = −0.91, with an interval of 0.05. See Section IX in the Supporting Information for extraction of extrapolated linear boundaries for the mentioned data points.

This provides a quantitative demonstration of the evolution from the non‐linear to the linear regime, with the overlap of the extrapolated boundary and the SSH linear phase boundary indicating they share the same origin.

## Author Contributions

C.H.L. proposed the idea, initiated, and supervised the project, and provided extensive revisions to the manuscript. H.S. conducted the initial research, developed the theoretical framework, performed numerical and LTspice simulations, and drafted the manuscript. H.A. implemented the machine learning applications and authored the corresponding sections. M.B.A.J. co‐supervised the project. Z.B.S., S.M.R., J.F.K., and M.B.A.J. reviewed and contributed to all aspects of the manuscript. All authors analyzed the results. The manuscript reflects the contributions of all authors.

## Conflict of Interest

The authors declare no conflict of interest.

## Supporting information

Supporting Information

## Data Availability

The datasets and necessary simulation codes generated and/or analyzed during the current study are available in the Zenodo repository at http://doi.org/10.5281/zenodo.15392779.
