# Supplementary material for: Protected Chaos in a Topological Lattice
Source: Adv Sci (Weinh). 2025 May 20;12(28):e03216. doi: 10.1002/advs.202503216 (PMC12302580; doi:10.1002/advs.202503216)
Supplement: Supplementary file 1 — Supporting Information [file ADVS-12-e03216-s001.pdf]

ADVANCED SCIENCE
Open Access

Supporting Information

for *Adv. Sci.*, DOI 10.1002/advs.202503216

Protected Chaos in a Topological Lattice

Haydar Sahin, Hakan Akgün, Zhuo Bin Siu, S. M. Rafi-Ul-Islam, Jian Feng Kong, Mansoor B. A. Jalil and Ching Hua Lee**

Supporting Information for "Protected chaos in a topological lattice"

Haydar Sahin,[1,2,*] Hakan Akgün,[3] Zhuo Bin Siu,[1] S. M. Rafi-Ul-Islam,[1]
Jian Feng Kong,[2,4] Mansoor B. A. Jalil,[1] and Ching Hua Lee[5,†]

[1] *Department of Electrical and Computer Engineering, National University of Singapore, Singapore 117583, Republic of Singapore*
[2] *Institute of High Performance Computing (IHPC), Agency for Science, Technology and Research (A*STAR), Singapore 138632, Republic of Singapore*
[3] *Department of Physics, Bilkent University, Ankara 06800, Türkiye*
[4] *Quantum Innovation Centre (Q.InC), Agency for Science, Technology and Research (A*STAR), 2 Fusionopolis Way, Innovis #08-03, Singapore 138634, Republic of Singapore*
[5] *Department of Physics, National University of Singapore, Singapore 117542, Republic of Singapore*

CONTENTS

I. Detailed derivations of analytical effective inductance formulae 2

II. Monte Carlo-based LTspice simulations of the Chua-SSH circuit under realistic experimental constraints 4

III. The effect of structural disorder 7

IV. Chaotic phase transitions of an isolated Chua circuit 9
- A. Triggering chaos through circuit parameters 9

V. Relating the scroll transitions with effective inductance 10
- A. Scroll transitions in a single isolated Chua circuit 12
- B. Scroll transitions in our Chua-SSH circuit 12

VI. Visual illustrations of scroll profiles across varying lattice sizes 12

VII. Linear Discriminant Analysis (LDA) and Support Vector Machines (SVMs) 13

VIII. Robust machine learning equations under realistic experimental constraints 14

IX. Evolution of Phase Boundaries 17

References 17

* sahinhaydar@u.nus.edu

† phylch@nus.edu.sg

I. DETAILED DERIVATIONS OF ANALYTICAL EFFECTIVE INDUCTANCE FORMULAE

We begin by deriving the closed-form analytical expressions for the edge nodes, i.e., $L_E(i=1)$ and $L_E(i=2N)$, which involve continued fractions due to recursive relations. Given that our circuit exhibits reflectional symmetry about node N, the effective inductance at both edge nodes is equivalent, i.e., $L_E(1)=L_E(2N)$. Therefore, we initially focus on deriving a closed-form expression for the left edge node under the assumption that the right side of the circuit extends semi-infinitely. The effective inductance for the left edge node, where $i=1$, can be ascertained by substituting Equation (44) into Equation (43). This yields

$$L_E(1)=\cfrac{1}{\frac{1}{L_c}+\frac{1}{L_b}+\boxed{\cfrac{1}{L_a+\cfrac{1}{\frac{1}{L_c}+\cfrac{1}{L_b+\cfrac{1}{\frac{1}{L_c}+\cfrac{1}{L_a+\cfrac{1}{\ddots}}}}}}}}. \tag{S1}$$

The above equation realizes Equation (43) of the main text when $m=0$ and $n=\propto$. The red box represents L_E^{right}. Since the red box involves a continued fraction (L_E^{right}) and we are dealing with a nested structure, we can express the two repeated sequences as

$$A=L_a+\left(\frac{1}{L_c}+\frac{1}{B}\right)^{-1}, \tag{S2}$$

where A is defined in terms of B, which means that to solve for A, we express B in a form that eventually leads back to A itself as

$$B=L_b+\left(\frac{1}{L_c}+\frac{1}{A}\right)^{-1}. \tag{S3}$$

The continued fraction represented by A is, in essence, an infinite nested structure that, due to its recursive nature, can be described by a finite formula. Breaking down the recursive dependency typically involves substituting one expression into the other and solving for the variable of interest. Therefore, we solve the above two relations simultaneously and find them as

$$A=\frac{1}{2}\left(L_a+\sqrt{\frac{(L_a+2L_c)\left(2L_c\left(L_a+L_b\right)+L_aL_b\right)}{L_b+2L_c}}\right), \tag{S4}$$

and

$$B=\frac{1}{2}\left(L_b+\sqrt{\frac{(L_b+2L_c)\left(2L_c\left(L_a+L_b\right)+L_aL_b\right)}{L_a+2L_c}}\right). \tag{S5}$$

Now, by rewriting Equation (S1) in terms of A,

$$L_E(1)=\left(\frac{1}{L_c}+\frac{1}{L_b}+\frac{1}{A}\right)^{-1}, \tag{S6}$$

and substituting A into the above equation, we obtain the closed-form expression for the left edge node as

$$L_E(1)=\left(\frac{1}{L_c}+\frac{1}{L_b}+\frac{2}{L_a+\sqrt{\frac{(L_a+2L_c)(2L_c(L_a+L_b)+L_aL_b)}{L_b+2L_c}}}\right)^{-1}. \tag{S7}$$

The above closed-form expression provides an effective inductance for the left edge node, assuming the right side of the circuit is semi-infinite. This expression aligns well with the numerically derived $L_E(1)$, obtained from recursive relations, as can be seen from Figure 11a. It is important to note that $L_E(1) = L_E(2N)$, reflecting the translational symmetry of our circuit. Following a similar approach, we can derive an analytical expression for the second left-edge node (where $i = 2$) by considering the right side of node 2 as a semi-infinite circuit. Applying Equation (43) with $m = 1$ and $n = \infty$ leads to the following numerical recursive relation

$$L_E(2) = \cfrac{1}{\frac{1}{L_c} + \cfrac{1}{L_a + \cfrac{1}{\frac{1}{L_c} + \frac{1}{L_b}}} + \cfrac{1}{L_b + \cfrac{1}{\frac{1}{L_c} + \cfrac{1}{L_a + \cfrac{1}{\frac{1}{L_c} + \cfrac{1}{\ddots}}}}}}. \tag{S8}$$

In the equation above, the right fraction has a continued form due to the semi-infinite approximation, through which we can rewrite it as

$$L_E(2) = \cfrac{1}{\frac{1}{L_c} + \cfrac{1}{L_a + \cfrac{1}{\frac{1}{L_c} + \frac{1}{L_b}}} + \cfrac{1}{B}}. \tag{S9}$$

We can explicitly obtain $L_E(2)$ by substituting B in the above equation as

$$L_E(2) = \left(\frac{1}{L_c} + \frac{1}{L_a + \frac{1}{\frac{1}{L_c} + \frac{1}{L_b}}} + \frac{2}{L_b + \sqrt{\frac{(L_b + 2L_c)(2L_c(L_a + L_b) + L_a L_b)}{L_a + 2L_c}}} \right)^{-1}. \tag{S10}$$

Using the same approximation methods, it is possible to obtain an analytical expression for $L_E(3)$ by initially writing the recursive expression down as

$$L_E(3) = \cfrac{1}{\frac{1}{L_c} + \cfrac{1}{L_b + \frac{1}{\frac{1}{L_c} + \frac{1}{L_a + \frac{1}{\frac{1}{L_c} + \frac{1}{L_b}}}}} + \cfrac{1}{L_a + \cfrac{1}{\frac{1}{L_c} + \cfrac{1}{L_b + \cfrac{1}{\frac{1}{L_c} + \cfrac{1}{\ddots}}}}}}. \tag{S11}$$

We now replace the continued fraction with A and rewrite the above recursive relation as

$$L_E(3) = \cfrac{1}{\frac{1}{L_c} + \cfrac{1}{L_b + \frac{1}{\frac{1}{L_c} + \frac{1}{L_a + \frac{1}{\frac{1}{L_c} + \frac{1}{L_b}}}}} + \cfrac{1}{A}}. \tag{S12}$$

The explicit analytical expression for $L_E(3)$ is obtained when A given in Equation (S4) is substituted in Equation (S12) as

$$L_E(3) = \left(\frac{1}{L_c} + \frac{1}{L_b + \frac{1}{\frac{1}{L_c} + \frac{1}{L_a + \frac{1}{\frac{1}{L_c} + \frac{1}{L_b}}}}} + \frac{2}{L_a + \sqrt{\frac{(L_a + 2L_c)(2L_c(L_a + L_b) + L_a L_b)}{L_b + 2L_c}}} \right)^{-1}. \tag{S13}$$

We have derived the closed-form analytical expressions for the first three edge nodes. Since the effective inductance rapidly converges to the same value as the bulk nodes, this is sufficient to obtain the behaviors of the effective inductance at the boundaries.

We now proceed to derive analytical closed-form expressions for the bulk nodes, employing the same methodology. In contrast to the edge nodes, we are considering a bulk node where both sides are considered to be semi-infinitely large. The recursive relation for such a bulk node is given as

$$L_E(\text{bulk}) = \cfrac{1}{\cfrac{1}{L_c} + \cfrac{1}{L_a + \cfrac{1}{\cfrac{1}{L_c} + \cfrac{1}{L_b + \cfrac{1}{\ddots}}}} + \cfrac{1}{L_b + \cfrac{1}{\cfrac{1}{L_c} + \cfrac{1}{L_a + \cfrac{1}{\ddots}}}}}. \tag{S14}$$

Here, since both sides of the bulk node are semi-infinitely large, there are two continued fractions in the denominator of $L_E(\text{bulk})$. Therefore, we can rewrite the above recursive relation as

$$L_E(\text{bulk}) = \left(\frac{1}{L_c} + \frac{1}{A} + \frac{1}{B}\right)^{-1}. \tag{S15}$$

We obtain the explicit analytical expression for the bulk nodes by substituting A and B into Equation (S15) and simplifying the result as

$$L_E(\text{bulk}) = \frac{L_a L_c (L_b + L_c) + L_b L_c^2}{\sqrt{(L_a + 2L_c)(L_b + 2L_c)(2L_c(L_a + L_b) + L_a L_b)}}. \tag{S16}$$

The closed-form expressions we have derived for the first three edge nodes and bulk nodes (i.e., Equations (S7), (S10), (S13), and (S16)) are sufficient to obtain the effective inductance profile of our circuit.

II. MONTE CARLO-BASED LTSPICE SIMULATIONS OF THE CHUA-SSH CIRCUIT UNDER REALISTIC EXPERIMENTAL CONSTRAINTS

To evaluate the experimental feasibility of protected chaos, we incorporate realistic factors such as component tolerances and parasitic resistances into our LTspice simulations. For this purpose, we use commercially available components selected for their low losses and tight tolerances, as listed in Table I. Among all components, inductors represent the dominant source of loss, and we model them with their specified series resistances. Another important

Component	Value	Manufacturer	Manufacturer No	Tolerance
C_1	10 nF	Murata Electronics	GCM188R72A103KA37D	±10%
C_2	100 nF	Murata Electronics	GCM21BR72A104KA37K	±10%
$L_{a,b}$	8.2 mH	Bourns Inc.	1140-822K-RC	±10%
$L_{a,b}$	82 mH	Abracon LLC	ASPI-0403S-822M	±20%
$L_{c,1}$	22 mH	Delevan	5500R-226J	±5%
$L_{c,2}$	2 mH	Pulse Electronics	APCI00121280202KY0	±10%
R	1.84 kΩ	Yageo	RT0603BRD071K84L	±0.1%
R_1	20 kΩ	Panasonic	ERA-6ARW203V	±0.05%
R_2	22 kΩ	Panasonic	ERA-3ARW223V	±0.05%
R_3	2.2 kΩ	Panasonic	ERA-3ARW222V	±0.05%
R_4	3.3 kΩ	Panasonic	ERA-3VRW3301V	±0.05%
R_5	220 Ω	Panasonic	ERA-3AEB221V	±0.1%
Op-Amp	N.A.	Analog Devices	LT1351CS8	N.A.

TABLE I. **Component specifications used in Monte Carlo-based LTspice simulations.** The table lists the nominal values, manufacturers, part numbers, tolerances of the components used in the LTspice simulations. Here, $L_{c,2}$ is an additional inductor connected in parallel with $L_{c,1}$ to achieve an effective inductance of 24 mH. The selected components are widely available and suitable for experimental implementation. While the nominal tolerances range from 5% to 20% depending on the component type, these can be significantly reduced by preselecting components using standard measurement tools such as an LCR meter or impedance analyzer.

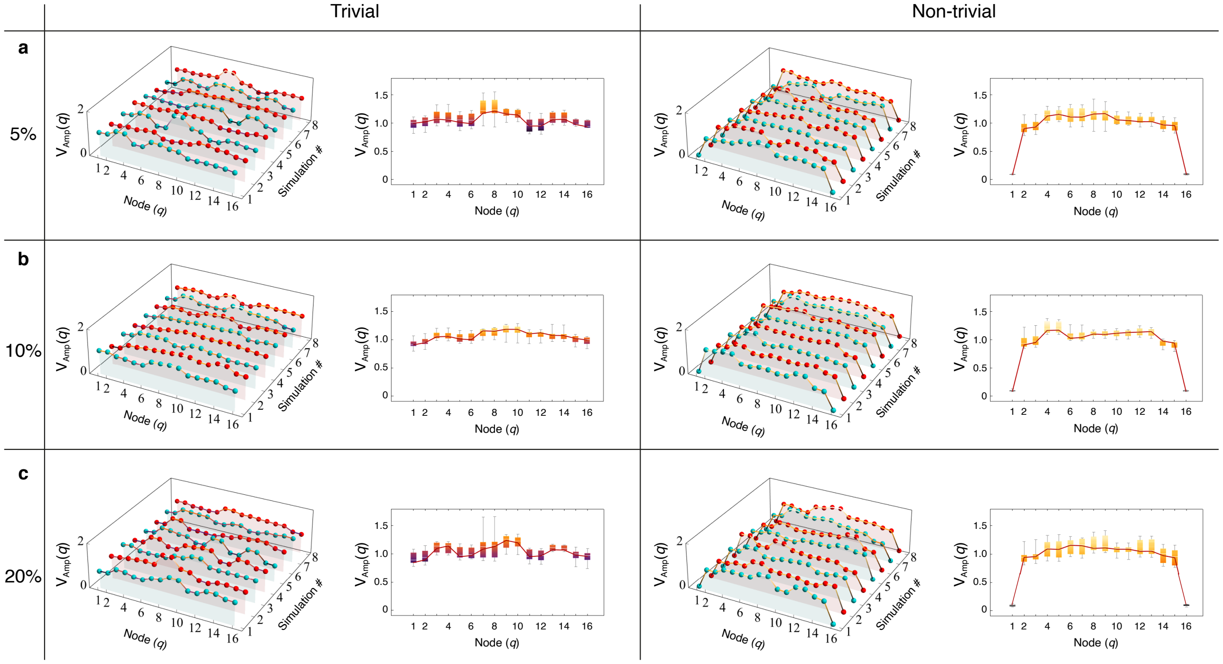

FIG. S1. **Monte Carlo–based LTspice simulations of protected chaos under component tolerances.** Scroll amplitudes $V_{\mathrm{Amp}}(q)$ are shown for trivial and non-trivial phases across 8 Monte Carlo runs for each tolerance level. Left plots in each panel show individual simulation results; right plots display *box-and-whisker* diagrams representing the variation in scroll amplitudes across nodes. **a** 5% tolerance applied to all energy-storage components. **b** 10% tolerance, consistent with typical manufacturer specifications. **c** 20% tolerance as an upper-bound scenario. The protected suppression of edge scrolls in the non-trivial phase remains clearly visible across all cases, indicating strong robustness. Parameters used: $N = 8$, $L_c = 24\,\mathrm{mH}$, $C_1 = 10\,\mathrm{nF}$, $C_2 = 100\,\mathrm{nF}$, $R = 1.85\,\mathrm{k\Omega}$, and $b = -0.71$.

factor in practical implementations is the deviation in component values due to manufacturing tolerances. Although such variations can be reduced by pre-selecting matched components, we systematically explore their impact by performing Monte Carlo tolerance analyses in LTspice across various tolerance levels.

To perform the Monte Carlo worst-case circuit analysis in LTspice, we replace the component values with the command $x \to \mathrm{mc}(x, tol)$, where x is the nominal value of the component and tol denotes the maximum deviation from this nominal value. All other properties from the datasheet are kept unchanged. The function $\mathrm{mc}(x, \mathrm{tol})$ assigns a randomly generated tolerance value to each component for each simulation run. In our analysis, we perform at least eight independent simulations for each case to ensure adequate sampling of possible variations within the specified tolerance window.

The simulations are based on the parameter set extensively studied in the main text: $N = 8$, $L_c = 24\,\mathrm{mH}$, $C_1 = 10\,\mathrm{nF}$, $C_2 = 100\,\mathrm{nF}$, $R = 1.85\,\mathrm{k\Omega}$, and $b = -0.71$. For the trivial and topological phases, we use $(L_a, L_b) = (8.2\,\mathrm{mH}, 82\,\mathrm{mH})$ and $(L_a, L_b) = (82\,\mathrm{mH}, 8.2\,\mathrm{mH})$, respectively. In these simulations, we apply a fixed 5% tolerance to C_1 and C_2, and vary the tolerance for the inductors L_a, L_b, and L_c using 5%, 10%, and 20% deviation windows. The resistors are not perturbed in these runs, as their specified tolerances are already below 0.1%, as listed in Table I. Wire resistances are also included by setting the series resistances to the inductors as follows: $25\,\mathrm{m\Omega}$ for the $24\,\mathrm{mH}$ inductor, $75\,\mathrm{m\Omega}$ for the $8.2\,\mathrm{mH}$ inductor, and $150\,\mathrm{m\Omega}$ for the $82\,\mathrm{mH}$ inductor. This setting effectively reflects an experimental circuit setup.

We first consider a scenario in which all energy-storage components—C_1, C_2, L_a, L_b, and L_c—are assigned a maximum deviation of 5%. In practice, such deviations can be minimized by preselecting components using measurement tools such as an LCR meter or an impedance analyzer. Each simulation is run for a sufficiently long duration (a few hundred milliseconds), and we record the voltage oscillations at the left nodes of each Chua circuit, denoted by $V(q; l)$, which determine the scroll amplitudes. In experiments, this voltage amplitude can be directly accessed by probing the left node of a Chua circuit with an oscilloscope, where the peak-to-peak amplitude can be extracted using standard oscilloscope functions.

We repeat the simulations eight times and plot the scroll amplitudes, $V_{\mathrm{Amp}}(q)$, at each node q for all runs. The results for both the trivial and non-trivial phases are shown in the left column of each panel in Figure S1. In the corresponding right column of each panel, we present the node voltage amplitude distributions using *box-and-whisker*

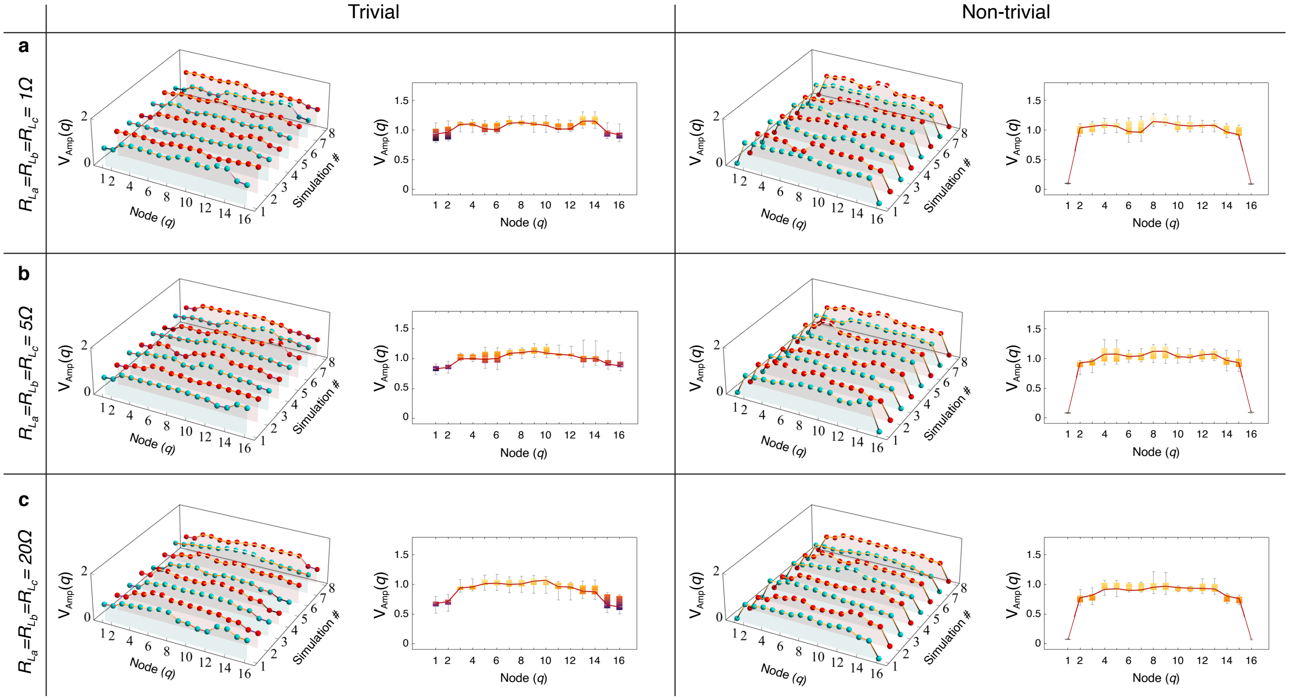

FIG. S2. **Monte Carlo–based LTspice simulations of protected chaos under increasing losses.** Scroll amplitudes $V_{\mathrm{Amp}}(q)$ are shown for simulations including fixed tolerances and increasing series resistance in all inductors. Left plots show individual simulation results; right plots show node-wise box-and-whisker distributions. **a** Series resistance set to $1\,\Omega$. **b** Increased to $5\,\Omega$. **c** Increased further to $20\,\Omega$. The qualitative features of protected chaos, including edge-mode suppression in the non-trivial phase, persist across all resistance values. Parameters used: $C_1 \pm 2\%$, $C_2 \pm 2\%$, L_a, L_b, $L_c \pm 5\%$, and other values same as in Figure S1.

plots. Each *whisker* indicates the minimum and maximum $V_{\mathrm{Amp}}(q)$ values observed across all eight simulations, while each *box* represents the median value. For visual clarity, we also include a red curve connecting the medians across nodes to highlight the overall scroll amplitude distribution trend.

In Figure S1, we present the simulation results of the Chua-SSH circuit under 5%, 10%, and 20% tolerance levels. Figure S1a corresponds to simulations with 5% deviations, representing experiments with pre-selected components. Figure S1b shows results for 10% tolerances, which reflect the typical tolerance window specified by the manufacturer. In Figure S1c, we examine an extreme case in which the inductor values vary by up to 20% from their nominal values. Remarkably, even under 20% tolerance, the edge scrolls in the non-trivial phase remain nearly unaffected. For instance, while bulk scroll amplitudes may vary by as much as 0.25 V in some cases, the edge scrolls show only minimal deviation. This result highlights the robustness of the protected chaotic state, demonstrating its resilience even in the presence of significant parameter uncertainties.

We now examine the Chua-SSH circuit under large loss due to the components, wire and contact resistances. By setting the tolerance window constant as $C_1 \pm 2\%$, $C_2 \pm 2\%$, $L_a \pm 5\%$, $L_b \pm 5\%$, and $L_c \pm 5\%$, which is easily achievable for an experimental circuit, we first increase the series resistance of the inductors as $R_{L_a} = R_{L_b} = R_{L_c} = 1\,\Omega$. We observe the qualitative behavior is mostly preserved, as seen in Figure S2a. Increasing the series resistances to $R_{L_a} = R_{L_b} = R_{L_c} = 5\,\Omega$, in Figure S2b, we observe slightly more suppressed voltages, however, importantly, the edge scrolls are still distinct in the non-trivial phase. To further examine our circuit with large resistive parasitics, we set $R_{L_a} = R_{L_b} = R_{L_c} = 20\,\Omega$ and observe the same qualitative behavior in Figure S2c.

We now examine the Chua-SSH circuit under conditions of increased loss due to component parasitics, wire resistances, and contact resistances. Keeping the tolerance window fixed at $C_1 \pm 2\%$, $C_2 \pm 2\%$, $L_a \pm 5\%$, $L_b \pm 5\%$, and $L_c \pm 5\%$—a range that is easily achievable in practical experimental setups—we vary the series resistances of the inductors to simulate different loss scenarios. First, we set the inductor series resistances to $R_{L_a} = R_{L_b} = R_{L_c} = 1\,\Omega$, and observe that the qualitative behavior of the scroll amplitudes remains largely preserved, as shown in Figure S2a. Increasing the resistance further to $5\,\Omega$ in Figure S2b results in slightly more suppressed voltage amplitudes; however, the edge scrolls in the non-trivial phase remain clearly distinguishable. Finally, when the resistance is increased to $20\,\Omega$ (Figure S2c), the circuit still maintains the same qualitative features, with the protected edge dynamics remaining robust despite the significant parasitic losses.

Although these resistance values exceed the parasitic resistances specified by the manufacturers of the components used in our realistic simulations, the qualitative behavior of the circuit remains robust. In fact, the deviations shown in the right-hand plots of each panel in Figure S2 become even smaller as the series resistance increases. Overall, our simulations—which incorporate all relevant real-world experimental effects—demonstrate that protected chaos is highly feasible in practical implementations, with the key features remaining well-preserved even under large tolerance windows and significant parasitic losses.

III. THE EFFECT OF STRUCTURAL DISORDER

To investigate the robustness of chaotic dynamics induced by topology against structural disorders, we deliberately introduce disorders into bulk Chua circuits and topological coupling elements. As demonstrated in Figure 1d of the main text, chaotic trajectories transition to limit cycles when the inductance (L_c) of an isolated Chua circuit exceeds 26.06 mH, with all other parameters fixed as in the main text. In the following scenarios, we analyze how similar perturbations influence the collective dynamics in an $N = 8$ Chua-SSH lattice.

First, we introduce a localized structural disorder by altering the inductance L_c of a single Chua circuit at sublattice node A of the 4th unit cell from the default 24 mH to 30 mH, leaving all other parameters unchanged (Figure S3a). Remarkably, this localized perturbation has negligible impact on the scroll profiles and the overall non-linear phase boundary, as illustrated visually and through the phase diagram simulation.

Next, we introduce stronger disorder by simultaneously altering both the inductors L_c and capacitors C_2 within the two Chua circuits attached to sublattice nodes A and B of the 4th unit cell. Specifically, we set $L_c(4, A) = 27\,\mathrm{mH}$, $C_2(4, A) = 80\,\mathrm{nF}$, $L_c(4, B) = 30\,\mathrm{mH}$, and $C_2(4, B) = 90\,\mathrm{nF}$ (Figure S3b). While such deviations are significant enough to alter chaotic trajectories in isolated Chua circuits, the topological bulk effectively suppresses these strong disorders. The visual scroll profiles (taken at parameters corresponding to the magenta star in the associated phase diagram) remain robust, and the non-linear phase boundary, indicated by the magenta dashed line inherited from the disorder-free lattice, remains largely preserved with only minor expansion toward smaller parameter regimes.

In a third scenario, we examine multiple structural disorders at two distinct unit cells by setting $L_c(4, A) = 30\,\mathrm{mH}$, $L_c(6, A) = 28\,\mathrm{mH}$, and $C_2(6, A) = 90\,\mathrm{nF}$ (Figure S3c). Although the scroll amplitudes at the disordered nodes slightly increase compared to the other bulk nodes, the overall non-linear phase boundary remains almost identical to that of the disorder-free system.

Finally, we assess the impact of disorders in the topological couplings at the 4th unit cell. We perturb the coupling inductances by setting $L_a(4) = L_a + L_a/20$ and $L_b(4) = L_b - L_b/20$. Remarkably, the phase boundary remains nearly unaffected throughout the entire parameter space. Additionally, for the visualization of the scroll distributions, we consider an extreme case by exchanging coupling inductances, replacing $L_a(4)$ with L_b and $L_b(4)$ with L_a, effectively breaking the periodicity of the bulk couplings. Despite this drastic perturbation, as illustrated in Figure S3d, the periodicity and character of chaotic scroll profiles remain essentially unaffected.

Collectively, these four distinct disorder scenarios strongly suggest that our Chua-SSH lattice exhibits exceptional robustness against structural disorders. This inherent robustness ensures that protected chaos is readily observable experimentally, providing a promising pathway toward reliable experimental realization.

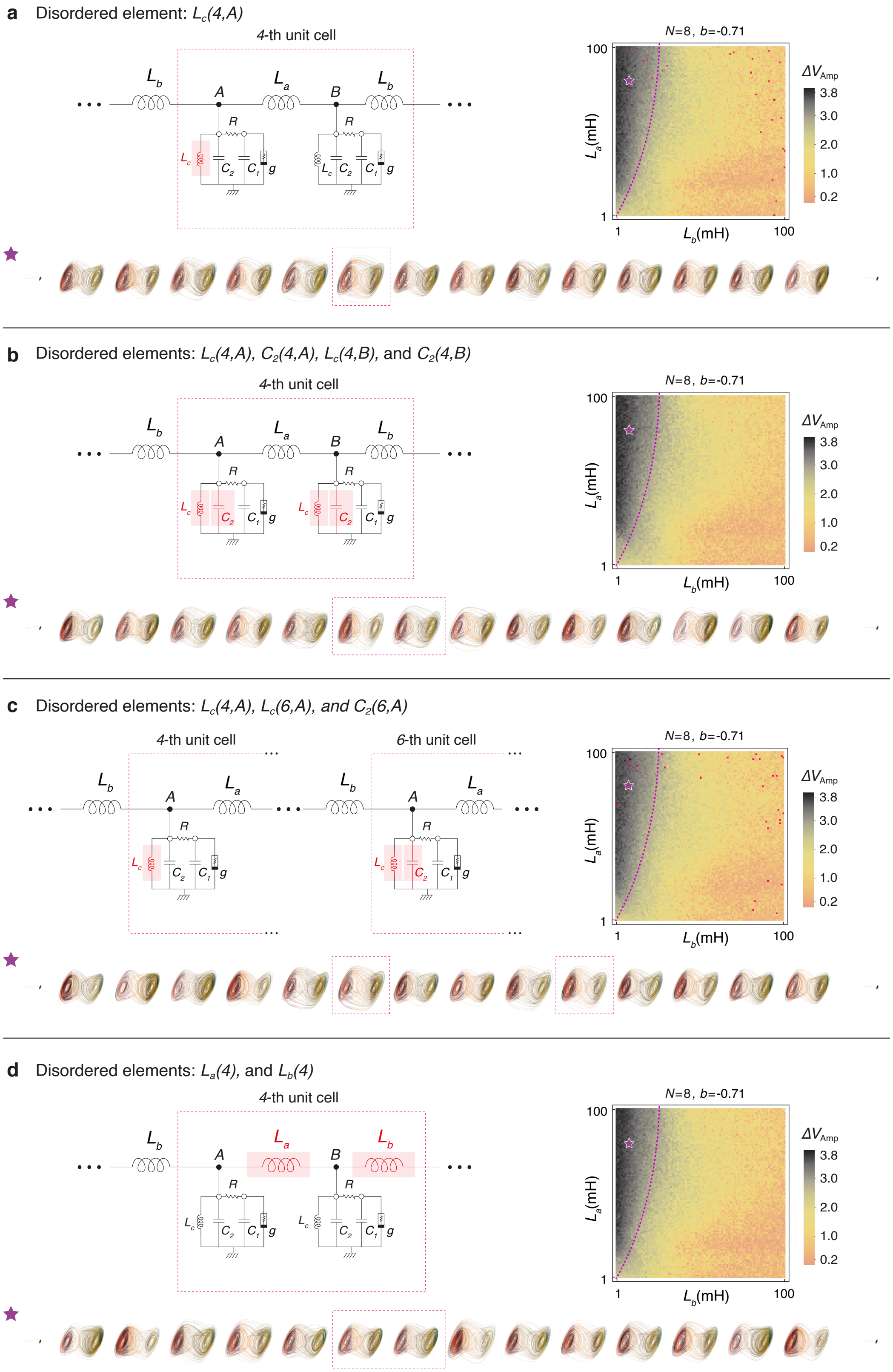

FIG. S3. **Disordered Chua-SSH circuit and chaotic dynamics. a-d** Various scenarios demonstrating structural disorders introduced into the bulk Chua circuits and topological couplings. The altered/disordered elements are highlighted in red within the dashed boxes. Corresponding phase diagrams (right column) illustrate the amplitude difference between bulk and edge scrolls (ΔV_{amp}). Magenta stars indicate the parameters $(L_a, L_b) = (80\,\mathrm{mH}, 8\,\mathrm{mH})$ selected for visualization of scroll profiles (bottom rows). The magenta dashed lines in the phase diagrams are reference phase boundaries derived from disorder-free simulations, indicating minimal impact from structural disorders. The parameters used throughout are fixed at $(L_c, C_1, C_2, R, b) = (24\,\mathrm{mH}, 10\,\mathrm{nF}, 100\,\mathrm{nF}, 1.85\,\mathrm{k\Omega}, -0.71)$.

IV. CHAOTIC PHASE TRANSITIONS OF AN ISOLATED CHUA CIRCUIT

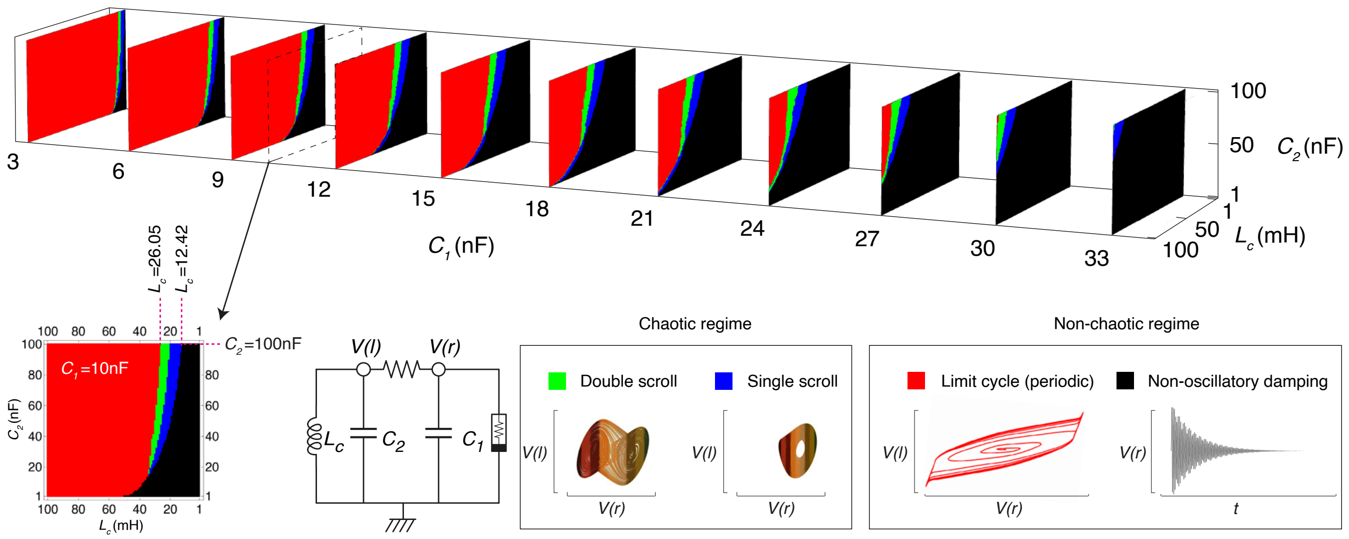

FIG. S4. **Parameter space analysis of chaotic transitions in an isolated Chua circuit.** Systematic classification of dynamical behaviors showing how variations in the energy-storage components (C_1, C_2, and L_c) trigger transitions between chaotic and non-chaotic regimes. Four characteristic regimes identified numerically are indicated by colors: double-scroll chaos (green), single-scroll chaos (blue), periodic limit-cycle (red), and non-oscillatory damping (black). The lower-left inset shows a detailed cross-section for the fixed default capacitance ($C_1 = 10\,\mathrm{nF}$), clearly marking the chaotic region boundaries at $L_c = 12.42\,\mathrm{mH}$ and $L_c = 26.05\,\mathrm{mH}$. These boundaries correspond precisely to the chaotic regime limits discussed in Figure 1d. The other parameters used are $(R, b) = (1.85\,\mathrm{k\Omega}, -0.71)$.

As topological and chaotic phases in our study are investigated primarily based on variations in the inductances of intra-cell and inter-cell inductors, we previously illustrated chaotic phase transitions by varying the inductance L_c in an isolated Chua circuit while keeping other parameters fixed (Figure 1d). Here, we expand this analysis by systematically examining parametric transitions of chaotic scroll behaviors across all three passive energy storage elements (L_c, C_1, and C_2) of an isolated Chua circuit.

To comprehensively classify chaotic and non-chaotic dynamical regimes, we define four characteristic dynamical behaviors: double-scroll chaos, single-scroll chaos, periodic limit-cycle oscillations, and non-oscillatory damping. By numerically simulating an isolated Chua circuit across a wide parameter space spanned by L_c, C_1, and C_2, we identify these four distinct behavior. In the resulting parameter diagrams (Figure S4), the double-scroll regime is marked green, the single-scroll regime is marked blue, the periodic limit-cycle regime is red, and the non-oscillatory damping regime is black.

As shown in Figure S4 in the main text, chaotic oscillations—either single scroll (blue) or double scroll (green)—occur between non-oscillatory (black) and periodic limit-cycle (red) regions. Although the parameter space supporting chaotic behavior appears relatively narrow, suitable parameter configurations for robust chaotic dynamics can still be clearly found. For instance, the analysis presented in Figure 1 demonstrated scroll transitions at fixed capacitor values ($C_1 = 10\,\mathrm{nF}$, and $C_2 = 100\,\mathrm{nF}$) while varying the inductance L_c. There, the chaotic regime was bounded $L_c = 12.42\,\mathrm{mH}$ (lower bound) and $L_c = 26.05\,\mathrm{mH}$ (upper bound). These boundaries correspond exactly to the cross-sectional diagram at $C_1 = 10\,\mathrm{nF}$ depicted in the lower-left inset of Figure S4. Thus, one can readily determine alternative parameter settings to realize protected chaos experimentally.

A. Triggering chaos through circuit parameters

To elucidate the numerical phase diagrams shown in Figure S4, we provide a compact analytical explanation of how variations in the passive elements—L_c, C_1, and C_2—lead to different dynamical behaviors in a single Chua circuit. Our aim here is to explain the sequence of transitions observed numerically. We consider the linearized dynamics

around an equilibrium point $(x_e, 0, -x_e)$, which yields the Jacobian [?]

$$J = \begin{pmatrix} -\alpha(1+m) & \alpha & 0 \\ 1 & -1 & 1 \\ 0 & -\beta & 0 \end{pmatrix}, \qquad \alpha = \frac{C_2}{C_1}, \quad \beta = \frac{R^2 C_2}{L_c}, \quad m = f_b'(x_e) \in \{a, b\}. \tag{S17}$$

Here, m corresponds to the local slope of the piecewise-linear Chua diode, which switches between a and b depending on whether the state is inside or outside the breakpoint region $|x_e| < E_p$. The eigenvalues of this matrix are determined by the characteristic polynomial

$$\lambda^3 + A\lambda^2 + B\lambda + \Gamma = 0, \tag{S18}$$

with

$$A = \alpha(1+m) + 1, \quad B = \alpha(1+m) + \alpha\beta, \quad \Gamma = \alpha\beta(1+m). \tag{S19}$$

The stability of the equilibrium is then governed by the Routh–Hurwitz conditions [? ? ? ?]. When $A > 0$, $\Gamma > 0$, and $AB - \Gamma > 0$ are all satisfied, the fixed point is stable and all trajectories decay without oscillation, which corresponds to the black region in Figure S4. The Hopf bifurcation occurs at the threshold $AB - \Gamma = 0$, where two complex-conjugate eigenvalues cross the imaginary axis. This marks the onset of a small-amplitude oscillation around the fixed point. As α or β is increased further—for example, by increasing C_2 or L_c—the amplitude of this limit cycle grows and eventually reaches the nonlinear region of the diode. Once the orbit touches one of the breakpoints, the dynamics become asymmetric and start folding back on themselves, leading to a single-scroll attractor. A further increase in parameters allows the orbit to span both breakpoints within each cycle, and the system transitions into a double-scroll attractor. This process corresponds to the emergence of more complex dynamics and marks the chaotic regime. The discriminant of the cubic equation,

$$\Delta = 18AB\Gamma - 4A^3\Gamma + A^2B^2 - 4B^3 - 27\Gamma^2, \tag{S20}$$

provides further insight. When $\Delta < 0$, the eigenvalue structure always includes one real and two complex-conjugate roots, which is necessary for oscillatory behavior. Thus, the region bounded by $\Delta < 0$ and $AB - \Gamma < 0$ supports the growth of oscillations and, ultimately, chaos. In short, increasing C_2 or L_c, or decreasing C_1, increases the energy stored in the system relative to dissipation. This change first destabilizes the fixed point via a Hopf bifurcation, then enlarges the orbit into the nonlinear regime, where the piecewise structure of the Chua diode generates either single- or double-scroll chaos. These steps follow the color-coded transitions shown in Figure S4 and provide a basic yet useful explanation of how chaos emerges in this circuit by tuning passive components.

V. RELATING THE SCROLL TRANSITIONS WITH EFFECTIVE INDUCTANCE

In an isolated Chua circuit, the voltage oscillations at the two nodes are mutually coupled, such that the evolution of one influences the other. This interplay enables an approximate characterization of the scroll type based on the amplitude of voltage oscillations at one of the nodes. In particular, under a reasonable approximation, the scroll type can be inferred from the voltage amplitude at the left node. If we recall our non-linear function (Equation (2) in the main text):

$$f_b(x) = bx + \frac{1}{2}(a-b)(|x + E_p| - |x - E_p|), \tag{S21}$$

where E_p is the breakpoint voltage of the Chua diode, which determines the transition point between the inner and outer slopes in the piecewise-linear I–V characteristic. As shown in Figure 1c and e, the slope of the nonlinear response changes at these transition points $\pm E_p$. A necessary condition for double-scroll chaotic behavior is that the voltage oscillations at the right node span both nonlinear regions, i.e., $\max|V(r)| > E_p > \min|V(r)|$, so that the system experiences alternating segments of the I–V characteristic. In this work, we consistently set $E_p = 3\,\text{V}$ and use a dimensionless outer slope of $a = -1.14$. Once the amplitude of $V(l)$ is sufficiently large, it drives the right node oscillations beyond the negative breakpoint, enabling a transition from single-scroll to double-scroll dynamics. Remarkably, we find that a sufficient condition for the onset of double-scroll behavior is that $V(l)$ exceeds E_p. This condition allows us to infer the scroll type from the left-node voltage amplitude, whose qualitative profile reflects the effective inductance at the node.

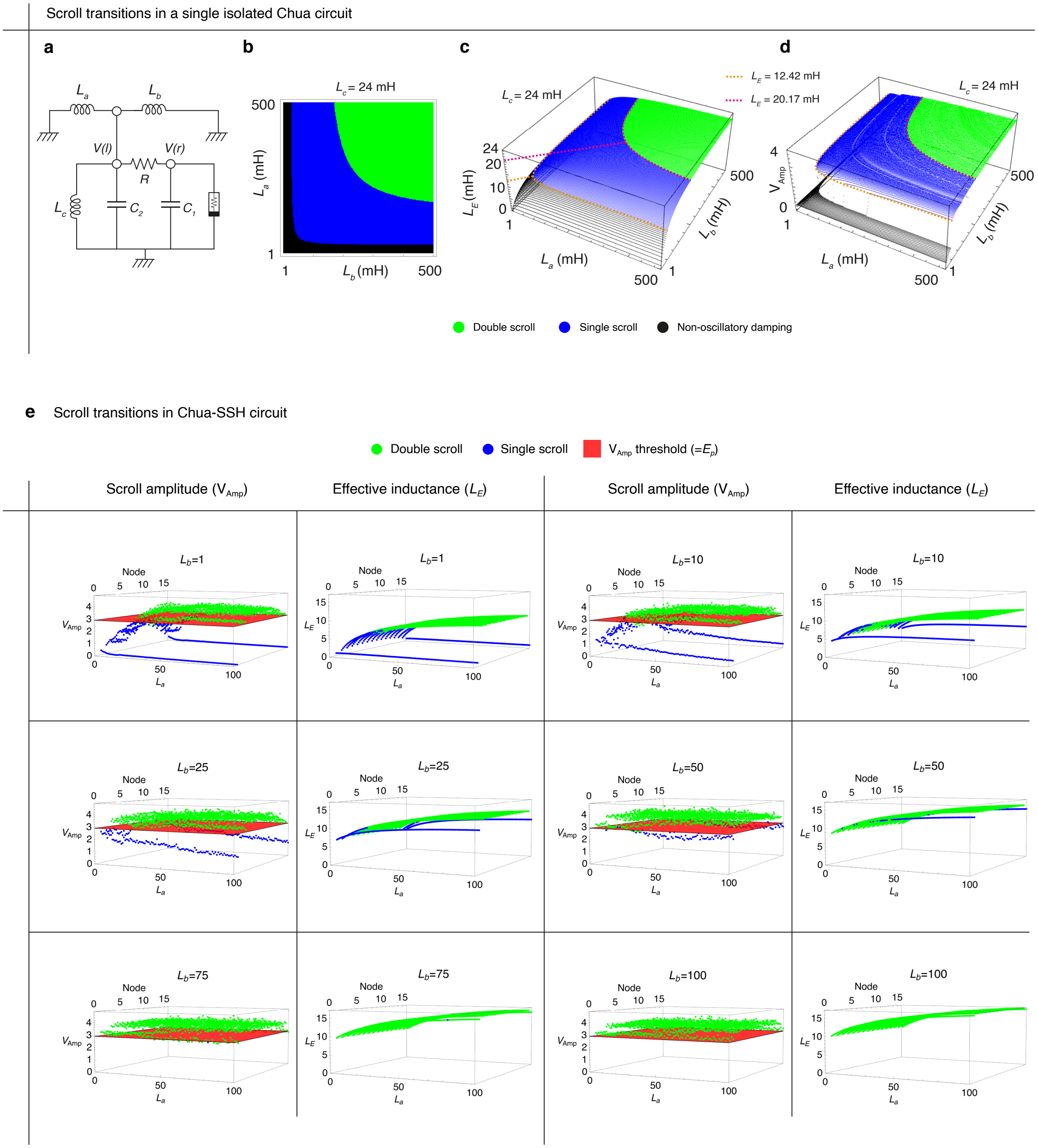

FIG. S5. **Scroll transitions in an isolated Chua circuit and the Chua-SSH array.** **a** Circuit diagram of the Chua circuit with three inductors L_a, L_b, and L_c in parallel at the left node $V(l)$ and a single inductor L_c at the right node $V(r)$. **b** Scroll state diagram showing the scroll type as a function of L_a and L_b for a single isolated Chua circuit with fixed $L_c = 24\,\text{mH}$. Blue and green regions respectively indicate single- and double-scroll dynamics. Black region is the non-oscillatory damping regime. **c** Scroll-type transition given in ‘b’ explicitly plotted as a function of the effective node inductance L_E. The transitions from non-oscillatory to single-scroll (orange dashed line) and single-scroll to double-scroll (magenta dashed line) occurs precisely at $L_E = 12.42\,\text{mH}$ and $L_E = 20.17\,\text{mH}$, identical to the single-inductor scenario (L_c only) demonstrated in Figure 1d. **d** Corresponding voltage amplitude V_{Amp} illustrating the strong correlation between the effective inductance and node voltage amplitude. **e** Scroll transitions in a Chua-SSH circuit with $N = 8$, evaluated for six different values of L_b (1 to 100 mH). For each case, we show the scroll amplitude V_{Amp} (left) and the corresponding effective inductance L_E (right) across the nodes for varying L_a. Scroll type is color-coded (blue for single-scroll, green for double-scroll). The red transparent plane indicates the threshold amplitude $V_{\text{Amp}} = E_p = 3\,\text{V}$, which separates the two scroll regimes. A clear correlation between the spatial profile of V_{Amp} and L_E emerges, reflecting the impact of effective inductance on local energy confinement and scroll behavior. The parameters used throughout are $(L_c, C_1, C_2, R, b) = (24\,\text{mH}, 10\,\text{nF}, 100\,\text{nF}, 1.85\,\text{k}\Omega, -0.71)$.

A. Scroll transitions in a single isolated Chua circuit

To elaborate on how the effective inductance influences the scroll type, we analyze a single isolated Chua circuit in which two additional inductors, representing the intra-cell and inter-cell inductors L_a and L_b, are connected between the left node and ground, as illustrated in Figure S5a. Although three inductors are connected in parallel at the left node, the scroll transition still occurs at the same effective inductance. As previously shown in the main text (Figure 1), the transition from single-scroll to double-scroll occurs at $L_c = 20.17\,\text{mH}$ when only one inductor is present. In Figure S5b, we plot the scroll types while varying L_a and L_b independently, keeping $L_c = 24\,\text{mH}$ fixed, which we adopt as the default value throughout the main text. For small values of both L_a and L_b, the circuit remains damped without sustained oscillations. As either L_a or L_b increases, the system first exhibits single-scroll chaos (blue region), followed by a transition to double-scroll behavior (green region) when one of the inductors exceeds approximately 200 mH.

The effective inductance at the left node, denoted by L_E, is simply given by

$$L_E = \left(\frac{1}{L_a} + \frac{1}{L_b} + \frac{1}{L_c} \right)^{-1}. \tag{S22}$$

In Figure S5c, we plot the scroll transition diagram as a function of L_E. The blue region, corresponding to single-scroll oscillations, transitions to the green region, corresponding to double-scroll oscillations, precisely when the effective inductance reaches $L_E = 20.17\,\text{mH}$. This is exactly the same inductance at which the scroll transition occurs when only L_c is present. This result demonstrates that the scroll type is determined by the effective inductance at the node.

Remarkably, as the effective inductance increases, the voltage amplitude at the left node also increases, and there exists a well-defined V_{Amp} threshold beyond which the system transitions from single-scroll to double-scroll, as shown in Figure S5d. The qualitative correlation between these two quantities allows us to evaluate both the scroll amplitude and scroll type based on the effective inductance.

B. Scroll transitions in our Chua-SSH circuit

We now consider an array of Chua circuits and investigate the scroll types by evaluating V_{Amp}. In Figure S5e, we display plots of V_{Amp} alongside the corresponding effective node inductance L_E. These 3D plots share axes representing the intra-cell inductance L_a and the node number. We fix $L_c = 24\,\text{mH}$ and present results for six different values of L_b. To determine the scroll types in our simulations, we utilize the Poincaré section method, coloring each data point blue to indicate a single-scroll portrait or green for a double-scroll portrait.

Notably, the scrolls remain single-scroll state below a threshold of $V_{\text{Amp}} = 3\,\text{V}$, corresponding precisely to the breakpoint voltage E_p, set to 3 V by default. For visual clarity, we include a red reference plane at 3 V in each voltage amplitude plot. As exemplified in Figure S5e for cases where $L_b < 75\,\text{mH}$, scrolls below this threshold consistently exhibit single-scroll behavior, while amplitudes exceeding 3 V transition into double-scroll states. Note that this threshold is inherently tied to the breakpoint voltage, and would thus shift accordingly if a different breakpoint voltage were employed.

Interestingly, the spatial profile of scroll amplitudes demonstrates qualitative agreement with the corresponding effective node inductance profile. This correlation allows us to infer general trends, including the evolution of bulk-edge scroll behavior. Physically, this correlation arises because higher effective inductances typically reduce coupling strength between adjacent nodes, leading to enhanced local energy confinement and thus increased amplitude at the node voltage $V(l)$. Consequently, one can reasonably estimate the scroll amplitude distribution by examining the effective inductance profile.

VI. VISUAL ILLUSTRATIONS OF SCROLL PROFILES ACROSS VARYING LATTICE SIZES

Here, we provide visual illustrations of scroll profiles corresponding to the phase diagrams presented in Figure 12 of the main text. We examine both non-trivial and trivial phases, specifically choosing parameters $(L_a, L_b) = (80\,\text{mH}, 8, \text{mH})$ for the non-trivial phase and $(L_a, L_b) = (8\,\text{mH}, 80\,\text{mH})$ for the trivial phase. As shown in Figure S6, topological suppression of edge scrolls remains prominent even for very small lattices comprising only 3 or 4 unit cells, particularly when the ratio of inductances clearly distinguishes the non-trivial from the trivial phase.

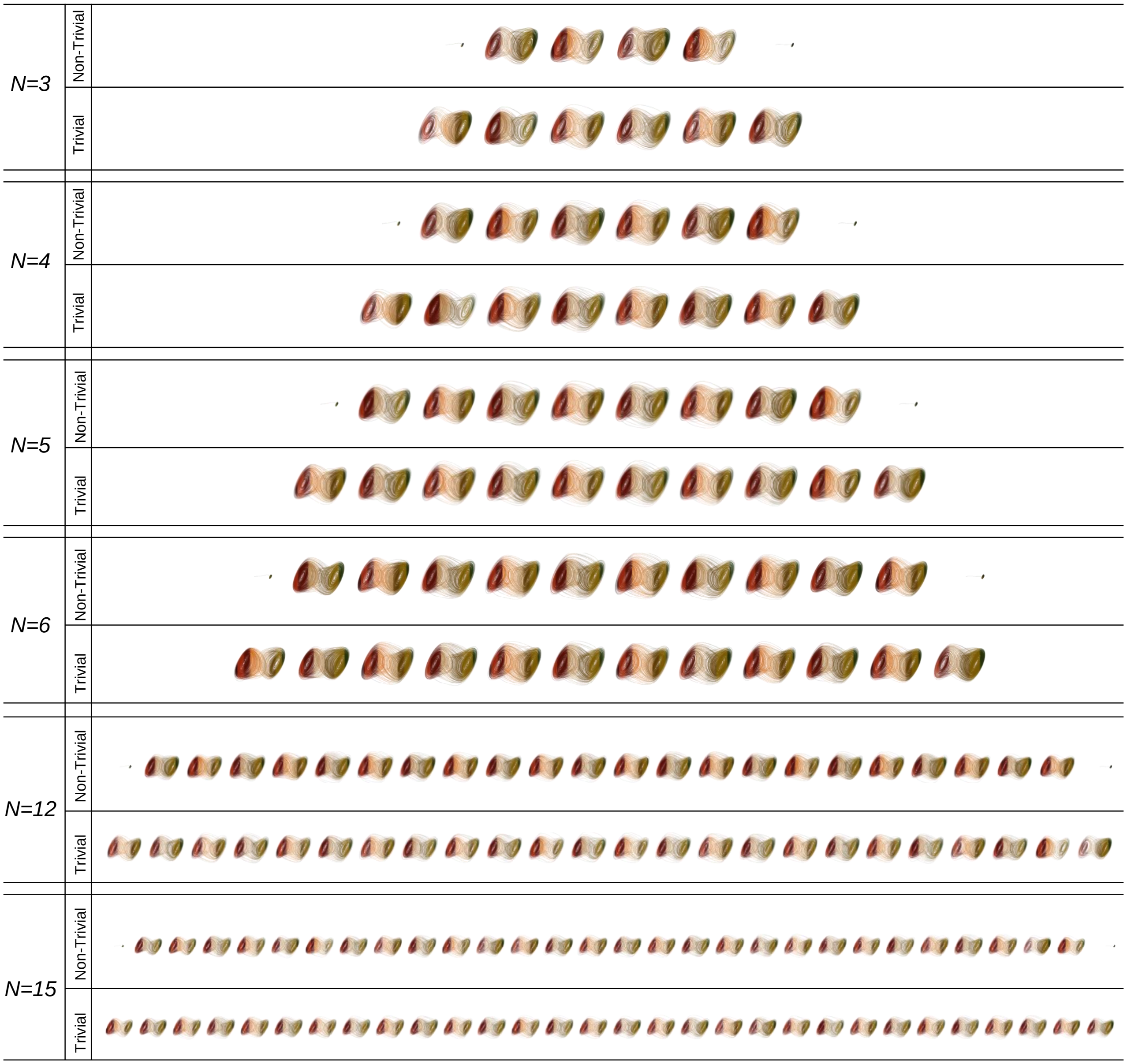

FIG. S6. **Scroll profiles illustrating the effect of lattice size on chaotic dynamics for non-trivial and trivial phases.** Scroll dynamics for varying lattice sizes $N = (3, 4, 5, 6, 12, 15)$ demonstrate topological edge suppression, even in smaller lattices ($N = 3, 4$), within the non-trivial phase. This indicates that edge suppression is robust and consistent, confirming the universality of topologically protected chaos across different system sizes. The parameters used here are $(L_c, C_1, C_2, R, b) = (24\,\text{mH}, 10\,\text{nF}, 100\,\text{nF}, 1.85\,\text{k}\Omega, -0.71)$, with inductance values set at $(L_a, L_b) = (80\,\text{mH}, 8\,\text{mH})$ for the non-trivial phase; these values are switched for the trivial phase.

VII. LINEAR DISCRIMINANT ANALYSIS (LDA) AND SUPPORT VECTOR MACHINES (SVMS)

LDA operates under the assumption that classes are linearly separable and that each class follows a multivariate normal distribution with identical covariance matrices. Mathematically, LDA seeks to find a projection vector $\mathbf{w}$ that maximizes the ratio of between-class variance to within-class variance:

$$J(\mathbf{w}) = \frac{\mathbf{w}^\top \mathbf{S}_B \mathbf{w}}{\mathbf{w}^\top \mathbf{S}_W \mathbf{w}}, \tag{S23}$$

where:

$$\mathbf{S}_B = \sum_{i=1}^{C} N_i(\boldsymbol{\mu}_i - \boldsymbol{\mu})(\boldsymbol{\mu}_i - \boldsymbol{\mu})^\top \quad \text{(Between-class scatter matrix)},$$

$$\mathbf{S}_W = \sum_{i=1}^{C} \sum_{x \in \mathcal{C}_i} (x - \boldsymbol{\mu}_i)(x - \boldsymbol{\mu}_i)^\top \quad \text{(Within-class scatter matrix)}.$$

Here, C represents the number of classes, N_i is the number of samples in class i, $\boldsymbol{\mu}_i$ is the mean vector of class i, and $\boldsymbol{\mu}$ is the overall mean vector of all classes.

In this study, LDA was applied to reduce the dimensionality of the four dimensional space $(L_a, L_b, L_E(\text{bulk}), \Delta L_E)$ to the one dimensional projection shown in Figure S7. The figure illustrates that the classes form three well-separated clusters in the reduced 1D space. This clear separation indicates that the classes are governed by well-defined linear decision boundaries, validating LDA's assumptions. Additionally, the high classification accuracy achieved further supports the notion that the underlying classes are effectively modeled by linearly separable equations within the feature space.

By verifying the existence of linear separability, we can identify the governing linear equations of the phase space through the application of Support Vector Machines (SVM). Figure S8 showcases how classes are separated in various two-dimensional projections of the phase space. Each subspace represents an equation governing the phase space and the whole phase space can be represented by combination of these conditions. It should also be noted that 3- and 4-dimensional slices exist with their own governing equations. However, due to the increased complexity with additional variables, we focus on high-accuracy two-dimensional equation combinations.

The presence of clear linear boundaries in these two-dimensional projections indicates that the classes are indeed linearly separable within these subspaces. Linear decision boundaries separating the classes are observed, demonstrating that these classes can be effectively modeled using different variables. The variables with the highest classification accuracy were selected for Equation (S24). To formalize this separability, we derive the governing linear equations using SVM, which define the hyperplanes that best distinguish between the classes. Although a two-dimensional space is sufficient for distinguishing classes in our study, the general form of the separating hyperplane in higher dimensions is expressed as:

$$\mathbf{w}^\top \mathbf{x} + b = 0, \tag{S24}$$

where $\mathbf{w}$ is the weight vector perpendicular to the hyperplane, $\mathbf{x}$ is the feature vector, and b is the bias term.

VIII. ROBUST MACHINE LEARNING EQUATIONS UNDER REALISTIC EXPERIMENTAL CONSTRAINTS

In the previous sections, we presented the experimental conditions includes various uncertainties in real-world implementations. Here, we extend our SVM formulation to ensure robustness against these perturbations commonly

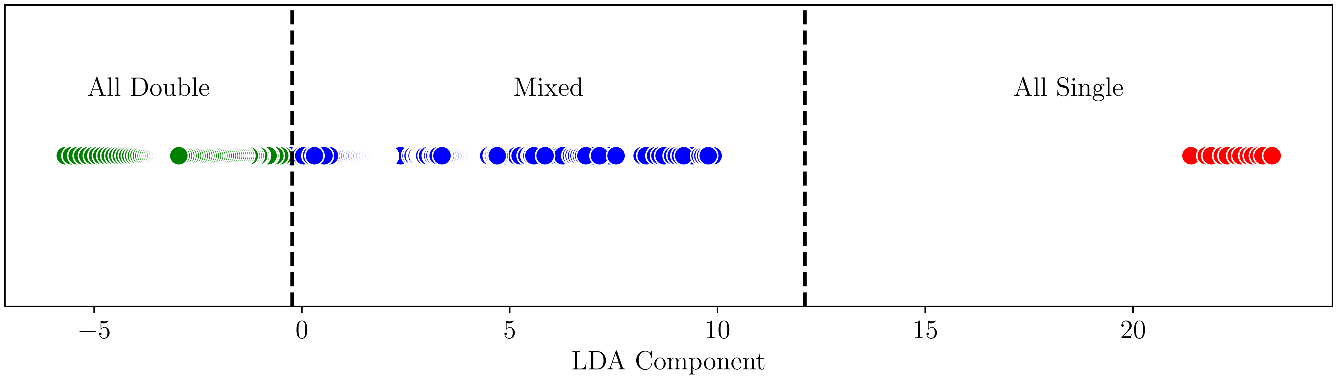

FIG. S7. **LDA Reduced 1D Space** This figure demonstrates how Linear Discriminant Analysis (LDA) distinguishes classes by maximizing the ratio of between-class variance to within-class variance. As shown, three regions are well-separated, resulting in three distinct clusters. This indicates that, overall, the class space is governed by well-defined linear equations.

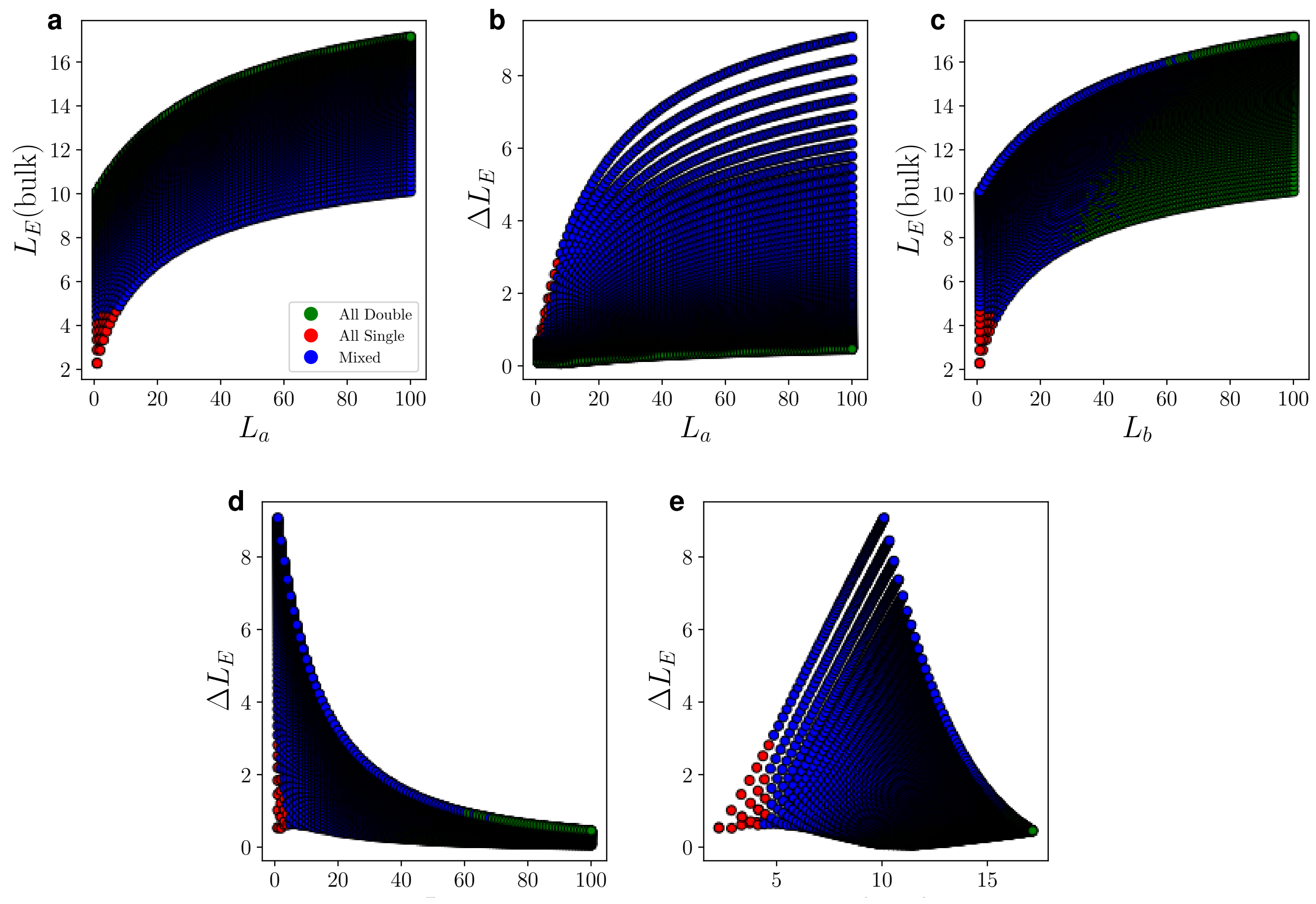

FIG. S8. **Linearly Separable 2D Phase Space Projections.** Shown are two-dimensional slices of the $(L_a, L_b, L_E(\text{bulk}), \Delta L_E)$ phase space, where linearly separable boundaries emerge across distinct subspaces. **a** $(L_a, L_E(\text{bulk}))$ separates all double-scroll cases. **b** $(L_a, \Delta L_E)$ separates all double-single cases. **c** $(L_E(\text{bulk}), L_b)$ clearly separates all three classes. **d** $(\Delta L_E, L_b)$ separates all double cases. **e** $(\Delta L_E, L_E(\text{bulk}))$ distinctly separates all three scroll types. Each of these subspaces defines an equation that governs the phase space and different combinations can be selected.

encountered in experimental settings. Specifically, we investigate the use of adversarial training to enhance robustness in practical applications.

In the adversarial training scheme, SVMs were trained on perturbed samples. Each training configuration involved perturbations along specific axes—L_E(bulk), L_b, or both—and their robustness was quantified under external noise conditions, as shown in Figure S9. Figure S9a depicts the average classification accuracy as a function of simultaneous perturbations to both L_E(bulk) and L_b. While the horizontal axis denotes the perturbation magnitude (Δ), the vertical axis shows the resulting accuracy. Each curve corresponds to a different adversarial training regime. For example, the red curve represents an SVM trained using perturbations sampled from Uniform$(-\epsilon_{\text{train}}, \epsilon_{\text{train}})$ with $\epsilon_{\text{train}} = 3$.

For each data point $\mathbf{q}_i$ shown in Figure 4a, we perform the following steps during training:

1. Generate a set of 25 perturbed samples:

$$\mathbf{q}_{i,j} = \mathbf{q}_i + \boldsymbol{\epsilon}_{i,j}, \quad \boldsymbol{\epsilon}_{i,j} \sim \text{Uniform}\big(-\epsilon_{\text{train}}, \epsilon_{\text{train}}\big), \quad j = 1, 2, \ldots, 25.$$

2. Identify the adversarial sample $(\hat{\mathbf{q}}_i)$ by selecting the perturbation that maximizes the SVM loss ℓ_{SVM} for the corresponding label y_i:

$$\hat{\mathbf{q}}_i = \underset{1 \le j \le 25}{\arg\max}\, \ell_{\text{SVM}}(\mathbf{q}_{i,j}, y_i) = \underset{1 \le j \le 25}{\arg\max} \left\{ \max\Big(0,\, 1 - y_i \left(\mathbf{w}^\top \mathbf{q}_{i,j} + b\right)\Big) \right\}.$$

3. Use $\hat{\mathbf{q}}_i$ as the training sample for the original data point $\mathbf{q}_i$ to enhance model robustness.

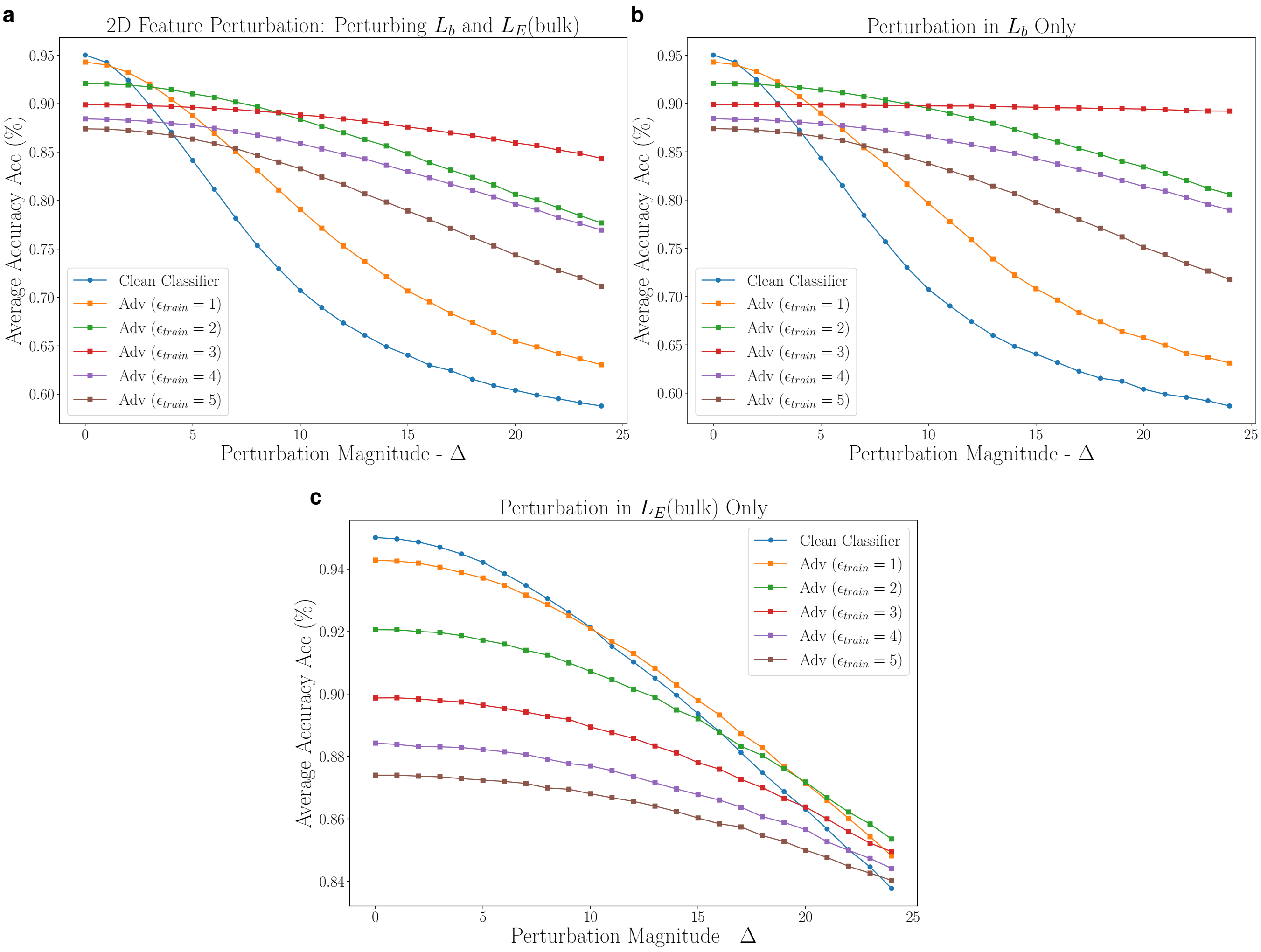

FIG. S9. **Robustness of adversarially trained support vector machines (SVMs) against perturbations. a)** Average classification accuracy of SVMs trained with varying adversarial perturbation amplitudes, evaluated under simultaneous perturbations in both dimensions $(L_E(\text{bulk}), L_b)$. Each curve corresponds to a different adversarial training regime, where during training, 25 perturbed versions of each original data point were generated using noise sampled independently from $\text{Uniform}(-\epsilon_{\text{train}}, \epsilon_{\text{train}})$, and the one yielding the highest SVM loss was selected. At test time, 50 new perturbations were applied to each data point in Figure S9a of the main text, and accuracy was averaged across these realizations. Adversarially trained SVMs—such as the red curve trained with $\epsilon_{\text{train}} = 3$—maintain accuracy above 85% even under test noise drawn from $\text{Uniform}(-\Delta = 25, \Delta = 25)$, indicating that adversarial training effectively enhances model robustness. **b)** and **c)** Classification accuracy under perturbations applied individually to L_b and $L_E(\text{bulk})$, respectively. These results demonstrate that aligning the adversarial training range with anticipated experimental uncertainties enables an optimal trade-off between accuracy and robustness, ensuring reliable classification in realistic, noisy environments.

During testing, for each original data point $\mathbf{q}_i$, we sample 50 new perturbations:

$$\mathbf{q}'_{i,k} = \mathbf{q}_i + \boldsymbol{\epsilon}'_{i,k}, \quad \boldsymbol{\epsilon}'_{i,k} \sim \text{Uniform}\big(-\Delta, \Delta\big), \quad k = 1, 2, \ldots, 50,$$

where Δ denotes the test perturbation magnitude. The final classification accuracy is computed as the average performance over 50 perturbed instances for each data point.

While accuracy generally decreases as perturbation strength increases, models trained with these worst-case scenarios demonstrate significantly greater resilience—indicating that noise-injected training enhances robustness without a substantial loss in performance. For example, the red curve illustrates that classification accuracy remains consistently high—between 90% and 85%—across a broad range of perturbations sampled from $\text{Uniform}(-25, 25)$, highlighting the model's robustness in the presence of large perturbations. Moreover, by aligning the training perturbation range with the expected experimental uncertainty in each individual dimension, classification performance can be optimized for specific conditions, leading to more reliable phase-profile determinations. For instance, although the red curve

demonstrates the highest robustness over an extended perturbation range (Uniform($-25, 25$)), if the experimental uncertainty is known to be Uniform($-10, 10$), the green curve—trained with adversarial perturbations in the narrower range Uniform($-2, 2$)—emerges as the best choice.

Similarly, Figure S9b and Figure S9c illustrate how model performance responds to perturbations applied individually to L_b and L_E(bulk), respectively. These results demonstrate that adversarial training can be effectively tailored to match the distinct noise characteristics of each dimension. For example, when L_E(bulk) is relatively stable—perturbed by less than Uniform($-5, 5$)—the clean classifier (blue curve in Figure S9c) already performs optimally, so no additional adversarial training is required in that dimension. In contrast, when L_b is subject to more substantial noise drawn from Uniform($-10, 10$), the most effective strategy is to apply adversarial training exclusively along the L_b dimension using perturbations from Uniform($-2, 2$), since the green curve in Figure S9b ($\epsilon_{\text{train}} = 2$) achieves the highest accuracy in this regime. This analysis allows for the optimal choice of adversarial training regime when the uncertainties in each dimension are known. Lastly, a comparison between Figure S9b and Figure S9c reveals that perturbations in L_b degrade model performance more significantly than those in L_E(bulk), highlighting the importance of dimension-specific training strategies.

In summary, adversarial training provides a systematic approach for enhancing model robustness to experimental uncertainties. This framework not only improves classification reliability but also enables the design of classifiers specifically optimized for the noise profiles encountered in different experimental settings.

IX. EVOLUTION OF PHASE BOUNDARIES

The acquired linear boundaries provide a robust quantification of the evolving k-means clusters. Additionally, the consistent alignment between the high-pass filtered space, the k-means boundaries, and the linear decision boundaries further supports the validity of the applied methodology. The final column of Figure S10 highlights the real space evolution, corresponding to the results shown in Figure 14e.

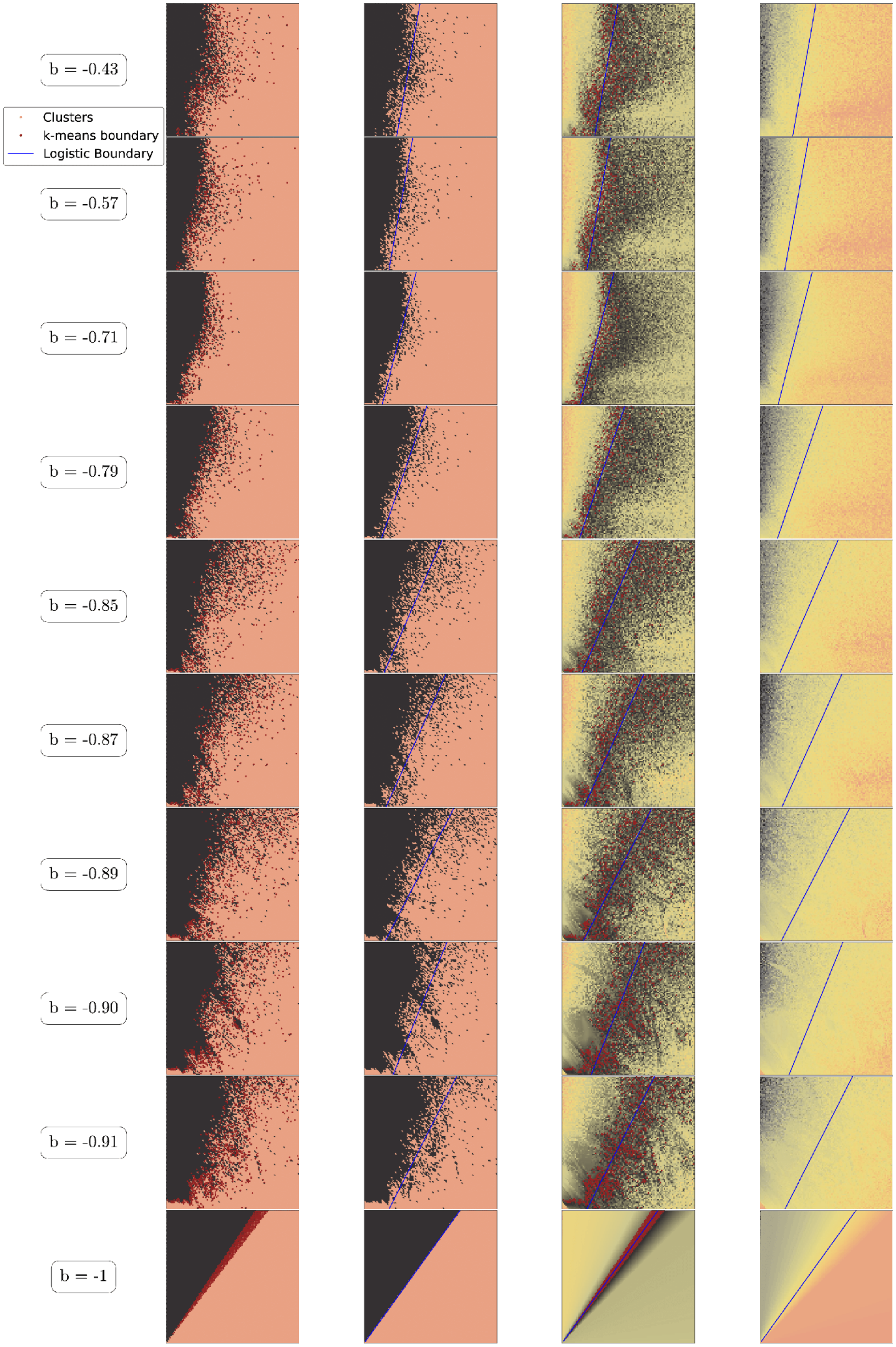

FIG. S10. **Determining the phase boundaries.** This figure presents various processed representations of phase spaces for different b values. The leftmost column depicts the phase boundaries and clusters identified by k-means clustering. The second column shows the linear decision boundaries derived from logistic regression, marking the optimal separation between clusters. The third column overlays the resulting linear decision boundary and k-means boundary on the high-pass filtered phase space. Finally, the fourth column shows how the decision boundary maps onto the real phase space.
